# GSNCASCR: An R Package to Identify Differentially Co-Expressed Curated Gene Sets with Single-Cell RNA-Seq Data

**DOI:** 10.3390/ijms26104771

**Published:** 2025-05-16

**Authors:** Shouguo Gao, Haoran Li, Zhijie Wu, Hiroki Mizumaki, Sachiko Kajigaya, Neal S. Young

**Affiliations:** Hematopoiesis and Bone Marrow Failure Laboratory, Hematology Branch, National Heart, Lung, and Blood Institute, National Institutes of Health, Bethesda, MD 20892, USA

**Keywords:** single-cell RNA-seq, differential co-expression, pathway analysis

## Abstract

(1) Differential co-expression analysis between two phenotypes with a known gene set helps to uncover gene regulation alterations. (2) GSNCASCR uses CSCORE to estimate the gene pair correlations for network reconstruction and GSNCA to quantify the structure changes of co-expression networks of the predefined gene sets. It also ranks genes based on their “importance” in the weighted network. The method is implemented with free R software (version 0.1.0, available on GitHub), allowing users to analyze their data with the help of demo vignettes included in the package. (3) With analysis of both simulated and real datasets, we demonstrate that the statistical tests performed with GSNCASCR are able to identify differentially co-expressed gene sets with higher precision than tests with Gene Set Co-Expression Analysis (GSCA, version 1.1.1) and Gene Sets Net Correlations Analysis (GSNCA, version 1.42.0). Specifically, GSNCASCR achieved an AUC value of 0.985, while GSNCA and GSCA achieved 0.817 and 0.893, respectively, when positive and negative pathways are defined as having more than 40% and less than 20% co-expressed gene pairs in the simulated data, respectively. Furthermore, across simulated data with varying noise levels, pathway sizes, and positive/negative pathway definitions, GSNCASCR consistently performs best in over 90% of scenarios, as evaluated by AUC values. With an available COVID-19 dataset, we show CD4^+^ T cell dysfunction in severe COVID-19 as TNF-α/TNF receptor 1-dependent immune pathways. In the weighted network of a gene set of *IFN-γ*, *IFITM3* was identified as a hub gene, which has been evidenced by a genome-wide association study and functional studies. (4) We developed a bioinformatics tool, GSNCASCR, that analyzes differentially co-expressed pathways with single-cell RNA-sequencing data and also evaluates the importance of the genes within pathways. This tool combines the advantages of two algorithms, enabling the quantification and examination of cell type-specific co-expression changes within pathways. The package allows for the analysis of shared and unique disease-affected pathways across different cell types.

## 1. Introduction

Differential co-expression analysis examines diseases and phenotypic variations by finding gene pairs whose co-expression patterns vary across conditions. The simplest differential co-expression analysis compares clusters of co-expressed genes under different conditions. Many methods have been developed to identify differentially co-expressed gene modules [1].

Gene set analysis methods, such as the well-known Gene Set Enrichment Analysis (GSEA) [2], examine the overall differential expression of sets of related genes (pathways). They enhance statistical power and aggregate prior biological knowledge. One motivation to test pathways is based on the idea that complex diseases are rarely consequences of abnormalities in a single gene but a result of changes in a set of related genes. Despite their success, GSEA and similar approaches do not identify important classes of differentially regulated pathways, such as groups of differentially co-expressed genes.

Other methods that test differential co-expression for a predefined collection of gene sets have been developed [3,4]. These methods vary in how they quantify co-expression between genes, measure changes in the co-expression of a group of genes, and cluster genes. The problem of measuring differential co-expression of a given gene set is formulated by the Gene Sets Co-expression Analysis (GSCA) [3] and Gene Sets Net Correlations Analysis (GSNCA) methods [4]. For example, GSCA calculates the Pearson correlation and aggregates the pairwise correlation differences between two conditions [3]. These methods are mainly used for microarray or bulk RNA sequencing (bulk RNA-seq) data and, more occasionally, for single-cell RNA-sequencing (scRNA-seq) data. Advances in scRNA-seq technology have enabled direct inference of co-expression in specific cell types [5,6].

Pearson correlation is often used to calculate gene co-expression, but co-expression derived from scRNA-seq showed lower functional connectivity than that from bulk RNA-seq [7], due to batch effects or incomplete transcriptome coverage inherent in current scRNA-seq protocols [7]. Thus, the usual correlation approach cannot be applied to scRNA-seq, and new approaches have been proposed to capture gene co-expression from scRNA-seq data [6,7]. Several methods have been developed recently to better capture co-expressions from scRNA-seq data, including PIDC [8], locCSN [9], scLink [10], SpQN [11], CoAM [12], DeepCSCN [13], and others. For instance, scLink calculates correlations of gene pairs and applies a penalized, data-adaptive likelihood method to examine sparse dependencies among genes and to construct sparse gene co-expression networks [10]. PIDC utilizes partial information decomposition based on multivariate information theory to quantify statistical dependencies among genes and infer gene networks [8]. scDiffCoAM considers different association metrics or additional adjustments when inferring co-expressions from scRNA-seq [12]. DeepCSCN can infer co-expression at the whole sample level and build cell-type-specific co-expression networks, demonstrating significant improvements over many existing methods [13]. However, these methods primarily focus on gene–gene co-expression and network inference without incorporating curated pathway information, resulting in difficulties for biologists to interpret and infer a biological hypothesis. GSCA and GSNCA are two tools that allow pathway level analysis, but were developed before RNA-seq technology came to the fore [3,4].

For a typical single-cell experiment, there is a substantial variation of sequencing depth across cells. As a result, gene co-expression measured via correlations of Unique Molecular Identifier (UMI) counts across cells can be confounded by varying sequencing depths, resulting in inflated false positive findings for co-expressed gene pairs. Measurement errors in the count data add an additional challenge in inferring co-expression levels, as these errors tend to attenuate correlation estimates to different degrees for genes with distinct expression levels. Several methods have been recently developed to better capture gene co-expression from scRNA-seq data than a simple normalization-based approach: they consider different association or additional adjustments when inferring co-expression. Recently, Circuit Switching-Core (CS-CORE) identified gene co-expression that was more reproducible across independent datasets and is more enriched with known transcription factor–target gene pairs than other methods [6].

In this manuscript, we integrate GSCNA and CS-CORE and develop a new package, Gene Sets Net Correlations Analysis with Single-Cell RNA-seq (GSNCASCR), available at GitHub. Performance was evaluated with both simulated and real experimental datasets. We implemented the algorithms as a publicly available package, allowing users to freely download, use, and modify it. Several demo vignettes were created to guide users for data preparation, analysis, results summary, and visualization.

## 2. Results

**Figure 1:**
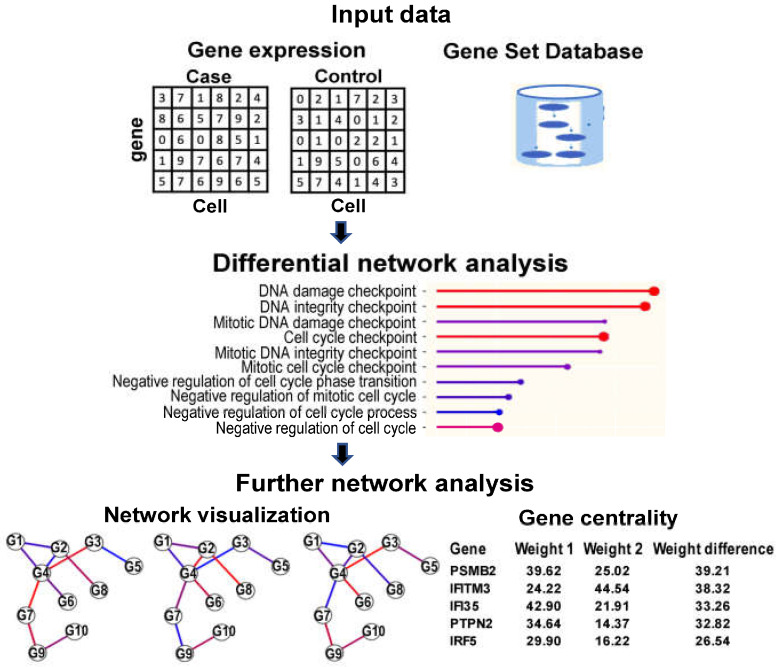
Overview of the GSNCASCR package. The package receives scRNA-seq expression matrix of case and control, and a collection of pathways as input data. It constructs two gene co-expression networks for each pathway and tests the network difference between case and control. The software allows for examining each pathway by visualizing the gene co-expression networks (case, control, and difference) and ranking the genes according to their network importance.

The GSNCASCR R package (version 0.1.0) compares gene co-expression networks in terms of their structural properties. The construction of co-expression networks, the graph spectral analysis, and main features of the package are described below:

The GSNCASCR package receives a gene expression matrix, cell labels, and a collection of gene sets as input data. Then, it randomly selects the same number of cells of two conditions, constructs two gene co-expression networks for each gene set, and tests the equality in the network structural features between two biological conditions (Figure 1). The software allows the user to further analyze each gene set by visualizing the gene co-expression graphs, ranking the genes according to their “importance” in the gene set network, and performing standard single gene differential expression analysis.

Our package integrates the advantages of CS-CORE and GSNCA. CS-CORE is able to identify the gene co-expression of each population, while the co-expression derived from bulk RNA-seq is mainly from cell type composition [6], important when there are no good surface markers for flow cytometry sorting cell populations. GSNCA captures the topological difference between co-expression networks of two conditions. The results from GSNCASCR are able to prioritize trait-relevant cell types and candidate genes.

We used both simulation experiments and analyses of biological data to evaluate the performance of GSNCASCR.

### 2.1. GSNCASCR Package

Most co-expression analyses have been performed on bulk samples, which consist of mixed cell types. Utilizing the variancePartition tool [14], we analyzed the expression variance for each gene in the COVID-19 dataset, finding that cell type was the largest contributor to gene expression (Figure S1). This indicates that changes in co-expression between two conditions are largely influenced by variations in cell type composition in bulk samples. CS-CORE demonstrates a significant advantage in this context, as it is specifically designed to infer co-expression changes within one single cell type [6]. Although profiling sorted cells can achieve similar insights, cell sorting presents challenges due to the lack of high-quality antibodies. Even if feasible, this process can be tedious and susceptible to technical artifacts [6].

The GSNCASCR package is a tool to analyze gene co-expression networks. It receives gene expression data and a predefined collection of gene sets, from which it performs differential network analysis. The software also includes further analytics of a gene set, such as network visualization, centralities of the genes that belong to the set, and the standard single gene differential expression analysis, as shown in Figure 1. In the next paragraphs, we describe briefly the input, output, and main features of the package. For a detailed tutorial and manual, refer to https://github.com/shouguog/GSNCASCR (accessed on 14 May 2024) (examples are available as vignettes in the same website).

### 2.2. Simulation

To evaluate the statistical powers of GSNCASCR, GSCA, and GSNCA methods, we generated simulated datasets with scDesigner2 (version 0.1.0).

**Figure 2:**
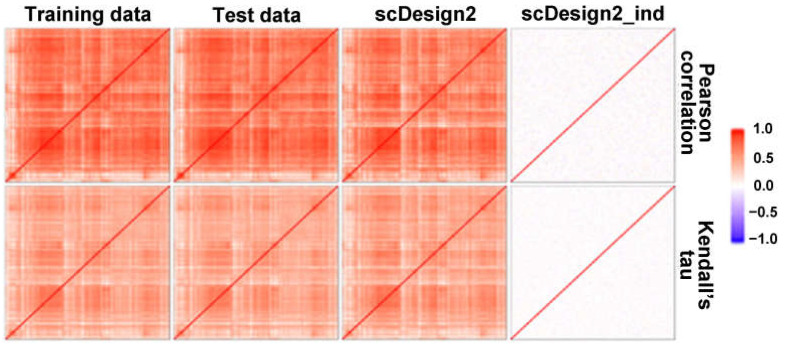
The simulation of scRNA-seq data by scDesign2 with maintaining correlation (column 3) or not maintaining correlation (column 4). Pearson (**top**) and Kendall’s tau (**bottom**) correlations between gene pairs were shown. Highly correlated gene pairs in training data are used to create negative/positive pathways.

With scDesign2, and based on the dataset of mouse_sie_10x.rds in this package, we simulated one dataset, maintaining the gene co-expression and another without co-expression (Figure 2). We extracted the highly corelated gene pairs in the training dataset and manually created gene sets with different numbers of co-expressed gene pairs. Datasets with >40% and <20% gene pairs were defined as positive and negative datasets, respectively [15].

Steps to generate the simulated data are illustrated in Figure S2. (1) Based on the training dataset, scDesign2 was used to generate two single-cell datasets: one that maintains co-expression and another that does not. (2) We manually created pathways of varying sizes (20, 40, 60, 80, and 100) by selecting different numbers (n) of highly correlated gene pairs from the initial training dataset. The remaining genes were randomly selected to complete the pathways (pathway size–number of correlated genes). (3) We ran the GSNCASCR, GSNCA (version 1.42.0), and GSCA (version 1.1.1) software tools to compare datasets with and without maintained co-expression. Positive pathways exhibited more co-expression changes than negative pathways. (4) We used AUC values to evaluate the performance of the three tools.

The w value, as described in Equation (3) of Materials and Methods, is utilized to quantify the differences between networks under two conditions. Permutations were obtained by shuffling cell labels. We observed that the w values from these permutations follow a normal distribution (Shapiro–Wilk test), as shown in Figure S3. Consequently, we compared our observed results with the permutation-derived distribution to estimate p-values.

**Figure 3:**
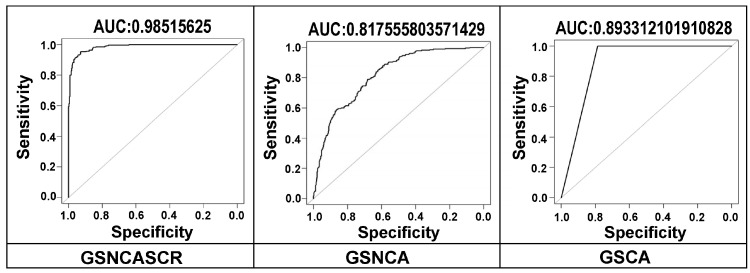
ROC curves for the three differential co-expression analysis tools using simulated data with default parameters. No extra errors were added to the simulated data.

We used simulated data to compute true sensitivities and precision of the tools for detecting co-expression alteration pathways. Receiver operating characteristic (ROC) curves, using the simulated data (>40% and <20% gene pairs for positive and negative pathways, respectively), are shown in Figure 3. GSNCASCR shows the highest area-under-the-curve (AUC) value, indicating the best performance among the three tools tested.

Average true positive rates (TPRs, sensitivities), false positive rates (FPRs), precision, and accuracy of the tools are given in Table 1. We defined TPs as truly called differentially co-expressed pathways and FPs as the pathways called significant but not differentially co-expressed pathways. Similarly, true negatives (TNs) were defined as pathways that were not truly differentially co-expressed and were not called significant, and false negatives (FNs) were defined as pathways that were truly differentially co-expressed but were not called significant.

As seen in Table 1, GSNCASCR identified the gained, highest identification accuracy at 0.79 and precision at 1.00. In comparison, GSCA identified the greatest number of truly differentially co-expressed pathways but also introduced the highest number of false positives (high false positive rate), which resulted in a low identification accuracy at 0.69. GSNCA identified the smallest number of truly differentially co-expressed pathways though it introduced a small number of false positives, which resulted in a low identification accuracy at 0.73.

Adjusting the cutoff criteria used to define positive and negative pathways in simulated data will produce different AUC values for the three software tools. More stringent cutoffs are anticipated to result in higher AUC values because they create a clearer distinction between positive and negative pathways. We experimented with various cutoff types and discovered that GSNCASCR consistently outperformed the others in approximately 90% of the cutoff combinations. AUC values using the criteria of greater than 40% for positive and less than 40% for negative pathways, without any grey area, are presented in Supplementary Figure S4.

Due to the inherent noise in scRNA-seq data, it is essential to assess the impact of dropout and noise. Our simulated dataset is based on actual scRNA-seq data, which naturally includes dropout and noise. To further mimic these conditions, we introduced two types of data corruption: (a) increasing the number of zeros (dropouts) and (b) adding noise [16,17]. Dropouts were applied by setting low expression values to zero with a higher probability, varying the fraction of zeros from 0.1 to 0.7. For noise addition, we randomly increased expression values by 30–50% or decreased them by 20–40%, with probabilities ranging from 0.1 to 0.7.

AUC values decrease as dropout rates and noise levels increase (Figure S5). Therefore, conducting quality control and removing low-quality cells are essential steps before analysis. Further improvements in performance can be achieved by screening and eliminating technical noise in scRNA-seq data [18].

### 2.3. Biological Experiments

To examine performance in a real dataset, we firstly applied GSNCASCR to a scRNA-seq dataset from human peripheral blood mononuclear cells (PBMC) of seven hospitalized patients with SARS-CoV-2 and six healthy donors to identify biological pathways differentially regulated in COVID-19 patients [19]. Gene sets were taken from the Hallmark pathway sets of the molecular signature database (MSigDB, https://www.gsea-msigdb.org/gsea/index.jsp (accessed on 16 May 2023)) where a total of 50 pathways are present. We also used gene sets taken from the Gene Ontology (GO) biological process pathways from MSigDB. Pathways with <40 or >1000 genes were discarded and the resulting datasets comprised 7000 genes and 1026 pathways to analyze [2].

Approximately 80% of gene expressions follow a normal or log-normal distribution [20]. Given that different genes may exhibit varying distributions, selecting a normal distribution is often the best approach, as it fits most genes well, which is required by CS-CORE. We used the same real dataset as referenced in the paper of CS-CORE [6]. Consequently, the dataset met the requirements for CS-CORE’s measurement model.

The top 20 complete lists of pathways identified in CD4^+^ T cells are provided in Table 2. Pathways found by GSNCASCR approaches were mainly immune related, including HALLMARK_INTERFERON_GAMMA_RESPONSE, HALLMARK_INTERFERON_ALPHA_RESPONSE, HALLMARK_TNFA_SIGNALING_VIA_NFKB, HALLMARK_COMPLEMENT, HALLMARK_IL2_STAT5_SIGNALING, and HALLMARK_IL6_JAK_STAT3_SIGNALING. GO terms datasets contained many more pathways, and again most of pathways were immune related, with top pathways of GOBP_DEFENSE_RESPONSE_TO_SYMBIONT, GOBP_CYTOPLASMIC_TRANSLATION, GOBP_REGULATION_OF_VIRAL_GENOME_REPLICATION, GOBP_POSITIVE_REGULATION_OF_IMMUNE_SYSTEM_PROCESS, GOBP_RESPONSE_TO_VIRUS, GOBP_AMIDE_BIOSYNTHETIC_PROCESS, GOBP_PEPTIDE_BIOSYNTHETIC_PROCESS, GOBP_PROTEIN_ACETYLATION, GOBP_NEGATIVE_REGULATION_OF_VIRAL_PROCESS, and GOBP_ANTIGEN_RECEPTOR_MEDIATED_SIGNALING_PATHWAY.

**Figure 4:**
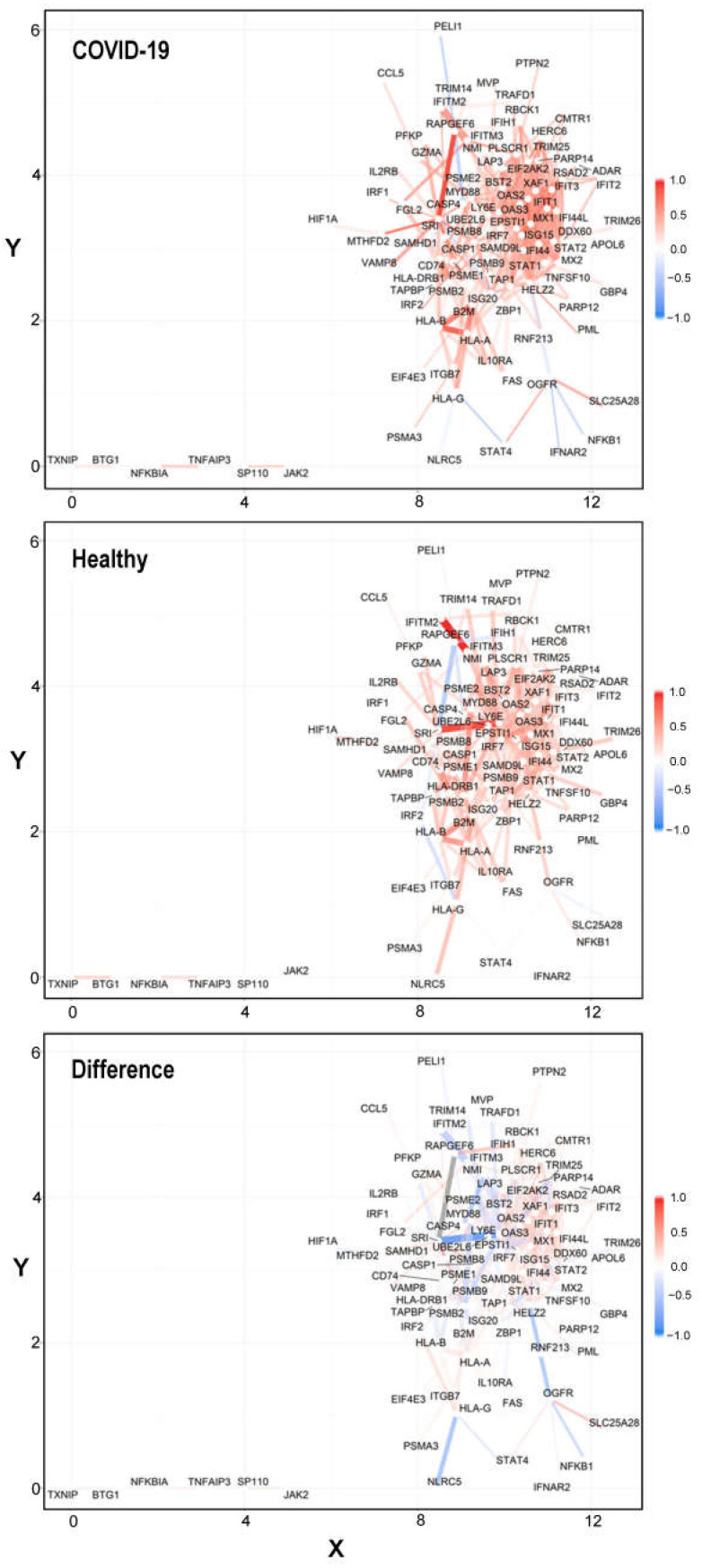
The weighted networks in COVID-19, healthy donors, and their difference in CD4^+^ T cells. Red and blue indicate positive or negative correlations, respectively. Only edges with |r1 − r2| > 0.2 were retained. The correlations of r1 (COVID-19), r2 (Healthy), and r1 − r2 (Difference) were used to define the colors and widths of the network edges.

Co-expression networks for healthy, control, and differential conditions are shown in Figure 4 (figure with .pdf format is available in Supplementary File S2). Additionally, network visualizations in ggraph format for control, COVID-19, and differential networks are available in Supplementary File S3 for further examination.

In both B cells (Table 3 and Table S1) and CD4^+^ T cells, HALLMARK_INTERFERON_GAMMA_RESPONSE, HALL-MARK_INTERFERON_ALPHA_RESPONSE, and HALL-MARK_TNFA_SIGNALING_VIA_NFKB were all among the top identified pathways. This was consistent with our understanding of COVID-19. Cytokines, such as interleukin-6 (IL-6), interleukin-1 (IL-1), interleukin-17 (IL-17), and tumor necrosis factor-alpha (TNF-α) play a significant role in lung damage in acute respiratory distress syndrome patients through impairment of respiratory epithelium. Cytokine storm is defined as acute overproduction and uncontrolled release of proinflammatory markers, locally and systemically [21].

Multiple studies have highlighted dysregulation of complex networks of peripheral blood immune responses in COVID-19, using scRNA-seq analysis [20,22,23]. Monocytes, dendritic cells, natural killer (NK) cells, T cells, and B cells are all reported to relate to disease severity, while a dysregulated interferon (IFN) response, which has a key role in innate immune response, is associated with disease pathogenesis and severity. Rare loss-of-function mutations in IFNAR2 are associated with severe COVID-19 and many other viral infections. Administration of IFN might reduce the likelihood of critical illness in COVID-19 but could not distinguish if such a treatment might be effective during disease progression of COVID-19. Several of these loci corresponded to previously documented associations to lung or autoimmune and inflammatory diseases [24].

High levels of proinflammatory cytokines such as TNF-α and interleukins are produced by innate immune cells to fight SARS-CoV-2 infections. Cytokine-mediated inflammatory events are also linked to detrimental lung injury and respiratory failure, which can result in patients’ deaths. TNF-α is among the early cytokines produced to mediate proinflammatory responses and enhance immune cell infiltration in response to SARS-CoV-2 infections.

We then examined differential expressed pathways in CD8^+^ T cells, and the results are shown in Table 4.

Surprisingly, in CD8^+^ T cells, top pathways were not immune-related, although COVID-19 causes several immune-related complications, such as lymphocytopenia and cytokine storm. Our results are consistent with a study that showed that SARS-CoV-2-infected human CD4^+^ T helper cells, but not CD8^+^ T cells, are present in blood and bronchoalveolar lavage CD4^+^ T helper cells of severe COVID-19 patients. Also, previous studies showed SARS-CoV-2 spike glycoprotein directly binds to the CD4 molecule, which in turn mediates the entry of SARS- CoV-2 into CD4^+^ T helper cells, leading to impaired CD4^+^ T cell functions and cell death. SARS-CoV-2-infected CD4^+^ T helper cells express elevated IL-10, which is associated with viral persistence and disease severity. Thus, CD4-mediated SARS-CoV-2 infection of CD4^+^ T helper cells may contribute to a poor immune response in COVID-19 patients [21]. Similarly, with GO biological process terms, the top terms were TELOMERE related, DNA replication, and protein synthesis and localization (Table S2). In contrast, in CD4^+^ T cells, most of the top terms were immune related (Table S3), such as GOBP_DEFENSE_RESPONSE_TO_SYMBIONT, GOBP_CYTOPLASMIC_TRANSLATION, GOBP_REGULATION_OF_VIRAL_GENOME_REPLICATION, GOBP_POSITIVE_REGULATION_OF_IMMUNE_SYSTEM_PROCESS, GOBP_RESPONSE_TO_VIRUS, GOBP_AMIDE_BIOSYNTHETIC_PROCESS, GOBP_PEPTIDE_BIOSYNTHETIC_PROCESS, and GOBP_PROTEIN_ACETYLATION.

Our results also revealed the importance of identifying cell-type-specific co-expression, which is more enriched for biorelevant pathways [2], as most gene–gene correlations were brought by the cell-type specificity of gene expression. For example, two genes specifically expressed in one cell type were highly correlated when we analyzed all cell populations.

We examined the importance of HALLMARK_INTERFERON_GAMMA_RESPONSE in COVID-19 infection. In network analysis of B cells, interferon-induced antiviral factor (*IFITM3*) was the hub gene (Table 5). *IFITM3* inhibits SARS-CoV-2 infection by preventing SARS-CoV-2 spike-protein-mediated virus entry and cell-to-cell fusion. Analysis of a Chinese COVID-19 patient cohort demonstrated that the rs12252 C genotype of *IFITM3* is associated with the SARS-CoV-2 infection risk in the studied cohort. These data suggest that individuals carrying the rs12252 C allele in the *IFITM3* gene may be vulnerable to SARS-CoV-2 infection and benefit from early medical intervention [25].

The *IFITM3* rs6598045 G allele was significantly more common in deceased COVID-19 patients than in those who recovered. Highest mortality rates were observed in the Delta variant and with the lowest qPCR Ct values. COVID-19 mortality was associated with the *IFITM3* rs6598045 GG and AG in the Delta variant and the *IFITM3* rs6598045 AG in the Alpha variant. A statistically significant difference was observed in the qPCR Ct values between individuals with GG and AG genotypes and those with an AA genotype [26]. *IFITM* proteins are directly involved in adaptive immunity, and they regulate CD4+ T helper cell differentiation [27]. *IFITM3* also directly engages and shuttles incoming virus particles to lysosomes [28].

*IFITM3* was also a hub gene in the differential network of CD4^+^ T cells, ranking 12 out of 118 genes (Table 6). The number one hub gene was *BST2*, which was associated with COVID-19. There was a decrease in SARS-CoV-2 in cells with deleted transmembrane *BST2* domains compared to the initial Vero cell line. Similar results were obtained for SARS-CoV-2 and avian influenza virus [29]. Another study found that BST-2 restricts SARS-CoV-2 virion egress by tethering virions to the plasma membrane. We also identified several SARS-CoV-2 proteins that are putative modulators of *BST2* function [30]. *BST2* is an antiviral protein that inhibits the release and spread of many viruses and is upregulated as part of the innate immune defense against infections [31]. *BST2* can respond to infection by inducing proinflammatory responses via NF-κb signaling pathway activation [32].

Successful identification of hub genes illustrated the capability of GSNCASCR in prioritizing disease-related genes for understanding pathophysiology of disease and potential therapies.

DADA2 (deficiency of adenosine deaminase 2) is a vasculitis disease caused by autosomal-recessive loss-of-function mutations in the *ADA2* gene [33]. The spectrum of disease manifestations includes vasculitis, vasculopathy, and inflammation. ADA2 protein is primarily secreted by stimulated monocytes and macrophages, and aberrant monocyte differentiation to macrophages is important in the pathogenesis of DADA2. We also applied GSNCASCR to an scRNA-seq dataset comprising monocytes, CD4^+^, and CD8^+^ T lymphocytes of DADA2 patients and the results are shown in Table 7.

As expected, gene sets identified by GSNCASCR in monocytes in DADA2 patients were highly related with immune response, including IFN-γ and IFN-α and TNF-α signaling via NFκB and other pathways, indicating activation of monocytes and general inflammation in DADA2. Our previous research also revealed that T lymphocytes were activated and potentially contributed to exaggerated inflammation via ligand–receptor interactions with monocytes [34]. Consistently, upregulation of genes in the immune pathways such as *IFN-γ* and *IFN-α*, IL6 JAK STAT3 signaling, IL2 STAT5 signaling, and TNF-α signaling via NFκB were seen in CD4^+^ T cells of DATA2 patients, defined by GSNCASCR [33]. GSNCASCR also showed that CD8^+^ T cells in DADA2 upregulated stress pathways, including unfolded protein response, UV response, and inflammation (TNF-α signaling via NFκB and PI3K AKT MTOR signaling), suggesting T cell activation, cytotoxicity, and contribution to inflammation in the disease [34,35].

The results from GSNCA and GSCA applied to DADA2 and COVID-19 datasets are presented in Supplementary File S4. While most findings aligned with those from GSNCASCR, some discoveries were not clearly identified by these two tools. For instance, GSCA and GSNCA also identified immune response pathways to be differentially co-expressed in monocytes in DADA2, but GSNCA failed in CD4 and CD8 cells, and GSCA failed in CD8 cells. We recommend using multiple software tools on real datasets to thoroughly assess both consistent and inconsistent results for biological interpretation.

## 3. Discussion

We propose a statistical test, GSNCACR, to advantageously integrate GSNCA and CSCORE, and to better detect significant changes in the co-expression structure between two different biological conditions.

To further improve co-expression analysis for scRNA-seq data, one possibility is to use neighboring information of co-expression networks to refine gene–gene dependence identification. For example, topological overlap measure is a combination of the adjacency values between a pair of genes as well as the adjacency values these genes have with other genes to which they are connected [36].

Due to a high dropout rate, imputation can be considered in the future, and also batch correction, sequence depth, and other factors. Imputation with a sophisticated approach, such as Markov affinity-based graph imputation of cells, can denoise the cell count matrix and fill in missing transcripts, making it more effective in recovering gene–gene relationships [37]. Our program can run in parallel with multiple cores under Linux. However, on a personal computer, about 10 h is needed to calculate 100 pathways when using permutations to estimate statistical significance. The algorithm can be improved to increase computational speed. Additionally, our algorithm, including CS-CORE, supports parallel execution on Linux systems, which can enhance performance. Since individual pathways are treated independently, users can divide a pathway set into small pathway subsets, run the program on each subset separately, and then merge the results. The number of cells in the dataset impacts processing time; we have found that having around 3000 cells, with comparable numbers in both healthy and control groups, is optimal.

One limit of GSNCACR is its reliance on the quality and completeness of pathway databases. The quality and completeness of biological pathway content can vary significantly. Usually, large datasets such as GO have low quality. Users can choose different pathway datasets, depending on their study aim, for screening or validating. Recent studies have emphasized the contribution of cell–cell interactions across different cell populations in normal tissues and disease states [38]. Also, GSNCACR cannot examine the relationships of differentially co-expressed pathways across different cell populations, which would be another direction for improvement.

Integration with some known regulatory markers, such as K4me2, K4me3, K27ac, and ATAC-seq signals, can enhance co-expression estimation [39]. Additionally, integrating external datasets like STRING and BioGRID can also improve these estimations [40,41].

Interpreting and validating co-expression changes of gene pairs within interesting pathways is important. We plan to develop a Shiny app tool that allows users to interactively examine these changes in the context of STRING databases [39]. While it is important to validate co-expression changes with external databases for performance evaluation, there are currently no comprehensive databases detailing interaction changes due to diseases or biological processes.

There are many software packages for differential co-expression analysis at the gene pair, network, and subnetwork levels. Though useful, results are noisy and challenging to interpret. There are only several co-expression software packages based on well-defined pathways (functionally annotated gene set) [42,43]. Compared to network analysis, results from pathway analysis are more easily comprehensible for biologists to interpret and to infer a biological hypothesis.

## 4. Materials and Methods

The GSNCASCR R package compares gene co-expression networks in terms of their structural properties. In the following subsections, we explain the construction of co-expression networks (graphs), the graph spectral analysis, and the package’s main features.

### 4.1. Simulated and Read Datasets

We used scDesign2 as the simulator because we desired synthetic cells that preserved real genes and gene–gene correlations observed in real data [8], which preserved genes and gene–gene correlations and allowed us to generate non-zero-inflated data, making it easy for us to introduce non-biological zeros using various masking schemes. We focused on 500 genes randomly sampled from the top 5000 highly expressed genes with probabilities proportional to the inverse density of expression levels.

Real datasets were downloaded. We used the scRNA-seq data on PBMCs from COVID-19 patients and healthy donors from [19], at the NCBI Gene Expression Omnibus (accession no. GSE150728). The data are available at https://hosted-matrices-prod.s3-us-west-2.amazonaws.com/Single_cell_atlas_of_peripheral_immune_response_to_SARS_CoV_2_infection-25/blish_covid.seu.rds (accessed on 22 May 2023). The datasets contain the metadata of cell types. The subsets of B cells, CD8^+^ T cells, CD4^+^ T cells, and monocytes were extracted from the Seurat object. The datasets of DADA2 patients were downloaded from GEO (accession IDs, GSE142444 and GSE168163) [33,35].

The gene lists of Hallmark and GO biology process gene sets were downloaded from the Gene Set Enrichment Analysis (GSEA) database https://www.gsea-msigdb.org/gsea/msigdb (accessed on 20 October 2022).

### 4.2. Estimation of Co-Expression Gene Pairs

The first step is to estimate co-expression from scRNA-seq data with CS-CORE, which models unobserved true gene expression levels as latent variables, linked to observed UMI counts through a measurement model that accounts for both sequencing depth variations and measurement errors.

Under the expression measurement model of a Poisson distribution:

(1) $$\left(z_{i1},\ldots ,z_{ip}\right)\sim F_{p}\left(\mu ,\;\Sigma \right),\;x_{ij}|z_{ij}\sim Poisson\left(s_{i}z_{i1}\right)$$

Here, $x_{ij}$ is a UMI count of gene j in cell i, assumed to follow a Poisson measurement model depending on an underlying expression level $z_{ij}$ and sequencing depth $s_{i}$.

With $E\left(x_{ij}\right)=s_{i}\mu_{i},\;Var\left(x_{ij}\right)=s_{i}\mu_{i}+s_{i}^{2}\sigma_{jj}$ and $E\left[\left(x_{ij}-s_{i}\mu_{j}\right)\left(x_{ij^{\prime }}-s_{i}\mu_{j^{\prime }}\right)\right]=s_{i}\sigma_{jj^{\prime }}$, CS-Core estimates $\;\mu_{j}$ via the regression approaches. CS-CORE selects and updates weights via an IRLS procedure, such that the weighted least squares estimators are statistically efficient.

Next, CS-CORE develops a statistical test to assess whether a gene pair has independent expression levels. When $z_{ij}$ and $z_{ij^{\prime }}$ are independent, $Var\left(\xi_{ijj^{\prime }}\right)=\left(s_{i}\mu_{j^{\prime }}+s_{i}^{2}\sigma_{jj}\right)\left(s_{i}\mu_{j^{\prime }}+s_{i}^{2}\sigma_{jj^{\prime }}\right)=1/g_{ijj^{\prime }}$. Letting ${\hat{\sigma }}_{jj^{\prime }}$ be estimated with true $\mu_{j}$s, the test statistic is defined as $T_{jj^{\prime }}={\hat{\sigma }}_{jj^{\prime }}/\sqrt{Var\left({\hat{\sigma }}_{jj^{\prime }}\right)}$.

It follows that $T_{jj^{\prime }}\sim N\left(0,1\right)$ under the null hypothesis that $z_{ij}$ and $z_{ij^{\prime }}$ are independent. This result allows us to directly compute p-values by plugging in IRLS estimated $u_{j^{\prime }}^{\prime }$ and ${\sigma_{jj}}^{\prime }$ values, all of which are consistent to weight least squares estimators.

### 4.3. Identification of Co-Expressed Pathways

The GSNCA method detects differences in a network correlation structure for a gene set between two conditions [4] and is implemented in function GSNCAtest. Genes under each phenotype are assigned weight factors that are adjusted simultaneously such that equality is achieved between each gene’s weight as well as a sum of its weighted correlations with other genes in a gene set of *p* genes:

(2) $$w_{i}=\sum_{j\neq i}w_{j}r_{ij},\;1\leq i\leq p$$

where $r_{ij}$ is the correlation estimated by CS-CORE, and then solves as an eigenvector problem with a unique solution that is an eigenvector corresponding to the largest eigenvalue of the genes’ correlation matrix.

As a test statistic, $w_{GSNCASCR}$, we use the L1 norm between the scaled weight vectors w(1) and w(2) (each vector is multiplied by its norm to scale weight factor values around one) between two conditions. The test statistic GSNCASCR is the first norm between two scaled weight vectors under two phenotypes where each vector is multiplied by its norm.

(3) $$w_{GSCNASCR}=\sum_{i=1}^{p}\left|w_{i,norm}^{\left(1\right)}-w_{i,\;norm}^{\left(2\right)}\right|$$

We use this test statistic to test the hypothesis H0: $w_{GSNCASCR}=0$ against the alternative H1: $w_{GSNCASCR}\neq 0$. We downloaded the code of the GSAR package, which implemented the GSNCAtest function and used it in our package. In this function, GSNCAtest uses permutations to estimate *p*-values (https://bioconductor.org/packages/release/bioc/manuals/GSAR/man/GSAR.pdf (accessed on 12 May 2023)). The *p*-values for the test statistic are obtained by comparing the observed value of the test statistic to its null distribution, which is estimated using a permutation approach.

### 4.4. Identification of Hub Genes in Pathways

Hub genes provide useful biological information beyond the result that a pathway is differentially co-expressed between two conditions. A weighted node connectivity (WNC) score can be specified as follows:

(4) $$WNC_{i}=\sum_{j}^{N}w_{ij}$$

where node i is connected to node j, and $w_{ij}$ reflects the strength of a connection of node i with node j. In this paper, $w_{ij}$ is computed as an absolute value of a correlation (differential correlation in differential networks) between genes i and j estimated by GSNCASCR.

## 5. Conclusions

GSNCASCR identified differential gene sets through examining co-expression networks with scRNA-seq data. It performs better than GSCNA and GSCA, with higher precision and accuracy. As an additional result from GSNCASCR, we defined hub genes as genes with the largest weights and showed that these genes corresponded frequently to major and specific pathway regulators, as well as to genes that were most affected by the biological difference between two conditions. GSNCASCR is a new approach, resulting in the generation of novel biological hypotheses at both gene and pathway levels. This package provides pathways for understanding the mechanism of diseases and hub genes for functional studies.

In addition to Supplementary File S1, a vignette for analysis of CD4 cells in COVID-19 (available in Appendix A), several additional vignettes are available on our GitHub repository. These resources provide comprehensive guidance for users to effectively utilize the analytical tools. Furthermore, they should be useful in allowing other users to reproduce our analysis and to track the tool’s analysis procedures. These vignettes also provide exemplary steps for others to establish pipelines for their own single-cell data.

## Figures and Tables

**Table 1:** Comparison of average true positive rates (sensitivities), false positive rates, precision, and accuracy of the three tools.

Method	Sensitivity	False Positive Rate	Precision	Accuracy
GSNCASCR	0.69	0.00	1.00	0.79
GSNCA	0.60	0.15	0.77	0.73
GSCA	1.00	0.59	0.60	0.69

Sensitivity = TP/(TP + FN), False positive rate = FP/(FP + TN), Precision = TP/(TP + FP), and Accuracy = (TP + TN)/(TP + TN + FP + FN). TP, true positive; FN, false negative; FP, false positive; TN, true negative.

**Table 2:** Gene sets identified by GSCNASCR in the CD4^+^ T cells.

Pathway	*p*-Value
HALLMARK_INTERFERON_GAMMA_RESPONSE	1.42 × 10^−21^
HALLMARK_INTERFERON_ALPHA_RESPONSE	2.34 × 10^−20^
HALLMARK_KRAS_SIGNALING_DN	1.09 × 10^−13^
HALLMARK_TNFA_SIGNALING_VIA_NFKB	9.53 × 10^−13^
HALLMARK_COMPLEMENT	3.04 × 10^−9^
HALLMARK_FATTY_ACID_METABOLISM	5.25 × 10^−9^
HALLMARK_XENOBIOTIC_METABOLISM	9.73 × 10^−9^
HALLMARK_ALLOGRAFT_REJECTION	7.31 × 10^−8^
HALLMARK_IL2_STAT5_SIGNALING	7.61 × 10^−8^
HALLMARK_IL6_JAK_STAT3_SIGNALING	1.11 × 10^−7^
HALLMARK_DNA_REPAIR	3.10 × 10^−6^
HALLMARK_TGF_BETA_SIGNALING	3.52 × 10^−6^
HALLMARK_OXIDATIVE_PHOSPHORYLATION	5.27 × 10^−5^
HALLMARK_ADIPOGENESIS	1.26 × 10^−5^
HALLMARK_HYPOXIA	1.52 × 10^−5^
HALLMARK_APICAL_JUNCTION	1.95 × 10^−5^
HALLMARK_KRAS_SIGNALING_UP	2.17 × 10^−5^
HALLMARK_REACTIVE_OXYGEN_SPECIES_PATHWAY	6.85 × 10^−5^
HALLMARK_COAGULATION	7.04 × 10^−5^
HALLMARK_APICAL_SURFACE	0.000129693

**Table 3:** Gene sets identified by GSNCASCR in the B cells.

Pathway	*p*-Value
HALLMARK_ALLOGRAFT_REJECTION	1.89 × 10^−28^
HALLMARK_IL2_STAT5_SIGNALING	8.11 × 10^−21^
HALLMARK_INTERFERON_GAMMA_RESPONSE	4.67 × 10^−20^
HALLMARK_TNFA_SIGNALING_VIA_NFKB	4.35 × 10^−19^
HALLMARK_KRAS_SIGNALING_UP	1.36 × 10^−16^
HALLMARK_ESTROGEN_RESPONSE_EARLY	1.60 × 10^−16^
HALLMARK_P53_PATHWAY	6.02 × 10^−16^
HALLMARK_HYPOXIA	2.22 × 10^−15^
HALLMARK_UV_RESPONSE_DN	1.71 × 10^−14^
HALLMARK_MYC_TARGETS_V2	1.97 × 10^−14^
HALLMARK_E2F_TARGETS	3.27 × 10^−14^
HALLMARK_G2M_CHECKPOINT	1.66 × 10^−13^
HALLMARK_MTORC1_SIGNALING	4.99 × 10^−13^
HALLMARK_APOPTOSIS	7.16 × 10^−13^
HALLMARK_PROTEIN_SECRETION	1.01 × 10^−12^
HALLMARK_ESTROGEN_RESPONSE_LATE	1.56 × 10^−12^
HALLMARK_CHOLESTEROL_HOMEOSTASIS	1.83 × 10^−12^
HALLMARK_MYC_TARGETS_V1	2.07 × 10^−12^
HALLMARK_COMPLEMENT	3.43 × 10^−12^
HALLMARK_PANCREAS_BETA_CELLS	7.39 × 10^−12^

**Table 4:** Gene sets identified by GSNCASCR in the CD8^+^ T cells.

Pathway	*p*-Value
HALLMARK_HYPOXIA	3.71 × 10^−22^
HALLMARK_G2M_CHECKPOINT	6.01 × 10^−22^
HALLMARK_MYC_TARGETS_V1	1.53 × 10^−20^
HALLMARK_INTERFERON_ALPHA_RESPONSE	5.19 × 10^−19^
HALLMARK_E2F_TARGETS	7.08 × 10^−19^
HALLMARK_ALLOGRAFT_REJECTION	2.50 × 10^−18^
HALLMARK_MTORC1_SIGNALING	9.14 × 10^−17^
HALLMARK_APICAL_JUNCTION	1.66 × 10^−15^
HALLMARK_UV_RESPONSE_UP	5.53 × 10^−15^
HALLMARK_INTERFERON_GAMMA_RESPONSE	9.81 × 10^−15^
HALLMARK_UNFOLDED_PROTEIN_RESPONSE	1.00 × 10^−14^
HALLMARK_MITOTIC_SPINDLE	1.32 × 10^−14^
HALLMARK_IL2_STAT5_SIGNALING	1.01 × 10^−13^
HALLMARK_TNFA_SIGNALING_VIA_NFKB	1.18 × 10^−12^
HALLMARK_XENOBIOTIC_METABOLISM	5.88 × 10^−12^
HALLMARK_UV_RESPONSE_DN	6.04 × 10^−11^
HALLMARK_GLYCOLYSIS	3.25 × 10^−10^
HALLMARK_MYC_TARGETS_V2	4.39 × 10^−10^
HALLMARK_PI3K_AKT_MTOR_SIGNALING	5.17 × 10^−5^
HALLMARK_FATTY_ACID_METABOLISM	1.40 × 10^−9^

**Table 5:** Top hub genes in the network of the IFN-γ pathway identified in B cells.

Gene	Degree in COVID-19	Degree in Healthy	Degree in Difference
*PSMB2*	39.62	25.02	39.21
*IFITM3*	24.22	44.54	38.32
*IFI35*	42.9	21.91	33.26
*PTPN2*	34.64	14.37	32.82
*IRF5*	29.9	16.22	26.54
*CD40*	29.8	12.29	24.19
*MTHFD2*	32.95	11.26	23.06
*CASP4*	22.08	22.68	22.74
*BST2*	28.95	14.26	22.62
*IFNAR2*	27.08	12.81	22.01
*HLA-G*	25.65	21.32	22
*LY6E*	27.68	20.66	21.7
*LAP3*	28.44	26.56	21.63
*HLA-DMA*	29.15	15.12	21.5
*OGFR*	24.44	15.61	21.28
*STAT2*	29.45	13.3	20.76
*CD38*	34.48	15.12	20.58
*HLA-DRB1*	29.29	12.27	20.56
*PSMB2*	39.62	25.02	39.21
*IFITM3*	24.22	44.54	38.32

**Table 6:** Top hub genes in the network of the IFN-γ pathway identified in CD4^+^ T cells.

Gene	Degree in COVID-19	Degree in Healthy	Degree in Difference
*BST2*	26.16	14.76	22.08
*SRI*	21.88	17.74	21.67
*OGFR*	21.02	11.82	18.87
*LAP3*	24.6	12.19	18.62
*LY6E*	26.82	22.24	16.68
*NMI*	21.58	15.11	16.66
*MYD88*	26.17	15.46	16.54
*HLA-G*	18.63	17.57	16.29
*IFI44L*	26.89	9.7	15.99
*RSAD2*	25.8	9.23	15.67
*MX2*	23.83	9.99	15.32
*IFITM3*	19.33	14.97	15.12
*CASP4*	20.56	13.68	14.83
*OAS3*	28.31	13.93	14.81
*PARP14*	22.44	10.35	14.56
*OAS2*	32.82	17.39	14.18
*IFIT1*	26.56	13.92	13.95
*STAT2*	23.22	16.76	13.92
*UBE2L6*	25.05	18.35	13.38
*RAPGEF6*	16.65	15.45	13.37

**Table 7:** Gene sets identified by GSNCASCR in the monocytes in DADA2.

Type	Pathway	*p*-Value
Monocyte	HALLMARK_INTERFERON_GAMMA_RESPONSE	1.19 × 10^−22^
Monocyte	HALLMARK_INTERFERON_ALPHA_RESPONSE	2.73 × 10^−17^
Monocyte	HALLMARK_INFLAMMATORY_RESPONSE	5.61 × 10^−14^
Monocyte	HALLMARK_ALLOGRAFT_REJECTION	1.87 × 10^−12^
Monocyte	HALLMARK_ADIPOGENESIS	1.87 × 10^−11^
Monocyte	HALLMARK_TNFA_SIGNALING_VIA_NFKB	1.51 × 10^−9^
Monocyte	HALLMARK_ESTROGEN_RESPONSE_LATE	2.43 × 10^−9^
Monocyte	HALLMARK_PROTEIN_SECRETION	3.09 × 10^−9^
Monocyte	HALLMARK_NOTCH_SIGNALING	3.30 × 10^−9^
Monocyte	HALLMARK_XENOBIOTIC_METABOLISM	1.83 × 10^−8^
CD4^+^ T	HALLMARK_INTERFERON_GAMMA_RESPONSE	3.47 × 10^−15^
CD4^+^ T	HALLMARK_IL6_JAK_STAT3_SIGNALING	4.58 × 10^−15^
CD4^+^ T	HALLMARK_INTERFERON_ALPHA_RESPONSE	4.86 × 10^−14^
CD4^+^ T	HALLMARK_IL2_STAT5_SIGNALING	1.42 × 10^−13^
CD4^+^ T	HALLMARK_TNFA_SIGNALING_VIA_NFKB	4.46× 10^−13^
CD4^+^ T	HALLMARK_ALLOGRAFT_REJECTION	6.27 × 10^−12^
CD4^+^ T	HALLMARK_KRAS_SIGNALING_UP	6.78 × 10^−12^
CD4^+^ T	HALLMARK_APOPTOSIS	1.75 × 10^−11^
CD4^+^ T	HALLMARK_EPITHELIAL_MESENCHYMAL_TRANSITION	1.82 × 10^−1^
CD4^+^ T	HALLMARK_OXIDATIVE_PHOSPHORYLATION	7.87 × 10^−9^
CD8^+^ T	HALLMARK_MYC_TARGETS_V1	4.06 × 10^−13^
CD8^+^ T	HALLMARK_TNFA_SIGNALING_VIA_NFKB	1.85 × 10^−11^
CD8^+^ T	HALLMARK_COMPLEMENT	5.99 × 10^−11^
CD8^+^ T	HALLMARK_UNFOLDED_PROTEIN_RESPONSE	2.04 × 10^−10^
CD8^+^ T	HALLMARK_PANCREAS_BETA_CELLS	1.95 × 10^−9^
CD8^+^ T	HALLMARK_UV_RESPONSE_UP	4.93 × 10^−9^
CD8^+^ T	HALLMARK_INFLAMMATORY_RESPONSE	1.05 × 10^−8^
CD8^+^ T	HALLMARK_CHOLESTEROL_HOMEOSTASIS	3.01 × 10^−8^
CD8^+^ T	HALLMARK_PI3K_AKT_MTOR_SIGNALING	4.83 × 10^−7^
CD8^+^ T	HALLMARK_ESTROGEN_RESPONSE_LATE	1.69 × 10^−6^

## Data Availability

Processed data are freely downloaded from the covid19cellatlas.org website.

## Supplementary Materials

The following supporting information can be downloaded at: https://www.mdpi.com/article/10.3390/ijms26104771/s1.

## Appendix A

Vignette of analysis of CD4^+^ T cells of COVID-19 patients is available at https://htmlpreview.github.io/?https://github.com/shouguog/GSNCASCR/blob/main/vignette/COVIDCD4Tcell.html (accessed on 22 June 2023), and a pdf file is attached as Supplementary Materials.
