# Supplementary material for: GSNCASCR: An R Package to Identify Differentially Co-Expressed Curated Gene Sets with Single-Cell RNA-Seq Data
Source: Int J Mol Sci. 2025 May 16;26(10):4771. doi: 10.3390/ijms26104771 (PMC12112291; doi:10.3390/ijms26104771)
Supplement: Supplementary file 1 [file ijms-26-04771-s001.zip › File S1-Vignette_CD4T.pdf]

Vignette Title

***Shouguo Gao*[1] *and Neal S. Young*[1*]**

[1]NHLBI/NIH

[*]shouguo.gao@nih.gov (mailto:shouguo.gao@nih.gov)

26 November 2023

Abstract

Description of your vignette

Contents

1 1. Load packages and data

2 2. Select cell types and gene sets to study

3 3. Run `Pathway Level` to infer cell-type-specific co-expression network on the specified gene set

4 4. Get the Subset of two groups

5 5. Calculate the network, or score of networks

6 6. Calculate the network, or score of networks for permutations

7 7. Calculate p values

8 8. Generate the figures for top pathways

9 9. Show plots

10 10. Show plots

11 11. Show plots

12 12. Show plots

This vignette shows an example of applying pathway level to infer cell-type-specific co-expression networks and extracting co-expressed gene modules that are enriched for biological functions in cell types.

1 1. Load packages and data

```
library(CSCORE)
library(Seurat)
library(ggraph)
library(gridExtra)
source("../R/functions_2.R")
```

In this vignette, we use the single cell RNA-sequencing data on Peripheral blood mononuclear cells (PBMC) from COVID patients and healthy controls from Wilk et al. (https://www.nature.com/articles/s41591-020-0944-y), which were also studied in our manuscript (https://www.biorxiv.org/content/10.1101/2022.12.13.520181v1). This data set can be downloaded via the following bash script

```
#wget https://hosted-matrices-prod.s3-us-west-2.amazonaws.com/Single_
cat("dowload")
```

```
## dowload
```

After downloading blish_covid.seu.rds, we load it into the R session

```
####Load the gene set and convert into list
geneset<-geneIds(getGmt("../data/h.all.v2023.2.Hs.symbols.gmt"))
pbmc <- readRDS('../data/blish_covid.seu.rds')
# Use the original UMI counts stored in Assay 'RNA'
DefaultAssay(pbmc) <- 'RNA'
```

2 2. Select cell types and gene sets to study

In this example, we focus on CD4T cells and infer the CD4T cell-specific co-expression network.

```
pbmc_CD4T = pbmc[,pbmc$cell.type.coarse %in% 'CD4 T']
```

Depending on the biological question of interest, one may choose to study the co-expression network for any gene set. Here, we chose to infer the co-expression network for the genes with meaningful expression levels in CD4T cells (top 5000 among 26361 genes). There are several reasons for our choice:

1. All genes with moderate to high expression levels provides a comprehensive and unbiased set of genes that could have meaningful biological functions in a cell type.
2. If the genes have much lower expression levels, it would be statistically more challenging and biologically less interesting to infer their co-expressions, as these genes might have almost all UMI counts equal to 0.

In general, it will be up to the users's choice to select the gene sets to study. We recommend choosing the gene sets that are of interest to your application.

```
mean_exp = rowMeans(pbmc_CD4T@assays$RNA@counts/pbmc_CD4T$nCount_RNA)
genes_selected = names(sort.int(mean_exp, decreasing = T))[1:5000]
```

3 3. Run `Pathway Level` to infer cell-type-specific co-expression network on the specified gene set

We further subset the CD4 T cells to those from healthy control subjects in order to study CD4T-cell specific co-expression network among healthy control CD4T cells.

```
metaData<-pbmc_CD4T@meta.data
metaData_Healthy<-metaData[metaData$Status=="Healthy",]
metaData_COVID<-metaData[metaData$Status=="COVID",]
set.seed(666);metaData_COVID<-metaData_COVID[sample(1:dim(metaData_CO
```

4 4. Get the Subset of two groups

```
pbmc_CD4T.sub<-subset(pbmc_CD4T, cells=c(rownames(metaData_Healthy),
rm(pbmc_CD4T)
```

5 5. Calculate the network, or score of networks

```
cor_data_score<-calculate_seq_correlation(geneset,pbmc_CD4T.sub,genes
```

```
## [1] "IRLS converged after 5 iterations."
## [1] "13 among 5000 genes have negative variance estimates. Their c
## [1] "0.1293% co-expression estimates were greater than 1 and were
## [1] "0.0751% co-expression estimates were smaller than -1 and were
## [1] "IRLS converged after 3 iterations."
## [1] "8 among 5000 genes have negative variance estimates. Their co
## [1] "0.0288% co-expression estimates were greater than 1 and were
## [1] "0.0144% co-expression estimates were smaller than -1 and were
## I am here line 62
```

```
save(cor_data_score, file = "CD4T_COVIDHD_cor_data_score.RData")
```

Next, based on the thresholded co-expression matrix, we apply WGCNA (https://bmcbioinformatics.biomedcentral.com/articles/10.1186/1471-2105-9-559) to extract co-expressed gene modules. In particular, we use `CS-CORE` estimates to measure co-expressions for single cell RNA-sequencing data, which replace the Pearson correlations used in traditional WGNCA workflow, that suffer from inflated false positives and attenuation bias on single cell data as demonstrated in our manuscript (https://www.biorxiv.org/content/10.1101/2022.12.13.520181v1).

6 6. Calculate the network, or score of networks for permutations

```
cor_data_perm_list<-runPermute_seq_correlation(geneset,pbmc_CD4T.sub,
```

```
## ###########################################1701031416   1   perm
## covid_557.3526 HIP044.1993 covid_557.5882 HIP044.3144 covid_557.64
## [1] "5 among 5000 genes have negative variance estimates. Their co
## [1] "0.0850% co-expression estimates were greater than 1 and were
## [1] "0.0574% co-expression estimates were smaller than -1 and were
## [1] "IRLS converged after 4 iterations."
## [1] "4 among 5000 genes have negative variance estimates. Their co
## [1] "0.0379% co-expression estimates were greater than 1 and were
## [1] "0.0189% co-expression estimates were smaller than -1 and were
## ###########################################1701031930   2   perm
## HIP023.3203 HIP044.3368 HIP044.2118 covid_557.4071 HIP044.605covic
## [1] "4 among 5000 genes have negative variance estimates. Their co
## [1] "0.0587% co-expression estimates were greater than 1 and were
## [1] "0.0342% co-expression estimates were smaller than -1 and were
## [1] "IRLS converged after 3 iterations."
## [1] "3 among 5000 genes have negative variance estimates. Their co
## [1] "0.0544% co-expression estimates were greater than 1 and were
## [1] "0.0285% co-expression estimates were smaller than -1 and were
## ###########################################1701032464   3   perm
## covid_557.5972 HIP023.2625 covid_556.1198 HIP044.3500 HIP002.684HI
## [1] "3 among 5000 genes have negative variance estimates. Their co
## [1] "0.0540% co-expression estimates were greater than 1 and were
## [1] "0.0250% co-expression estimates were smaller than -1 and were
## [1] "IRLS converged after 4 iterations."
## [1] "7 among 5000 genes have negative variance estimates. Their co
## [1] "0.0485% co-expression estimates were greater than 1 and were
## [1] "0.0243% co-expression estimates were smaller than -1 and were
## ###########################################1701032993   4   perm
## HIP045.2174 covid_557.5234 HIP002.340 covid_555_2.2849 HIP043.2736
## [1] "8 among 5000 genes have negative variance estimates. Their co
## [1] "0.0565% co-expression estimates were greater than 1 and were
## [1] "0.0269% co-expression estimates were smaller than -1 and were
## [1] "IRLS converged after 3 iterations."
## [1] "3 among 5000 genes have negative variance estimates. Their co
## [1] "0.0373% co-expression estimates were greater than 1 and were
## [1] "0.0126% co-expression estimates were smaller than -1 and were
## ###########################################1701033544   5   perm
## HIP044.2700 HIP043.3 covid_557.5146 covid_555_2.688 HIP044.2937cov
## [1] "4 among 5000 genes have negative variance estimates. Their co
## [1] "0.0422% co-expression estimates were greater than 1 and were
## [1] "0.0203% co-expression estimates were smaller than -1 and were
## [1] "IRLS converged after 3 iterations."
## [1] "5 among 5000 genes have negative variance estimates. Their co
## [1] "0.0384% co-expression estimates were greater than 1 and were
## [1] "0.0132% co-expression estimates were smaller than -1 and were
## ###########################################1701034066   6   perm
## HIP043.2149 covid_559.324 covid_555_1.2211 HIP023.2617 HIP045.1250
## [1] "6 among 5000 genes have negative variance estimates. Their co
## [1] "0.0547% co-expression estimates were greater than 1 and were
## [1] "0.0269% co-expression estimates were smaller than -1 and were
## [1] "IRLS converged after 4 iterations."
## [1] "4 among 5000 genes have negative variance estimates. Their co
## [1] "0.0629% co-expression estimates were greater than 1 and were
## [1] "0.0431% co-expression estimates were smaller than -1 and were
## ###########################################1701034586   7   perm
## covid_557.1488 HIP044.926 covid_555_1.2612 covid_557.5405 HIP045.1
## [1] "3 among 5000 genes have negative variance estimates. Their co
```

```
## [1] "0.0297% co-expression estimates were greater than 1 and were
## [1] "0.0122% co-expression estimates were smaller than -1 and were
## [1] "IRLS converged after 3 iterations."
## [1] "5 among 5000 genes have negative variance estimates. Their co
## [1] "0.0402% co-expression estimates were greater than 1 and were
## [1] "0.0167% co-expression estimates were smaller than -1 and were
## ############################################1701035112   8   perm
## HIP045.1230 HIP023.89 covid_557.4565 HIP044.1572 covid_557.2927cov
## [1] "7 among 5000 genes have negative variance estimates. Their co
## [1] "0.0836% co-expression estimates were greater than 1 and were
## [1] "0.0504% co-expression estimates were smaller than -1 and were
## [1] "IRLS converged after 3 iterations."
## [1] "4 among 5000 genes have negative variance estimates. Their co
## [1] "0.0393% co-expression estimates were greater than 1 and were
## [1] "0.0237% co-expression estimates were smaller than -1 and were
## ############################################1701035627   9   perm
## covid_558.197 HIP045.1007 HIP044.560 covid_556.1404 covid_560.119
## [1] "2 among 5000 genes have negative variance estimates. Their co
## [1] "0.0505% co-expression estimates were greater than 1 and were
## [1] "0.0298% co-expression estimates were smaller than -1 and were
## [1] "IRLS converged after 4 iterations."
## [1] "5 among 5000 genes have negative variance estimates. Their co
## [1] "0.0480% co-expression estimates were greater than 1 and were
## [1] "0.0277% co-expression estimates were smaller than -1 and were
## ############################################1701036145   10  perm
## covid_557.401 HIP023.2421 HIP023.1014 HIP044.367 HIP045.1963HIP04
## [1] "8 among 5000 genes have negative variance estimates. Their co
## [1] "0.0282% co-expression estimates were greater than 1 and were
## [1] "0.0115% co-expression estimates were smaller than -1 and were
## [1] "IRLS converged after 3 iterations."
## [1] "4 among 5000 genes have negative variance estimates. Their co
## [1] "0.0426% co-expression estimates were greater than 1 and were
## [1] "0.0226% co-expression estimates were smaller than -1 and were
## ############################################1701036665   11  perm
## covid_555_1.2696 covid_556.872 covid_557.4745 covid_559.1998 HIP04
## [1] "5 among 5000 genes have negative variance estimates. Their co
## [1] "0.0499% co-expression estimates were greater than 1 and were
## [1] "0.0243% co-expression estimates were smaller than -1 and were
## [1] "IRLS converged after 3 iterations."
## [1] "6 among 5000 genes have negative variance estimates. Their co
## [1] "0.0499% co-expression estimates were greater than 1 and were
## [1] "0.0225% co-expression estimates were smaller than -1 and were
## ############################################1701037174   12  perm
## HIP044.805 HIP044.232 HIP044.180 covid_559.584 HIP002.607HIP043.1
## [1] "4 among 5000 genes have negative variance estimates. Their co
## [1] "0.0417% co-expression estimates were greater than 1 and were
## [1] "0.0180% co-expression estimates were smaller than -1 and were
## [1] "IRLS converged after 4 iterations."
## [1] "6 among 5000 genes have negative variance estimates. Their co
## [1] "0.0423% co-expression estimates were greater than 1 and were
## [1] "0.0194% co-expression estimates were smaller than -1 and were
## ############################################1701037693   13  perm
## HIP045.2029 covid_560.1647 covid_557.5090 HIP044.2420 covid_555_2.
## [1] "3 among 5000 genes have negative variance estimates. Their co
## [1] "0.0697% co-expression estimates were greater than 1 and were
## [1] "0.0450% co-expression estimates were smaller than -1 and were
## [1] "IRLS converged after 3 iterations."
```

```
## [1] "4 among 5000 genes have negative variance estimates. Their cc
## [1] "0.0723% co-expression estimates were greater than 1 and were
## [1] "0.0460% co-expression estimates were smaller than -1 and were
## ############################################1701038230   14  perm
## covid_559.955 covid_559.1443 HIP045.1039 HIP023.1261 covid_558.10C
## [1] "7 among 5000 genes have negative variance estimates. Their cc
## [1] "0.0489% co-expression estimates were greater than 1 and were
## [1] "0.0236% co-expression estimates were smaller than -1 and were
## [1] "IRLS converged after 3 iterations."
## [1] "2 among 5000 genes have negative variance estimates. Their cc
## [1] "0.0314% co-expression estimates were greater than 1 and were
## [1] "0.0130% co-expression estimates were smaller than -1 and were
## ############################################1701038777   15  perm
## HIP044.1028 covid_558.365 HIP043.3754 covid_559.1735 HIP043.1777HI
## [1] "4 among 5000 genes have negative variance estimates. Their cc
## [1] "0.0707% co-expression estimates were greater than 1 and were
## [1] "0.0430% co-expression estimates were smaller than -1 and were
## [1] "IRLS converged after 3 iterations."
## [1] "4 among 5000 genes have negative variance estimates. Their cc
## [1] "0.0316% co-expression estimates were greater than 1 and were
## [1] "0.0097% co-expression estimates were smaller than -1 and were
## ############################################1701039331   16  perm
## HIP023.2762 HIP044.2683 HIP043.943 HIP044.2429 HIP045.1071HIP044.2
## [1] "5 among 5000 genes have negative variance estimates. Their cc
## [1] "0.0351% co-expression estimates were greater than 1 and were
## [1] "0.0126% co-expression estimates were smaller than -1 and were
## [1] "IRLS converged after 3 iterations."
## [1] "2 among 5000 genes have negative variance estimates. Their cc
## [1] "0.0500% co-expression estimates were greater than 1 and were
## [1] "0.0259% co-expression estimates were smaller than -1 and were
## ############################################1701039846   17  perm
## covid_557.5424 covid_561.261 covid_560.1789 covid_557.2777 HIP045.
## [1] "2 among 5000 genes have negative variance estimates. Their cc
## [1] "0.0393% co-expression estimates were greater than 1 and were
## [1] "0.0154% co-expression estimates were smaller than -1 and were
## [1] "IRLS converged after 3 iterations."
## [1] "5 among 5000 genes have negative variance estimates. Their cc
## [1] "0.0428% co-expression estimates were greater than 1 and were
## [1] "0.0182% co-expression estimates were smaller than -1 and were
## ############################################1701040359   18  perm
## HIP044.2000 HIP045.1998 HIP044.3875 HIP023.1606 HIP044.2611covid_5
## [1] "5 among 5000 genes have negative variance estimates. Their cc
## [1] "0.0512% co-expression estimates were greater than 1 and were
## [1] "0.0223% co-expression estimates were smaller than -1 and were
## [1] "IRLS converged after 4 iterations."
## [1] "3 among 5000 genes have negative variance estimates. Their cc
## [1] "0.0539% co-expression estimates were greater than 1 and were
## [1] "0.0272% co-expression estimates were smaller than -1 and were
## ############################################1701040862   19  perm
## covid_557.548 HIP044.468 covid_557.5146 HIP044.2087 covid_556.14HI
## [1] "8 among 5000 genes have negative variance estimates. Their cc
## [1] "0.0255% co-expression estimates were greater than 1 and were
## [1] "0.0081% co-expression estimates were smaller than -1 and were
## [1] "IRLS converged after 3 iterations."
## [1] "4 among 5000 genes have negative variance estimates. Their cc
## [1] "0.0451% co-expression estimates were greater than 1 and were
## [1] "0.0194% co-expression estimates were smaller than -1 and were
```

```
## ############################################1701041358   20  perm
## covid_558.1219 covid_557.3245 covid_557.5651 covid_557.4129 HIP044
## [1] "5 among 5000 genes have negative variance estimates. Their cc
## [1] "0.0355% co-expression estimates were greater than 1 and were
## [1] "0.0191% co-expression estimates were smaller than -1 and were
## [1] "IRLS converged after 3 iterations."
## [1] "8 among 5000 genes have negative variance estimates. Their cc
## [1] "0.0329% co-expression estimates were greater than 1 and were
## [1] "0.0155% co-expression estimates were smaller than -1 and were
```

```
save(cor_data_perm_list, file = "CD4T_COVIDHD_cor_data_hallmark_perm_
```

7 7. Calculate p values

```
pvalues<-sort(getPvaluesTTest(cor_data_score, cor_data_perm_list))
save(pvalues, file = "CD4T_pvaluesTtest.RData")
```

8 8. Generate the figures for top pathways

```
gglist <-list()
for(ii in 1:1){
  pathway<-names(pvalues)[ii]
  networkslist<-network_seq_correlation(geneset[[pathway]],pbmc_CD4T.
  gglist[[ii]]<-network_plot(networkslist,name1="COVID",name2="Health
}
```

```
## [1] "IRLS converged after 5 iterations."
## [1] "13 among 5000 genes have negative variance estimates. Their c
## [1] "0.1293% co-expression estimates were greater than 1 and were
## [1] "0.0751% co-expression estimates were smaller than -1 and were
## [1] "IRLS converged after 3 iterations."
## [1] "8 among 5000 genes have negative variance estimates. Their cc
## [1] "0.0288% co-expression estimates were greater than 1 and were
## [1] "0.0144% co-expression estimates were smaller than -1 and were
```

```
## Warning: package 'dplyr' was built under R version 4.1.3
```

```
##
## Attaching package: 'dplyr'
```

```
## The following objects are masked from 'package:GSEABase':
##
##     intersect, setdiff, union
```

```
## The following object is masked from 'package:graph':
##
##     union
```

```
## The following object is masked from 'package:AnnotationDbi':
##
##     select
```

```
## The following objects are masked from 'package:IRanges':
##
##     collapse, desc, intersect, setdiff, slice, union
```

```
## The following objects are masked from 'package:S4Vectors':
##
##     first, intersect, rename, setdiff, setequal, union

## The following object is masked from 'package:Biobase':
##
##     combine

## The following objects are masked from 'package:BiocGenerics':
##
##     combine, intersect, setdiff, union

## The following object is masked from 'package:gridExtra':
##
##     combine

## The following objects are masked from 'package:stats':
##
##     filter, lag

## The following objects are masked from 'package:base':
##
##     intersect, setdiff, setequal, union

## Warning: package 'igraph' was built under R version 4.1.3

##
## Attaching package: 'igraph'

## The following objects are masked from 'package:dplyr':
##
##     as_data_frame, groups, union

## The following object is masked from 'package:GSEABase':
##
##     union

## The following objects are masked from 'package:graph':
##
##     degree, edges, intersection, union

## The following object is masked from 'package:IRanges':
##
##     union

## The following object is masked from 'package:S4Vectors':
##
##     union

## The following objects are masked from 'package:BiocGenerics':
##
##     normalize, path, union

## The following objects are masked from 'package:stats':
##
##     decompose, spectrum

## The following object is masked from 'package:base':
##
##     union

## Using "stress" as default layout
## Using "stress" as default layout
## Using "stress" as default layout
```

```
## Warning: Using the `size` aesthetic in this geom was deprecated ir
## i Please use `linewidth` in the `default_aes` field and elsewhere
## This warning is displayed once every 8 hours.
## Call `lifecycle::last_lifecycle_warnings()` to see where this warr
## generated.
```

```
for(ii in 2:2){
  pathway<-names(pvalues)[ii]
  networkslist<-network_seq_correlation(geneset[[pathway]],pbmc_CD4T.
  gglist[[ii]]<-network_plot(networkslist,name1="COVID",name2="Healtl
}
```

```
## [1] "IRLS converged after 5 iterations."
## [1] "13 among 5000 genes have negative variance estimates. Their c
## [1] "0.1293% co-expression estimates were greater than 1 and were
## [1] "0.0751% co-expression estimates were smaller than -1 and were
## [1] "IRLS converged after 3 iterations."
## [1] "8 among 5000 genes have negative variance estimates. Their cc
## [1] "0.0288% co-expression estimates were greater than 1 and were
## [1] "0.0144% co-expression estimates were smaller than -1 and were
```

```
## Using "stress" as default layout
## Using "stress" as default layout
## Using "stress" as default layout
```

```
for(ii in 3:3){
  pathway<-names(pvalues)[ii]
  networkslist<-network_seq_correlation(geneset[[pathway]],pbmc_CD4T.
  gglist[[ii]]<-network_plot(networkslist,name1="COVID",name2="Healtl
}
```

```
## [1] "IRLS converged after 5 iterations."
## [1] "13 among 5000 genes have negative variance estimates. Their c
## [1] "0.1293% co-expression estimates were greater than 1 and were
## [1] "0.0751% co-expression estimates were smaller than -1 and were
## [1] "IRLS converged after 3 iterations."
## [1] "8 among 5000 genes have negative variance estimates. Their cc
## [1] "0.0288% co-expression estimates were greater than 1 and were
## [1] "0.0144% co-expression estimates were smaller than -1 and were
```

```
## Using "stress" as default layout
## Using "stress" as default layout
## Using "stress" as default layout
```

```
for(ii in 4:4){
  pathway<-names(pvalues)[ii]
  networkslist<-network_seq_correlation(geneset[[pathway]],pbmc_CD4T.
  gglist[[ii]]<-network_plot(networkslist,name1="COVID",name2="Healtl
}
```

```
## [1] "IRLS converged after 5 iterations."
## [1] "13 among 5000 genes have negative variance estimates. Their c
## [1] "0.1293% co-expression estimates were greater than 1 and were
## [1] "0.0751% co-expression estimates were smaller than -1 and were
## [1] "IRLS converged after 3 iterations."
## [1] "8 among 5000 genes have negative variance estimates. Their cc
## [1] "0.0288% co-expression estimates were greater than 1 and were
## [1] "0.0144% co-expression estimates were smaller than -1 and were
```

```
## Using "stress" as default layout
## Using "stress" as default layout
## Using "stress" as default layout
```

9 9. Show plots

Plot the first pathway.

```
gridExtra::grid.arrange(gglist[[1]]$gg1, gglist[[1]]$gg2, gglist[[1
```

```
## Warning: ggrepel: 7 unlabeled data points (too many overlaps). Cor
## ggrepel: 7 unlabeled data points (too many overlaps). Consider inc
## ggrepel: 7 unlabeled data points (too many overlaps). Consider inc
```

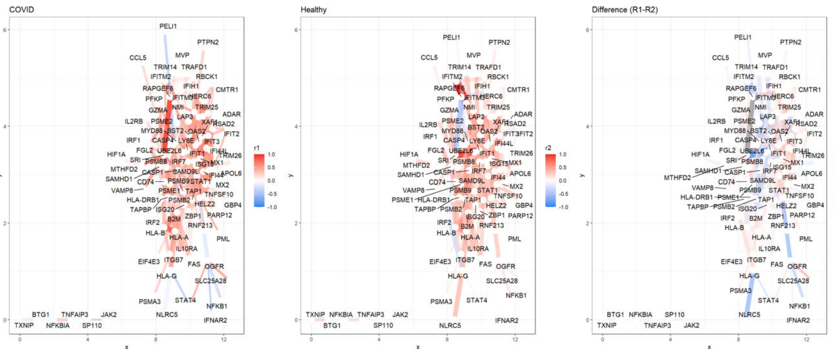

10 10. Show plots

Plot the second pathway.

```
library(ggraph)
gridExtra::grid.arrange(gglist[[2]]$gg1, gglist[[2]]$gg2, gglist[[2
```

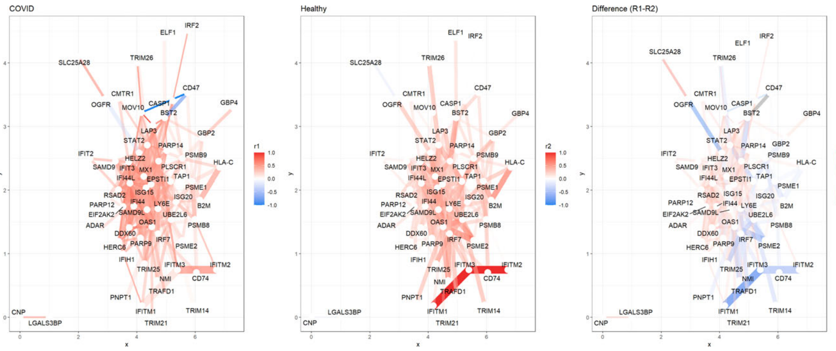

11 11. Show plots

Plot the third pathway.

```
library(ggraph)
gridExtra::grid.arrange(gglist[[3]]$gg1, gglist[[3]]$gg2, gglist[[3
```

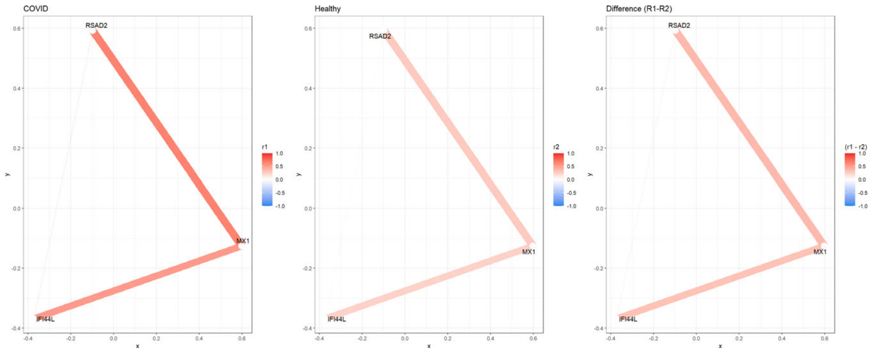

12 12. Show plots

Plot the fourth pathway.

```
library(ggraph)
gridExtra::grid.arrange(gglist[[4]]$gg1, gglist[[4]]$gg2, gglist[[4
```

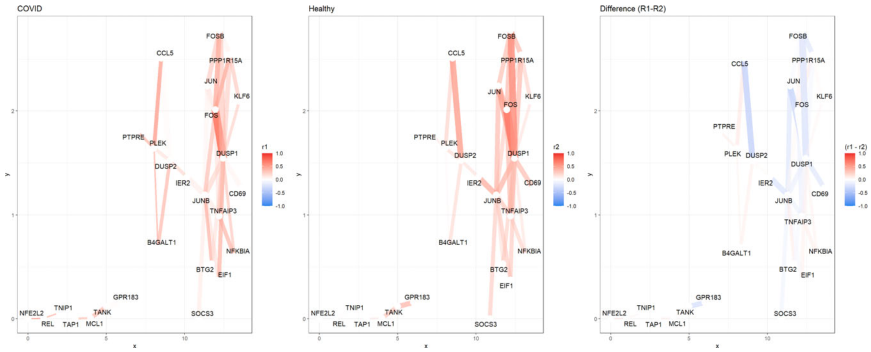

The other Analysis is possible # Session info {.unnumbered}

```
## R version 4.1.2 (2021-11-01)
## Platform: x86_64-w64-mingw32/x64 (64-bit)
## Running under: Windows 10 x64 (build 19045)
##
## Matrix products: default
##
## locale:
## [1] LC_COLLATE=English_United States.1252
## [2] LC_CTYPE=English_United States.1252
## [3] LC_MONETARY=English_United States.1252
## [4] LC_NUMERIC=C
## [5] LC_TIME=English_United States.1252
##
## attached base packages:
## [1] stats4    parallel  stats     graphics  grDevices utils     da
## [8] methods   base
##
## other attached packages:
##  [1] igraph_1.3.5         dplyr_1.1.2          GSEABase_1.56.0
##  [4] graph_1.72.0         annotate_1.72.0      XML_3.99-0.13
##  [7] AnnotationDbi_1.56.2 IRanges_2.28.0       S4Vectors_0.32.4
## [10] Biobase_2.54.0       BiocGenerics_0.40.0  doParallel_1.0.17
## [13] iterators_1.0.14     foreach_1.5.2        gridExtra_2.3
## [16] ggraph_2.1.0         ggplot2_3.4.2        SeuratObject_4.1.3
## [19] Seurat_4.3.0         CSCORE_0.0.0.9000    BiocStyle_2.22.0
##
## loaded via a namespace (and not attached):
##   [1] plyr_1.8.8             lazyeval_0.2.2         sp_1.6-0
##   [4] splines_4.1.2          listenv_0.9.0          scattermore_0.
##   [7] GenomeInfoDb_1.30.1    digest_0.6.31          htmltools_0.5.
##  [10] magick_2.7.3           viridis_0.6.2          fansi_1.0.4
##  [13] memoise_2.0.1          magrittr_2.0.3         tensor_1.5
##  [16] cluster_2.1.4          ROCR_1.0-11            Biostrings_2.6
##  [19] globals_0.16.2         graphlayouts_0.8.4     matrixStats_0.
##  [22] spatstat.sparse_3.0-0  colorspace_2.1-0       blob_1.2.3
##  [25] ggrepel_0.9.3          xfun_0.36              RCurl_1.98-1.9
##  [28] crayon_1.5.2           jsonlite_1.8.4         progressr_0.13
##  [31] spatstat.data_3.0-0    survival_3.5-0         zoo_1.8-11
##  [34] glue_1.6.2             polyclip_1.10-4        gtable_0.3.1
##  [37] zlibbioc_1.40.0        XVector_0.34.0         leiden_0.4.3
##  [40] future.apply_1.10.0    abind_1.4-5            scales_1.2.1
##  [43] DBI_1.1.3              spatstat.random_3.1-3  miniUI_0.1.1.1
##  [46] Rcpp_1.0.10            viridisLite_0.4.1      xtable_1.8-4
##  [49] reticulate_1.28        bit_4.0.5              htmlwidgets_1.
##  [52] httr_1.4.5             RColorBrewer_1.1-3     ellipsis_0.3.2
##  [55] ica_1.0-3              pkgconfig_2.0.3        farver_2.1.1
##  [58] sass_0.4.5             uwot_0.1.14            deldir_1.0-6
##  [61] utf8_1.2.2             labeling_0.4.2         tidyselect_1.2
##  [64] rlang_1.1.0            reshape2_1.4.4         later_1.3.0
##  [67] munsell_0.5.0          tools_4.1.2            cachem_1.0.6
##  [70] cli_3.6.0              RSQLite_2.2.20         generics_0.1.3
##  [73] ggridges_0.5.4         evaluate_0.20          stringr_1.5.0
##  [76] fastmap_1.1.0          yaml_2.3.7             goftest_1.2-3
##  [79] bit64_4.0.5            knitr_1.42             fitdistrplus_1
##  [82] tidygraph_1.2.3        purrr_1.0.1            RANN_2.6.1
##  [85] KEGGREST_1.34.0        pbapply_1.7-0          future_1.30.0
##  [88] nlme_3.1-161           mime_0.12              compiler_4.1.2
```

```
##  [91] rstudioapi_0.14       plotly_4.10.1          png_0.1-8
##  [94] spatstat.utils_3.0-1  tibble_3.2.1           tweenr_2.0.2
##  [97] bslib_0.4.2           stringi_1.7.12         highr_0.10
## [100] lattice_0.20-45       Matrix_1.5-3           vctrs_0.6.1
## [103] pillar_1.9.0          lifecycle_1.0.3        BiocManager_1.
## [106] spatstat.geom_3.0-6   lmtest_0.9-40          jquerylib_0.1.
## [109] RcppAnnoy_0.0.20      bitops_1.0-7           data.table_1.1
## [112] cowplot_1.1.1         irlba_2.3.5.1          httpuv_1.6.8
## [115] patchwork_1.1.2       R6_2.5.1               bookdown_0.33
## [118] promises_1.2.0.1      KernSmooth_2.23-20     parallelly_1.3
## [121] codetools_0.2-18      MASS_7.3-58.2          withr_2.5.0
## [124] sctransform_0.3.5     GenomeInfoDbData_1.2.7 grid_4.1.2
## [127] tidyr_1.3.0           rmarkdown_2.20         Rtsne_0.16
[illegible]
```
