# Supplementary material for: GSNCASCR: An R Package to Identify Differentially Co-Expressed Curated Gene Sets with Single-Cell RNA-Seq Data
Source: Int J Mol Sci. 2025 May 16;26(10):4771. doi: 10.3390/ijms26104771 (PMC12112291; doi:10.3390/ijms26104771)
Supplement: Supplementary file 1 [file ijms-26-04771-s001.zip › SupplementaryFigures.pdf]

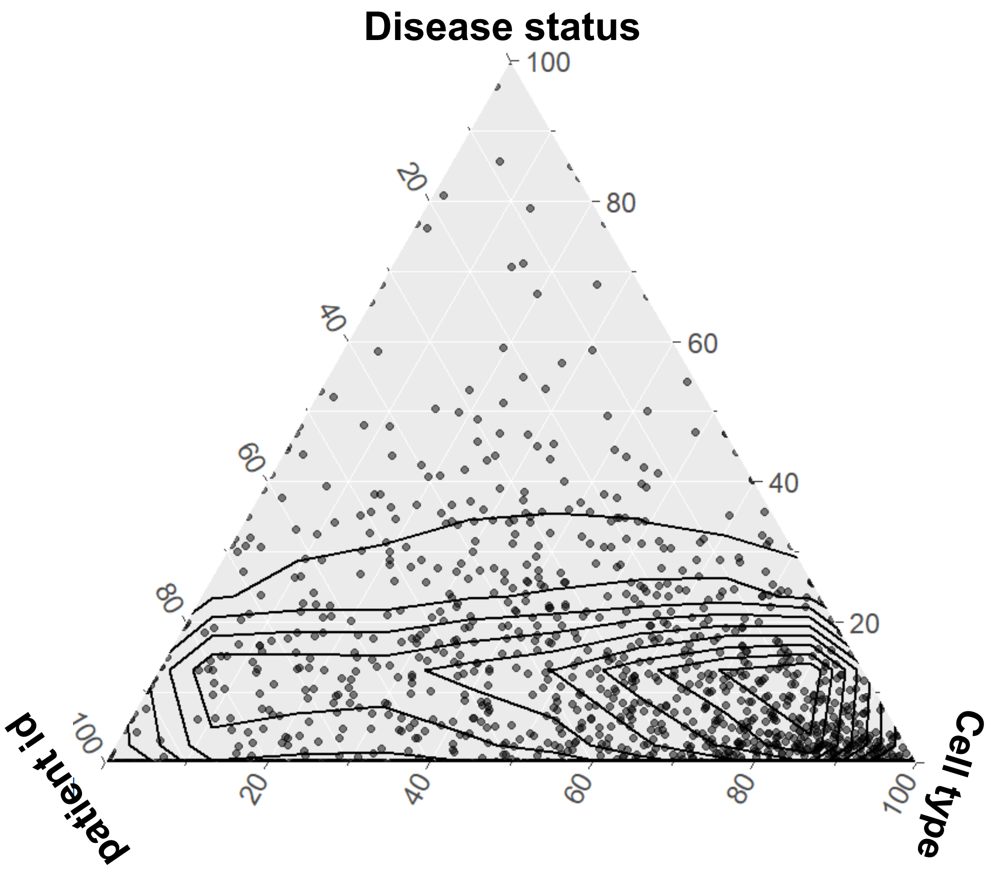

Figure S1. Gene-wise variance partitioning across datasets in COVID-19. Each dot in each ternary plot represents a gene’s relative amount of variance explained (by patient id, status, and cell type).

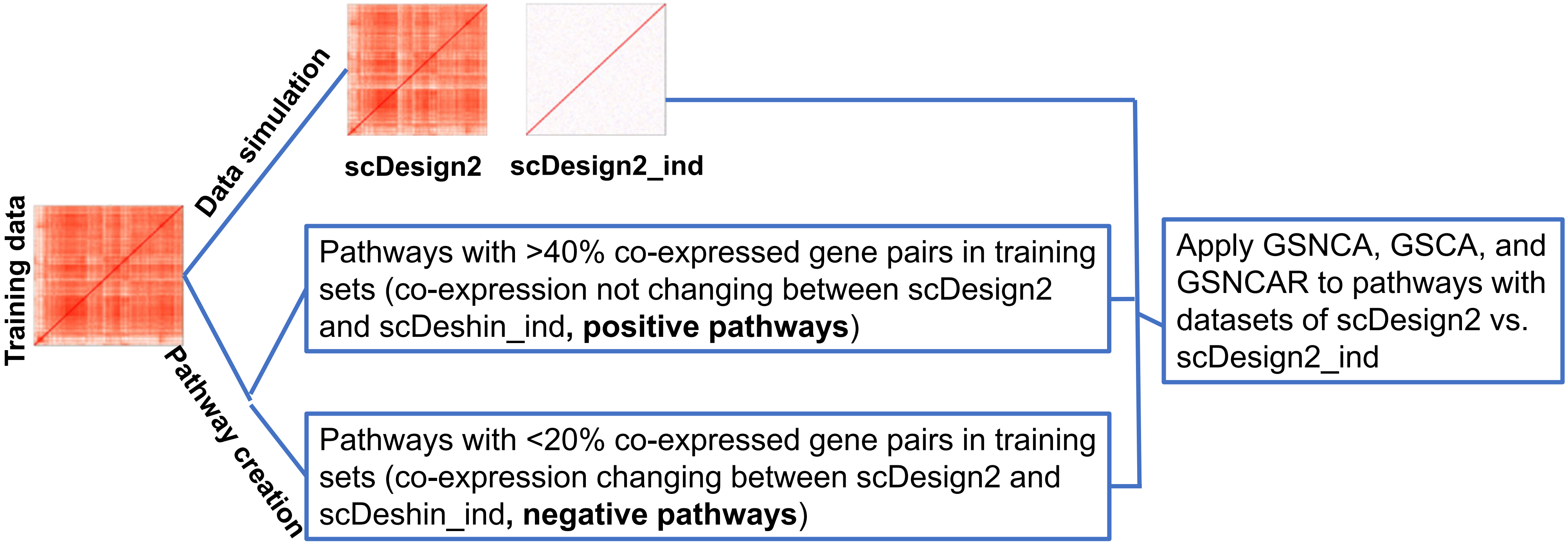

Figure S2. A pipeline for data simulation. 1) Based on the training dataset, two single-cell datasets were generated. 2) Created pathways of varying sizes (20, 40, 60, 80, and 100) by selecting different numbers of highly correlated gene pairs from the initial training dataset. 3) GSNCASCR, GSNCA, and GSCA on datasets with and without maintained co-expression.

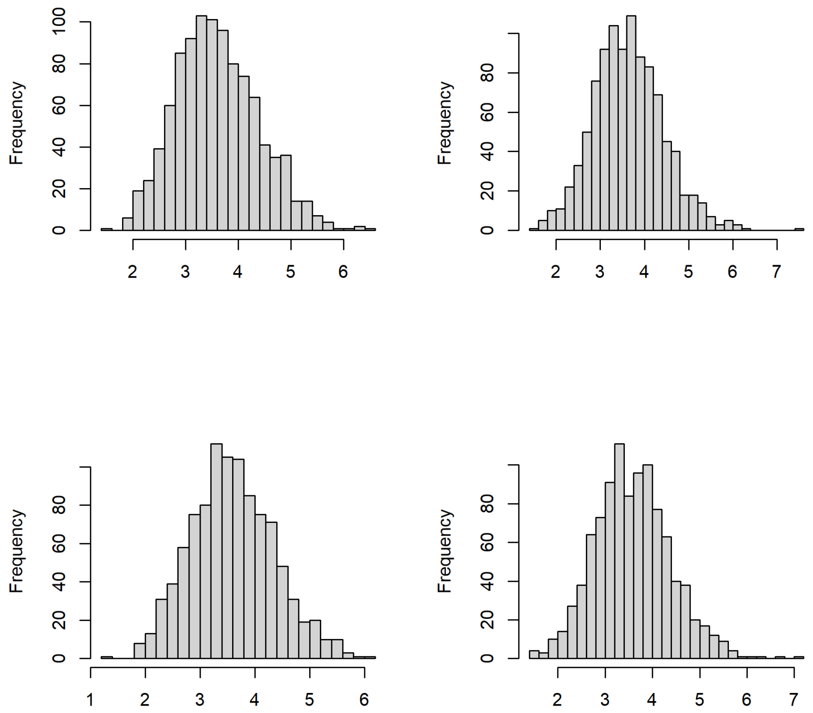

Figure S3. Histogram showing the distribution of w values for four representative pathways.

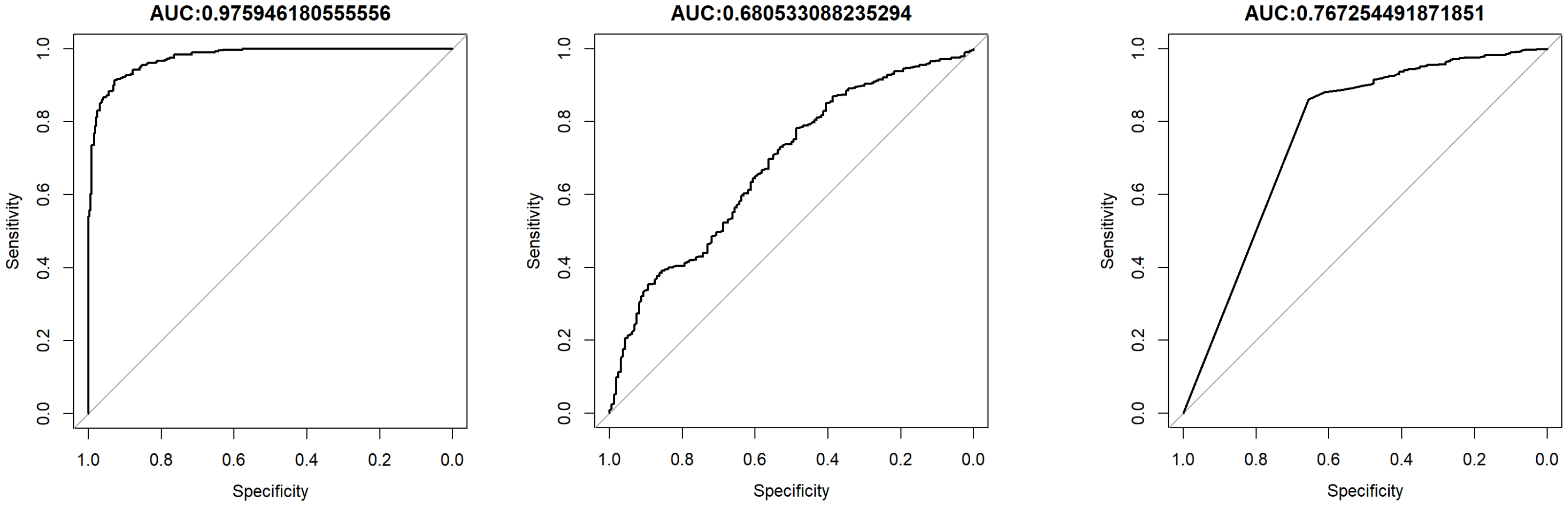

GSNCASCR GSNCA GSAC

Figure S4. AUC values with >=40% for positive pathways and <40% for negative pathways.

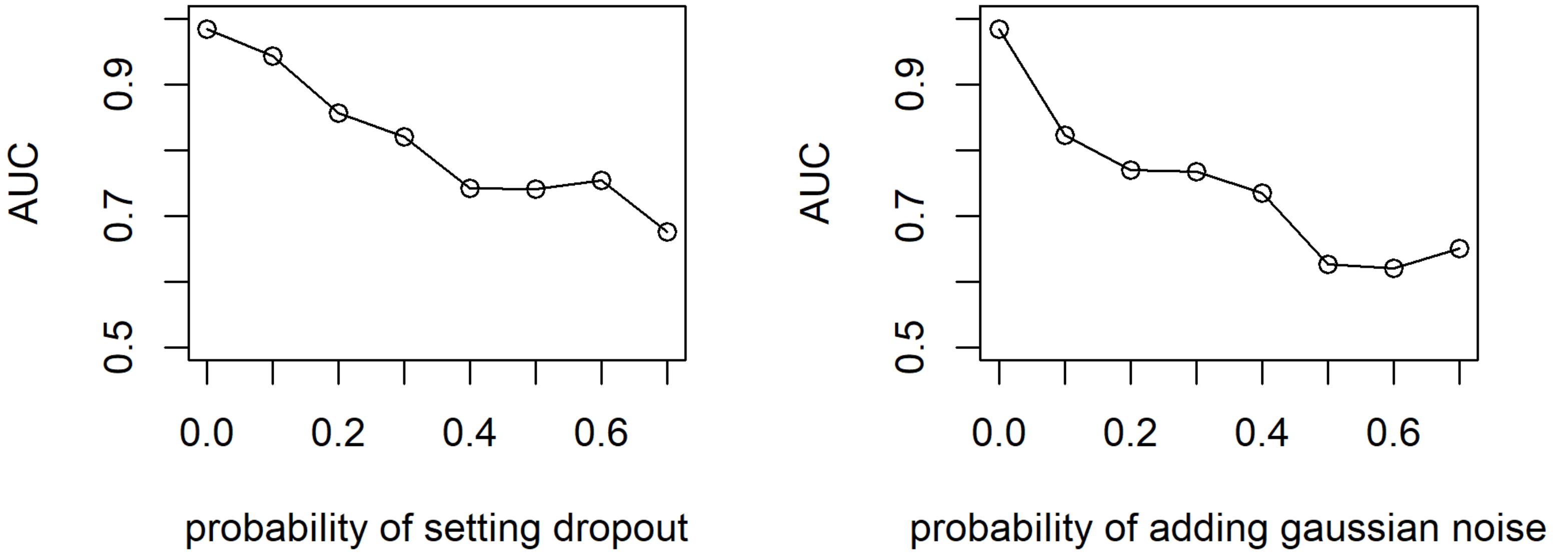

Figure S5. AUC values decreasing with noise level increasing.
