# Supplementary material for: GSNCASCR: An R Package to Identify Differentially Co-Expressed Curated Gene Sets with Single-Cell RNA-Seq Data
Source: Int J Mol Sci. 2025 May 16;26(10):4771. doi: 10.3390/ijms26104771 (PMC12112291; doi:10.3390/ijms26104771)
Supplement: Supplementary file 1 [file ijms-26-04771-s001.zip › Table S1-GO enrichment analysis of B cells.pdf]

Table S1. Gene Ontology enrichment analysis of B cells.

GO term	*P* value
GOBP_CELL_ACTIVATION	2.06E-28
GOBP_POSITIVE_REGULATION_OF_MULTICELLULAR_ORGANISMAL_PROCESS	1.57E-27
GOBP_IMMUNE_EFFECTOR_PROCESS	2.20E-27
GOBP_LEUKOCYTE_MEDIATED_CYTOTOXICITY	4.11E-27
GOBP_LYMPHOCYTE_ACTIVATION	1.71E-26
GOBP_CELL_CELL_ADHESION	2.06E-26
GOBP_T_CELL_ACTIVATION	2.56E-26
GOBP_CELL_ADHESION	3.27E-26
GOBP_POSITIVE_REGULATION_OF_CELL_ACTIVATION	4.88E-26
GOBP_REGULATION_OF_CELL_ACTIVATION	1.13E-25
GOBP_CELL_KILLING	4.05E-25
GOBP_POSITIVE_REGULATION_OF_CELL_ADHESION	4.91E-25
GOBP_POSITIVE_REGULATION_OF_IMMUNE_SYSTEM_PROCESS	5.20E-25
GOBP_ADAPTIVE_IMMUNE_RESPONSE	6.25E-25
GOBP_POSITIVE_REGULATION_OF_CELL_CELL_ADHESION	7.36E-25
GOBP_POSITIVE_REGULATION_OF_IMMUNE_RESPONSE	9.18E-25
GOBP_HEMOPOIESIS	1.42E-24
GOBP_REGULATION_OF_CELL_CELL_ADHESION	2.09E-24
GOBP_LEUKOCYTE_PROLIFERATION	2.19E-24
GOBP_LEUKOCYTE_CELL_CELL_ADHESION	2.34E-24
GOBP_MONONUCLEAR_CELL_DIFFERENTIATION	2.52E-24
GOBP_POSITIVE_REGULATION_OF_LEUKOCYTE_CELL_CELL_ADHESION	3.07E-24
GOBP_POSITIVE_REGULATION_OF_LYMPHOCYTE_ACTIVATION	5.39E-24
GOBP_REGULATION_OF_IMMUNE_RESPONSE	5.50E-24
GOBP_LEUKOCYTE_DIFFERENTIATION	6.59E-24
GOBP_RESPONSE_TO_ENDOGENOUS_STIMULUS	7.44E-24
GOBP_ALPHA_BETA_T_CELL_ACTIVATION	1.52E-23
GOBP_REGULATION_OF_LYMPHOCYTE_ACTIVATION	1.70E-23
GOBP_LOCOMOTION	2.20E-23
GOBP_REGULATION_OF_CELL_DEVELOPMENT	2.26E-23
GOBP_T_CELL_DIFFERENTIATION	2.87E-23
GOBP_LEUKOCYTE_MEDIATED_IMMUNITY	5.03E-23
GOBP_POSITIVE_REGULATION_OF_GENE_EXPRESSION	5.58E-23
GOBP_REGULATION_OF_CELL_ADHESION	7.93E-23
GOBP_REGULATION_OF_T_CELL_ACTIVATION	1.01E-22
GOBP_B_CELL_ACTIVATION	1.08E-22
GOBP_LYMPHOCYTE_MEDIATED_IMMUNITY	1.30E-22
GOBP_HUMORAL_IMMUNE_RESPONSE	1.40E-22
GOBP_NEGATIVE_REGULATION_OF_MULTICELLULAR_ORGANISMAL_PROCESS	1.46E-22
GOBP_RESPONSE_TO_BACTERIUM	1.47E-22
GOBP_REGULATION_OF_HEMOPOIESIS	1.82E-22
GOBP_HOMEOSTATIC_PROCESS	2.05E-22
GOBP_DEFENSE_RESPONSE_TO_OTHER_ORGANISM	2.17E-22
GOBP_POSITIVE_REGULATION_OF_TRANSCRIPTION_BY_RNA_POLYMERASE_II	2.48E-22
GOBP_REGULATION_OF_MULTICELLULAR_ORGANISMAL_DEVELOPMENT	2.66E-22
GOBP_NEGATIVE_REGULATION_OF_CELL_ACTIVATION	3.13E-22
GOBP_CELLULAR_RESPONSE_TO_SALT	4.70E-22
GOBP_RESPONSE_TO_HORMONE	5.28E-22
GOBP_CELL_MOTILITY	5.50E-22
GOBP_ADAPTIVE_IMMUNE_RESPONSE_BASED_ON_SOMATIC_RECOMBINATION_OF_IMMUNE_RECEPTORS_ BUILT_FROM_IMMUNOGLOBULIN_SUPERFAMILY_DOMAINS	5.70E-22
GOBP_RIBOSOME_BIOGENESIS	6.50E-22
GOBP_CD4_POSITIVE_ALPHA_BETA_T_CELL_ACTIVATION	7.44E-22
GOBP_PROTEIN_PHOSPHORYLATION	9.55E-22
GOBP_RESPONSE_TO_LIPID	9.64E-22
GOBP_LYMPHOCYTE_ACTIVATION_INVOLVED_IN_IMMUNE_RESPONSE	1.02E-21
GOBP_B_CELL_MEDIATED_IMMUNITY	1.07E-21
GOBP_NEGATIVE_REGULATION_OF_RNA_BIOSYNTHETIC_PROCESS	1.12E-21
GOBP_INNATE_IMMUNE_RESPONSE	1.13E-21

GOBP_ACTIVATION_OF_IMMUNE_RESPONSE	1.21E-21
GOBP_REGULATION_OF_ALPHA_BETA_T_CELL_ACTIVATION	1.22E-21
GOBP_ANTIGEN_RECEPTOR_MEDIATED_SIGNALING_PATHWAY	1.28E-21
GOBP_CELLULAR_RESPONSE_TO_OXYGEN_CONTAINING_COMPOUND	1.49E-21
GOBP_RESPONSE_TO_CYTOKINE	1.50E-21
GOBP_REGULATION_OF_IMMUNE_EFFECTOR_PROCESS	1.53E-21
GOBP_REGULATION_OF_LOCOMOTION	1.64E-21
GOBP_REGULATION_OF_LEUKOCYTE_PROLIFERATION	2.11E-21
GOBP_PLASMA_MEMBRANE_ORGANIZATION	2.31E-21
GOBP_CYTOKINE_PRODUCTION	2.39E-21
GOBP_CELL_ACTIVATION_INVOLVED_IN_IMMUNE_RESPONSE	2.46E-21
GOBP_POSITIVE_REGULATION_OF_DEVELOPMENTAL_PROCESS	2.68E-21
GOBP_REGULATION_OF_LEUKOCYTE_DIFFERENTIATION	2.75E-21
GOBP_IMMUNE_RESPONSE_REGULATING_SIGNALING_PATHWAY	3.52E-21
GOBP_ANTIGEN_PROCESSING_AND_PRESENTATION	3.69E-21
GOBP_INFLAMMATORY_RESPONSE	4.38E-21
GOBP_INTRACELLULAR_PROTEIN_TRANSPORT	4.61E-21
GOBP_RESPONSE_TO_ENDOPLASMIC_RETICULUM_STRESS	4.88E-21
GOBP_ALPHA_BETA_T_CELL_DIFFERENTIATION	7.37E-21
GOBP_POSITIVE_REGULATION_OF_CELL_POPULATION_PROLIFERATION	8.41E-21
GOBP_MYELOID_CELL_DIFFERENTIATION	8.69E-21
GOBP_LEUKOCYTE_CHEMOTAXIS	1.00E-20
GOBP_REGULATION_OF_CELL_DIFFERENTIATION	1.02E-20
GOBP_RESPONSE_TO_GROWTH_FACTOR	1.40E-20
GOBP_TAXIS	1.44E-20
GOBP_PROTEIN_TARGETING	1.59E-20
GOBP_T_CELL_MEDIATED_IMMUNITY	1.76E-20
GOBP_ANTIGEN_PROCESSING_AND_PRESENTATION_OF_PEPTIDE_ANTIGEN	2.17E-20
GOBP_PEPTIDE_BIOSYNTHETIC_PROCESS	2.43E-20
GOBP_POSITIVE_REGULATION_OF_IMMUNE_EFFECTOR_PROCESS	2.73E-20
GOBP_PEPTIDE_METABOLIC_PROCESS	3.59E-20
GOBP_NEGATIVE_REGULATION_OF_IMMUNE_SYSTEM_PROCESS	4.20E-20
GOBP_RESPONSE_TO_NITROGEN_COMPOUND	4.26E-20
GOBP_MONOATOMIC_CATION_TRANSPORT	4.47E-20
GOBP_REGULATION_OF_CELL_KILLING	4.53E-20
GOBP_STRIATED_MUSCLE_CELL_DIFFERENTIATION	4.76E-20
GOBP_MULTICELLULAR_ORGANISMAL_LEVEL_HOMEOSTASIS	4.88E-20
GOBP_MONOATOMIC_ION_TRANSPORT	5.01E-20
GOBP_PHOSPHORYLATION	6.38E-20
GOBP_B_CELL_PROLIFERATION	7.05E-20
GOBP_NEGATIVE_REGULATION_OF_CELL_POPULATION_PROLIFERATION	1.06E-19
GOBP_CELL_CHEMOTAXIS	1.12E-19
GOBP_PROTEIN_LOCALIZATION_TO_ORGANELLE	1.22E-19
GOBP_IMMUNE_RESPONSE_REGULATING_CELL_SURFACE_RECEPTOR_SIGNALING_PATHWAY	1.26E-19
GOBP_NEGATIVE_REGULATION_OF_SIGNALING	1.31E-19
GOBP_APOPTOTIC_SIGNALING_PATHWAY	1.31E-19
GOBP_TUMOR_NECROSIS_FACTOR_SUPERFAMILY_CYTOKINE_PRODUCTION	1.37E-19
GOBP_NEGATIVE_REGULATION_OF_GENE_EXPRESSION	1.40E-19
GOBP_REGULATION_OF_RESPONSE_TO_EXTERNAL_STIMULUS	1.54E-19
GOBP_REGULATION_OF_LYMPHOCYTE_DIFFERENTIATION	1.76E-19
GOBP_POSITIVE_REGULATION_OF_LEUKOCYTE_PROLIFERATION	1.86E-19
GOBP_REGULATION_OF_CATALYTIC_ACTIVITY	1.91E-19
GOBP_SECRETION	2.07E-19
GOBP_POSITIVE_REGULATION_OF_CELL_DEVELOPMENT	2.22E-19
GOBP_POSITIVE_REGULATION_OF_HEMOPOIESIS	2.44E-19
GOBP_REGULATION_OF_B_CELL_ACTIVATION	2.81E-19
GOBP_B_CELL_RECEPTOR_SIGNALING_PATHWAY	3.19E-19
GOBP_NEGATIVE_REGULATION_OF_TRANSCRIPTION_BY_RNA_POLYMERASE_II	3.43E-19
GOBP_POSITIVE_REGULATION_OF_CYTOKINE_PRODUCTION	4.31E-19
GOBP_ACTIN_FILAMENT_BASED_PROCESS	4.34E-19
GOBP_POSITIVE_REGULATION_OF_GROWTH	4.71E-19
GOBP_AMIDE_BIOSYNTHETIC_PROCESS	5.30E-19
GOBP_RESPONSE_TO_MOLECULE_OF_BACTERIAL_ORIGIN	5.66E-19
GOBP_RRNA_METABOLIC_PROCESS	6.11E-19

GOBP_NEGATIVE_REGULATION_OF_PROTEIN_METABOLIC_PROCESS	6.74E-19
GOBP_POSITIVE_REGULATION_OF_RESPONSE_TO_EXTERNAL_STIMULUS	7.59E-19
GOBP_POSITIVE_REGULATION_OF_MOLECULAR_FUNCTION	7.68E-19
GOBP_REGULATION_OF_PHOSPHORUS_METABOLIC_PROCESS	8.11E-19
GOBP_REGULATION_OF_PROTEIN_MODIFICATION_PROCESS	8.58E-19
GOBP_NEGATIVE_REGULATION_OF_LYMPHOCYTE_ACTIVATION	8.79E-19
GOBP_REGULATION_OF_DEFENSE_RESPONSE	9.11E-19
GOBP_LEUKOCYTE_MIGRATION	9.55E-19
GOBP_CELL_MORPHOGENESIS	9.60E-19
GOBP_EXPORT_FROM_CELL	9.68E-19
GOBP_MYELOID_LEUKOCYTE_MIGRATION	1.05E-18
GOBP_REGULATION_OF_HYDROLASE_ACTIVITY	1.06E-18
GOBP_T_CELL_ACTIVATION_INVOLVED_IN_IMMUNE_RESPONSE	1.07E-18
GOBP_CYTOSKELETON_ORGANIZATION	1.07E-18
GOBP_NEGATIVE_REGULATION_OF_PROGRAMMED_CELL_DEATH	1.09E-18
GOBP_PRODUCTION_OF_MOLECULAR_MEDIATOR_OF_IMMUNE_RESPONSE	1.17E-18
GOBP_REGULATION_OF_DEVELOPMENTAL_GROWTH	1.17E-18
GOBP_CELLULAR_RESPONSE_TO_EXTERNAL_STIMULUS	1.20E-18
GOBP_RESPONSE_TO_TYPE_II_INTERFERON	1.28E-18
GOBP_CYTOPLASMIC_TRANSLATION	1.32E-18
GOBP_CANONICAL_NF_KAPPAB_SIGNAL_TRANSDUCTION	1.37E-18
GOBP_DEVELOPMENTAL_GROWTH	1.46E-18
GOBP_ENZYME_LINKED_RECEPTOR_PROTEIN_SIGNALING_PATHWAY	1.46E-18
GOBP_REGULATION_OF_MYELOID_CELL_DIFFERENTIATION	1.49E-18
GOBP_T_CELL_PROLIFERATION	1.54E-18
GOBP_POSITIVE_REGULATION_OF_T_CELL_PROLIFERATION	1.73E-18
GOBP_REGULATION_OF_MYELOID_LEUKOCYTE_DIFFERENTIATION	1.79E-18
GOBP_MUSCLE_CELL_DIFFERENTIATION	2.00E-18
GOBP_REGULATION_OF_RESPONSE_TO_ENDOPLASMIC_RETICULUM_STRESS	2.03E-18
GOBP_MUSCLE_STRUCTURE_DEVELOPMENT	2.29E-18
GOBP_TRANSMEMBRANE_TRANSPORT	2.34E-18
GOBP_GROWTH	2.47E-18
GOBP_AMIDE_METABOLIC_PROCESS	2.74E-18
GOBP_RESPONSE_TO_ORGANIC_CYCLIC_COMPOUND	2.77E-18
GOBP_HOMEOSTASIS_OF_NUMBER_OF_CELLS	2.86E-18
GOBP_RESPONSE_TO_TOPOLOGICALLY_INCORRECT_PROTEIN	2.95E-18
GOBP_RESPONSE_TO_VIRUS	3.43E-18
GOBP_ESTABLISHMENT_OF_PROTEIN_LOCALIZATION_TO_ORGANELLE	4.70E-18
GOBP_REGULATION_OF_GROWTH	5.23E-18
GOBP_POSITIVE_REGULATION_OF_INTRACELLULAR_SIGNAL_TRANSDUCTION	5.58E-18
GOBP_CIRCULATORY_SYSTEM_PROCESS	6.03E-18
GOBP_REGULATION_OF_DNA_BINDING_TRANSCRIPTION_FACTOR_ACTIVITY	6.37E-18
GOBP_REGULATION_OF_TRANSPORT	6.51E-18
GOBP_REGULATION_OF_CELLULAR_COMPONENT_BIOGENESIS	6.60E-18
GOBP_INTERLEUKIN_6_PRODUCTION	6.91E-18
GOBP_PROTEIN_LOCALIZATION_TO_ENDOPLASMIC_RETICULUM	7.51E-18
GOBP_POSITIVE_REGULATION_OF_LOCOMOTION	8.64E-18
GOBP_MONOATOMIC_CATION_TRANSMEMBRANE_TRANSPORT	9.92E-18
GOBP_REGULATION_OF_PHOSPHORYLATION	1.08E-17
GOBP_RIBONUCLEOPROTEIN_COMPLEX_BIOGENESIS	1.27E-17
GOBP_SKELETAL_SYSTEM_DEVELOPMENT	1.43E-17
GOBP_POSITIVE_REGULATION_OF_CELL_DIFFERENTIATION	1.73E-17
GOBP_RESPONSE_TO_INORGANIC_SUBSTANCE	1.83E-17
GOBP_CELL_JUNCTION_ORGANIZATION	1.87E-17
GOBP_MEMBRANE_ORGANIZATION	2.07E-17
GOBP_GLYCOPROTEIN_METABOLIC_PROCESS	2.36E-17
GOBP_POSITIVE_REGULATION_OF_LYMPHOCYTE_DIFFERENTIATION	2.39E-17
GOBP_B_CELL_DIFFERENTIATION	2.65E-17
GOBP_CIRCULATORY_SYSTEM_DEVELOPMENT	2.83E-17
GOBP_SMALL_GTPASE_MEDIATED_SIGNAL_TRANSDUCTION	2.85E-17
GOBP_REGULATION_OF_INFLAMMATORY_RESPONSE	2.86E-17
GOBP_RESPONSE_TO_SALT	2.95E-17
GOBP_ENDOMEMBRANE_SYSTEM_ORGANIZATION	3.11E-17
GOBP_MYELOID_CELL_HOMEOSTASIS	3.29E-17

GOBP_POSITIVE_REGULATION_OF_PROGRAMMED_CELL_DEATH	3.32E-17
GOBP_POSITIVE_REGULATION_OF_LEUKOCYTE_MIGRATION	3.89E-17
GOBP_PROTEOLYSIS_INVOLVED_IN_PROTEIN_CATABOLIC_PROCESS	4.09E-17
GOBP_REGULATION_OF_AMIDE_METABOLIC_PROCESS	4.33E-17
GOBP_CYTOKINE_MEDIATED_SIGNALING_PATHWAY	4.55E-17
GOBP_MONOATOMIC_ION_TRANSMEMBRANE_TRANSPORT	4.57E-17
GOBP_NCRNA_METABOLIC_PROCESS	4.87E-17
GOBP_REGULATION_OF_TRANSFERASE_ACTIVITY	5.16E-17
GOBP_T_CELL_DIFFERENTIATION_IN_THYMUS	5.35E-17
GOBP_RESPONSE_TO_METAL_ION	5.58E-17
GOBP_REGULATION_OF_PROTEIN_STABILITY	5.73E-17
GOBP_NEGATIVE_REGULATION_OF_CELL_DEVELOPMENT	5.86E-17
GOBP_T_CELL_RECEPTOR_SIGNALING_PATHWAY	6.38E-17
GOBP_NEUROGENESIS	6.51E-17
GOBP_POSITIVE_REGULATION_OF_LEUKOCYTE_MEDIATED_IMMUNITY	6.70E-17
GOBP_CELLULAR_RESPONSE_TO_LIPID	6.88E-17
GOBP_MYELOID_LEUKOCYTE_ACTIVATION	6.91E-17
GOBP_POSITIVE_REGULATION_OF_LYMPHOCYTE_MEDIATED_IMMUNITY	7.01E-17
GOBP_CELLULAR_RESPONSE_TO_HORMONE_STIMULUS	7.60E-17
GOBP_REGULATION_OF_LEUKOCYTE_MIGRATION	8.82E-17
GOBP_REGULATION_OF_ACTIN_FILAMENT_BASED_PROCESS	9.01E-17
GOBP_NEGATIVE_REGULATION_OF_ORGANELLE_ORGANIZATION	9.93E-17
GOBP_AMIDE_TRANSPORT	9.95E-17
GOBP_PROTEIN_CATABOLIC_PROCESS	1.02E-16
GOBP_NEGATIVE_REGULATION_OF_CELL_DIFFERENTIATION	1.04E-16
GOBP_NEGATIVE_REGULATION_OF_AMIDE_METABOLIC_PROCESS	1.06E-16
GOBP_NEGATIVE_REGULATION_OF_DEVELOPMENTAL_PROCESS	1.08E-16
GOBP_REGULATION_OF_CHEMOTAXIS	1.33E-16
GOBP_CELL_CELL_SIGNALING	1.40E-16
GOBP_CELLULAR_RESPONSE_TO_INORGANIC_SUBSTANCE	1.42E-16
GOBP_VASCULAR_PROCESS_IN_CIRCULATORY_SYSTEM	1.50E-16
GOBP_CELL_PROJECTION_ORGANIZATION	1.54E-16
GOBP_IMMUNOGLOBULIN_PRODUCTION	1.54E-16
GOBP_CALCIUM_ION_HOMEOSTASIS	1.78E-16
GOBP_POSITIVE_REGULATION_OF_B_CELL_ACTIVATION	1.85E-16
GOBP_REGULATION_OF_ACTIN_FILAMENT_LENGTH	1.92E-16
GOBP_BIOLOGICAL_PROCESS_INVOLVED_IN_SYMBIOTIC_INTERACTION	2.05E-16
GOBP_INORGANIC_ION_HOMEOSTASIS	2.08E-16
GOBP_CHEMICAL_HOMEOSTASIS	2.08E-16
GOBP_POSITIVE_REGULATION_OF_DEFENSE_RESPONSE	2.15E-16
GOBP_POSITIVE_REGULATION_OF_CATALYTIC_ACTIVITY	2.28E-16
GOBP_PROCESS_UTILIZING_AUTOPHAGIC_MECHANISM	2.53E-16
GOBP_RIBOSOMAL_SMALL_SUBUNIT_BIOGENESIS	2.61E-16
GOBP_REGULATION_OF_CELL_CYCLE	2.74E-16
GOBP_PROTON_TRANSMEMBRANE_TRANSPORT	2.90E-16
GOBP_REGULATION_OF_ORGANELLE_ORGANIZATION	3.14E-16
GOBP_NCRNA_PROCESSING	3.20E-16
GOBP_DEVELOPMENTAL_CELL_GROWTH	3.29E-16
GOBP_REGULATION_OF_LEUKOCYTE_MEDIATED_IMMUNITY	3.40E-16
GOBP_CELLULAR_RESPONSE_TO_DECREASED_OXYGEN_LEVELS	3.68E-16
GOBP_POSITIVE_REGULATION_OF_CANONICAL_NF_KAPPAB_SIGNAL_TRANSDUCTION	3.76E-16
GOBP_NEGATIVE_REGULATION_OF_MOLECULAR_FUNCTION	3.98E-16
GOBP_POSITIVE_REGULATION_OF_TUMOR_NECROSIS_FACTOR_SUPERFAMILY_CYTOKINE_PRODUCTION	4.07E-16
GOBP_ENTRY_INTO_HOST	4.23E-16
GOBP_NEGATIVE_REGULATION_OF_RESPONSE_TO_EXTERNAL_STIMULUS	4.31E-16
GOBP_NEGATIVE_REGULATION_OF_PROTEIN_CONTAINING_COMPLEX_ASSEMBLY	4.42E-16
GOBP_LOCALIZATION_WITHIN_MEMBRANE	4.53E-16
GOBP_REGULATION_OF_CELLULAR_LOCALIZATION	4.77E-16
GOBP_ERYTHROCYTE_HOMEOSTASIS	4.84E-16
GOBP_GENERATION_OF_NEURONS	4.99E-16
GOBP_MYELOID_LEUKOCYTE_DIFFERENTIATION	5.04E-16
GOBP_RESPONSE_TO_OXIDATIVE_STRESS	6.34E-16
GOBP_POSITIVE_REGULATION_OF_CELLULAR_COMPONENT_ORGANIZATION	7.52E-16
GOBP_REGULATION_OF_APOPTOTIC_SIGNALING_PATHWAY	7.73E-16

GOBP_POSITIVE_REGULATION_OF_NF_KAPPAB_TRANSCRIPTION_FACTOR_ACTIVITY	7.93E-16
GOBP_MULTI_MULTICELLULAR_ORGANISM_PROCESS	8.73E-16
GOBP_CHROMATIN_REMODELING	9.19E-16
GOBP_REGULATION_OF_ANATOMICAL_STRUCTURE_SIZE	9.60E-16
GOBP_CHROMOSOME_ORGANIZATION	9.78E-16
GOBP_RESPONSE_TO_EXTRACELLULAR_STIMULUS	1.00E-15
GOBP_CELLULAR_RESPONSE_TO_EXTRACELLULAR_STIMULUS	1.04E-15
GOBP_RESPONSE_TO_PEPTIDE	1.07E-15
GOBP_VIRAL_PROCESS	1.09E-15
GOBP_VIRAL_LIFE_CYCLE	1.26E-15
GOBP_REGULATION_OF_SUPRAMOLECULAR_FIBER_ORGANIZATION	1.26E-15
GOBP_NEGATIVE_REGULATION_OF_CELLULAR_COMPONENT_ORGANIZATION	1.39E-15
GOBP_RESPONSE_TO_XENOBIOTIC_STIMULUS	1.42E-15
GOBP_MULTICELLULAR_ORGANISM_GROWTH	1.43E-15
GOBP_PROTEIN_DNA_COMPLEX_ORGANIZATION	1.61E-15
GOBP_WOUND_HEALING	1.81E-15
GOBP_GLYCOPROTEIN_BIOSYNTHETIC_PROCESS	1.82E-15
GOBP_NEGATIVE_REGULATION_OF_INTRACELLULAR_SIGNAL_TRANSDUCTION	1.83E-15
GOBP_MAINTENANCE_OF_LOCATION_IN_CELL	1.86E-15
GOBP_REGULATED_EXOCYTOSIS	2.01E-15
GOBP_REGULATION_OF_ACTIN_FILAMENT_ORGANIZATION	2.08E-15
GOBP_MONONUCLEAR_CELL_MIGRATION	2.23E-15
GOBP_CELLULAR_RESPONSE_TO_BIOTIC_STIMULUS	2.38E-15
GOBP_ORGANIC_HYDROXY_COMPOUND_TRANSPORT	2.39E-15
GOBP_RESPONSE_TO_ABIOTIC_STIMULUS	2.47E-15
GOBP_NEGATIVE_REGULATION_OF_IMMUNE_RESPONSE	2.47E-15
GOBP_NEGATIVE_REGULATION_OF_INFLAMMATORY_RESPONSE	2.58E-15
GOBP_POSITIVE_REGULATION_OF_TRANSFERASE_ACTIVITY	2.79E-15
GOBP_POSITIVE_REGULATION_OF_PROTEIN_LOCALIZATION_TO_NUCLEUS	2.87E-15
GOBP_REGULATION_OF_NEURON_APOPTOTIC_PROCESS	2.95E-15
GOBP_REGULATION_OF_PROTEIN_CONTAINING_COMPLEX_ASSEMBLY	3.22E-15
GOBP_REGULATION_OF_DNA_BIOSYNTHETIC_PROCESS	3.24E-15
GOBP_REGULATION_OF_SECRETION	3.30E-15
GOBP_SYNAPSE_ORGANIZATION	3.34E-15
GOBP_ACTIN_FILAMENT_ORGANIZATION	3.36E-15
GOBP_LIPID_METABOLIC_PROCESS	3.57E-15
GOBP_DNA_METABOLIC_PROCESS	3.70E-15
GOBP_NEURON_APOPTOTIC_PROCESS	4.04E-15
GOBP_REGULATION_OF_ANATOMICAL_STRUCTURE_MORPHOGENESIS	4.08E-15
GOBP_REGULATION_OF_PROTEIN_CONTAINING_COMPLEX_DISASSEMBLY	4.18E-15
GOBP_CELLULAR_RESPONSE_TO_OXIDATIVE_STRESS	4.22E-15
GOBP_NEURON_PROJECTION_EXTENSION	4.27E-15
GOBP_ANIMAL_ORGAN_MORPHOGENESIS	4.44E-15
GOBP_RESPONSE_TO_STEROID_HORMONE	4.53E-15
GOBP_MAINTENANCE_OF_LOCATION	4.79E-15
GOBP_INSULIN_SECRETION	5.20E-15
GOBP_TELOMERE_MAINTENANCE_VIA_TELOMERE_LENGTHENING	5.25E-15
GOBP_REGULATION_OF_CELLULAR_COMPONENT_SIZE	5.50E-15
GOBP_POSITIVE_REGULATION_OF_PROTEIN_MODIFICATION_PROCESS	5.58E-15
GOBP_MAPK_CASCADE	5.65E-15
GOBP_NEGATIVE_REGULATION_OF_PROTEIN_LOCALIZATION	6.25E-15
GOBP_NEGATIVE_REGULATION_OF_CELL_ADHESION	6.59E-15
GOBP_REGULATION_OF_KINASE_ACTIVITY	6.68E-15
GOBP_RIBOSOMAL_LARGE_SUBUNIT_BIOGENESIS	6.78E-15
GOBP_REGULATION_OF_HORMONE_LEVELS	6.84E-15
GOBP_ACTIN_POLYMERIZATION_OR_DEPOLYMERIZATION	7.66E-15
GOBP_POSITIVE_REGULATION_OF_DNA_BINDING_TRANSCRIPTION_FACTOR_ACTIVITY	8.06E-15
GOBP_CELLULAR_LIPID_METABOLIC_PROCESS	8.11E-15
GOBP_LEUKOCYTE_HOMEOSTASIS	8.69E-15
GOBP_POSITIVE_REGULATION_OF_ADAPTIVE_IMMUNE_RESPONSE	8.72E-15
GOBP_POSITIVE_REGULATION_OF_PROTEIN_METABOLIC_PROCESS	9.00E-15
GOBP_REGULATION_OF_NEURON_DIFFERENTIATION	9.26E-15
GOBP_ESTABLISHMENT_OF_PROTEIN_LOCALIZATION_TO_MEMBRANE	9.78E-15
GOBP_PROTEIN_TARGETING_TO_MEMBRANE	1.01E-14

GOBP_MONOATOMIC_ION_HOMEOSTASIS	1.03E-14
GOBP_NEGATIVE_REGULATION_OF_IMMUNE_EFFECTOR_PROCESS	1.07E-14
GOBP_PEPTIDYL_TYROSINE_MODIFICATION	1.09E-14
GOBP_RNA_TEMPLATED_DNA_BIOSYNTHETIC_PROCESS	1.32E-14
GOBP_POSITIVE_REGULATION_OF_TRANSPORT	1.45E-14
GOBP_CALCIUM_MEDIATED_SIGNALING	1.46E-14
GOBP_MYELOID_LEUKOCYTE_MEDIATED_IMMUNITY	1.47E-14
GOBP_GLYCOSYLATION	1.50E-14
GOBP_CELL_GROWTH	1.70E-14
GOBP_NEGATIVE_REGULATION_OF_CYTOKINE_PRODUCTION	1.76E-14
GOBP_SUPRAMOLECULAR_FIBER_ORGANIZATION	1.90E-14
GOBP_PROTEIN_LOCALIZATION_TO_EXTRACELLULAR_REGION	2.01E-14
GOBP_POSITIVE_REGULATION_OF_AMIDE_METABOLIC_PROCESS	2.03E-14
GOBP_NEGATIVE_REGULATION_OF_SUPRAMOLECULAR_FIBER_ORGANIZATION	2.20E-14
GOBP_REGULATION_OF_CATABOLIC_PROCESS	2.21E-14
GOBP_BIOLOGICAL_PROCESS_INVOLVED_IN_INTERACTION_WITH_HOST	2.49E-14
GOBP_REGULATION_OF_VIRAL_GENOME_REPLICATION	2.49E-14
GOBP_LIPID_LOCALIZATION	2.53E-14
GOBP_REGULATION_OF_PRODUCTION_OF_MOLECULAR_MEDIATOR_OF_IMMUNE_RESPONSE	2.53E-14
GOBP_IMPORT_INTO_CELL	2.75E-14
GOBP_RESPONSE_TO_HYDROGEN_PEROXIDE	2.77E-14
GOBP_PEPTIDE_TRANSPORT	2.86E-14
GOBP_CHAPERONE_MEDIATED_PROTEIN_FOLDING	2.89E-14
GOBP_REGULATION_OF_CYTOSKELETON_ORGANIZATION	3.17E-14
GOBP_PEPTIDYL_AMINO_ACID_MODIFICATION	3.23E-14
GOBP_VASCULATURE_DEVELOPMENT	3.29E-14
GOBP_REGULATION_OF_PROTEOLYSIS	3.41E-14
GOBP_NEGATIVE_REGULATION_OF_CATALYTIC_ACTIVITY	3.52E-14
GOBP_PROTEIN_STABILIZATION	3.59E-14
GOBP_SIGNAL_TRANSDUCTION_BY_P53_CLASS_MEDIATOR	3.67E-14
GOBP_RESPONSE_TO_TRANSFORMING_GROWTH_FACTOR_BETA	3.69E-14
GOBP_REGULATION_OF_SYNAPSE_STRUCTURE_OR_ACTIVITY	3.80E-14
GOBP_RESPONSE_TO_PEPTIDE_HORMONE	3.84E-14
GOBP_RAS_PROTEIN_SIGNAL_TRANSDUCTION	3.94E-14
GOBP_MACROAUTOPHAGY	3.95E-14
GOBP_POSITIVE_REGULATION_OF_HYDROLASE_ACTIVITY	3.97E-14
GOBP_INTRINSIC_APOPTOTIC_SIGNALING_PATHWAY	3.97E-14
GOBP_REGULATION_OF_PEPTIDE_TRANSPORT	4.02E-14
GOBP_REGULATION_OF_AUTOPHAGY	4.13E-14
GOBP_REGULATION_OF_CELLULAR_RESPONSE_TO_STRESS	4.44E-14
GOBP_POST_TRANSLATIONAL_PROTEIN_MODIFICATION	4.75E-14
GOBP_RESPONSE_TO_TUMOR_NECROSIS_FACTOR	4.96E-14
GOBP_REGULATION_OF_SYSTEM_PROCESS	5.52E-14
GOBP_POSITIVE_REGULATION_OF_CELLULAR_COMPONENT_BIOGENESIS	5.64E-14
GOBP_POST_TRANSCRIPTIONAL_REGULATION_OF_GENE_EXPRESSION	5.67E-14
GOBP_ACTIN_FILAMENT_BUNDLE_ORGANIZATION	6.43E-14
GOBP_RESPONSE_TO_TYPE_I_INTERFERON	6.47E-14
GOBP_IMMUNE_SYSTEM_DEVELOPMENT	6.57E-14
GOBP_CELLULAR_RESPONSE_TO_ORGANIC_CYCLIC_COMPOUND	6.99E-14
GOBP_TUBE_MORPHOGENESIS	7.03E-14
GOBP_NEGATIVE_REGULATION_OF_NEURON_APOPTOTIC_PROCESS	7.13E-14
GOBP_CELLULAR_COMPONENT_MORPHOGENESIS	7.30E-14
GOBP_INTRINSIC_APOPTOTIC_SIGNALING_PATHWAY_IN_RESPONSE_TO_DNA_DAMAGE	7.64E-14
GOBP_PROTEIN_COMPLEX_OLIGOMERIZATION	7.73E-14
GOBP_RESPONSE_TO_MECHANICAL_STIMULUS	8.27E-14
GOBP_CELLULAR_HOMEOSTASIS	8.57E-14
GOBP_NEGATIVE_REGULATION_OF_TRANSPORT	9.24E-14
GOBP_INTRACELLULAR_RECEPTOR_SIGNALING_PATHWAY	9.30E-14
GOBP_REGULATION_OF_INTRINSIC_APOPTOTIC_SIGNALING_PATHWAY	9.44E-14
GOBP_NEGATIVE_REGULATION_OF_MAPK_CASCADE	9.61E-14
GOBP_POSITIVE_REGULATION_OF_PHOSPHORUS_METABOLIC_PROCESS	9.69E-14
GOBP_POSITIVE_REGULATION_OF_CATABOLIC_PROCESS	1.05E-13
GOBP_CELLULAR_RESPONSE_TO_STEROID_HORMONE_STIMULUS	1.08E-13
GOBP_APOPTOTIC_MITOCHONDRIAL_CHANGES	1.10E-13

GOBP_POSITIVE_REGULATION_OF_CHEMOTAXIS	1.25E-13
GOBP_REGULATION_OF_SIGNAL_TRANSDUCTION_BY_P53_CLASS_MEDIATOR	1.31E-13
GOBP_ANATOMICAL_STRUCTURE_FORMATION_INVOLVED_IN_MORPHOGENESIS	1.33E-13
GOBP_REGULATION_OF_GTPASE_ACTIVITY	1.34E-13
GOBP_DEFENSE_RESPONSE_TO_SYMBIONT	1.36E-13
GOBP_STRIATED_MUSCLE_TISSUE_DEVELOPMENT	1.42E-13
GOBP_POSITIVE_REGULATION_OF_KINASE_ACTIVITY	1.43E-13
GOBP_CELL_PART_MORPHOGENESIS	1.58E-13
GOBP_BLOOD_VESSEL_MORPHOGENESIS	1.63E-13
GOBP_CALCIUM_ION_TRANSPORT	1.65E-13
GOBP_POSITIVE_REGULATION_OF_PROTEIN_CONTAINING_COMPLEX_ASSEMBLY	1.73E-13
GOBP_TISSUE_HOMEOSTASIS	1.74E-13
GOBP_CELLULAR_RESPONSE_TO_CHEMICAL_STRESS	1.95E-13
GOBP_NEGATIVE_REGULATION_OF_LOCOMOTION	2.00E-13
GOBP_PROTEASOMAL_PROTEIN_CATABOLIC_PROCESS	2.05E-13
GOBP_CELL_DIVISION	2.16E-13
GOBP_LIPID_MODIFICATION	2.18E-13
GOBP_REGULATION_OF_CELLULAR_RESPONSE_TO_GROWTH_FACTOR_STIMULUS	2.43E-13
GOBP_GOLGI_VESICLE_TRANSPORT	2.45E-13
GOBP_MUSCLE_TISSUE_DEVELOPMENT	2.53E-13
GOBP_REPRODUCTION	2.58E-13
GOBP_NEURON_DEVELOPMENT	2.62E-13
GOBP_POSITIVE_REGULATION_OF_ENDOCYTOSIS	2.63E-13
GOBP_REGULATION_OF_RESPONSE_TO_BIOTIC_STIMULUS	2.65E-13
GOBP_DNA_BIOSYNTHETIC_PROCESS	2.68E-13
GOBP_REGULATION_OF_LYMPHOCYTE_MEDIATED_IMMUNITY	2.93E-13
GOBP_EXOCYTOSIS	2.97E-13
GOBP_NEGATIVE_REGULATION_OF_PHOSPHORUS_METABOLIC_PROCESS	2.99E-13
GOBP_CELLULAR_RESPONSE_TO_PEPTIDE	3.00E-13
GOBP_SIGNAL_RELEASE	3.01E-13
GOBP_DNA_REPLICATION	3.04E-13
GOBP_DEVELOPMENTAL_PROCESS_INVOLVED_IN_REPRODUCTION	3.12E-13
GOBP_OSSIFICATION	3.32E-13
GOBP_REGULATION_OF_CELL_GROWTH	3.41E-13
GOBP_NEGATIVE_REGULATION_OF_PROTEOLYSIS	3.44E-13
GOBP_UBIQUITIN_DEPENDENT_ERAD_PATHWAY	3.73E-13
GOBP_CELLULAR_RESPONSE_TO_ABIOTIC_STIMULUS	3.85E-13
GOBP_NEGATIVE_REGULATION_OF_DEFENSE_RESPONSE	4.23E-13
GOBP_PROTEIN_PROCESSING	4.72E-13
GOBP_DEFENSE_RESPONSE_TO_BACTERIUM	4.77E-13
GOBP_RESPONSE_TO_OXYGEN_LEVELS	4.83E-13
GOBP_RESPONSE_TO_KETONE	4.88E-13
GOBP_MONOCARBOXYLIC_ACID_METABOLIC_PROCESS	5.04E-13
GOBP_SECOND_MESSENGER_MEDIATED_SIGNALING	5.40E-13
GOBP_CELL_CYCLE_PROCESS	5.66E-13
GOBP_MICROTUBULE_CYTOSKELETON_ORGANIZATION	5.70E-13
GOBP_PROTEIN_LOCALIZATION_TO_CHROMOSOME	5.83E-13
GOBP_GLAND_DEVELOPMENT	5.86E-13
GOBP_POSITIVE_REGULATION_OF_PHOSPHORYLATION	6.05E-13
GOBP_POSITIVE_REGULATION_OF_CYTOSKELETON_ORGANIZATION	6.21E-13
GOBP_FAT_CELL_DIFFERENTIATION	6.27E-13
GOBP_TRANSMEMBRANE_RECEPTOR_PROTEIN_TYROSINE_KINASE_SIGNALING_PATHWAY	6.48E-13
GOBP_CELLULAR_RESPONSE_TO_PEPTIDE_HORMONE_STIMULUS	6.69E-13
GOBP_POSITIVE_REGULATION_OF_PEPTIDYL_TYROSINE_PHOSPHORYLATION	7.29E-13
GOBP_CELLULAR_COMPONENT_DISASSEMBLY	7.41E-13
GOBP_RESPONSE_TO_WOUNDING	8.27E-13
GOBP_BONE_DEVELOPMENT	8.29E-13
GOBP_PROTEIN_CONTAINING_COMPLEX_DISASSEMBLY	9.24E-13
GOBP_REGULATION_OF_INTRACELLULAR_TRANSPORT	9.99E-13
GOBP_MULTICELLULAR_ORGANISM_REPRODUCTION	1.06E-12
GOBP_EMBRYONIC_ORGAN_MORPHOGENESIS	1.08E-12
GOBP_REGULATION_OF_CELLULAR_CATABOLIC_PROCESS	1.11E-12
GOBP_CYTOKINE_PRODUCTION_INVOLVED_IN_IMMUNE_RESPONSE	1.22E-12
GOBP_REGULATION_OF_PEPTIDYL_TYROSINE_PHOSPHORYLATION	1.25E-12

GOBP_NEGATIVE_REGULATION_OF_HYDROLASE_ACTIVITY	1.27E-12
GOBP_NEGATIVE_REGULATION_OF_PROTEIN_MODIFICATION_PROCESS	1.29E-12
GOBP_NEGATIVE_REGULATION_OF_PEPTIDASE_ACTIVITY	1.29E-12
GOBP_HETEROCYCLE_CATABOLIC_PROCESS	1.45E-12
GOBP_POSITIVE_REGULATION_OF_BINDING	1.50E-12
GOBP_NEGATIVE_REGULATION_OF_AUTOPHAGY	1.52E-12
GOBP_AMEBOIDAL_TYPE_CELL_MIGRATION	1.55E-12
GOBP_VIRAL_GENOME_REPLICATION	1.62E-12
GOBP_ACTOMYOSIN_STRUCTURE_ORGANIZATION	1.94E-12
GOBP_POSITIVE_REGULATION_OF_ORGANELLE_ORGANIZATION	1.94E-12
GOBP_SMALL_MOLECULE_BIOSYNTHETIC_PROCESS	2.06E-12
GOBP_ORGANIC_CYCLIC_COMPOUND_CATABOLIC_PROCESS	2.11E-12
GOBP_POSITIVE_REGULATION_OF_CELL_SUBSTRATE_ADHESION	2.20E-12
GOBP_LIPID_BIOSYNTHETIC_PROCESS	2.32E-12
GOBP_POSITIVE_REGULATION_OF_CELL_GROWTH	2.32E-12
GOBP_GOLGI_ORGANIZATION	2.38E-12
GOBP_VESICLE_ORGANIZATION	2.44E-12
GOBP_POSITIVE_REGULATION_OF_SECRETION	2.65E-12
GOBP_REGULATION_OF_PEPTIDASE_ACTIVITY	2.67E-12
GOBP_TELOMERE_ORGANIZATION	2.76E-12
GOBP_ENDOPLASMIC_RETICULUM_TO_GOLGI_VESICLE_MEDIATED_TRANSPORT	2.84E-12
GOBP_NON_MEMBRANE_BOUNDED_ORGANELLE_ASSEMBLY	3.00E-12
GOBP_RESPONSE_TO_REACTIVE_OXYGEN_SPECIES	3.02E-12
GOBP_POSITIVE_REGULATION_OF_CELL_CYCLE_PROCESS	3.02E-12
GOBP_PROTEIN_MATURATION	3.03E-12
GOBP_MACROPHAGE_ACTIVATION	3.04E-12
GOBP_EMBRYONIC_MORPHOGENESIS	3.27E-12
GOBP_LYSOSOMAL_TRANSPORT	3.28E-12
GOBP_G_PROTEIN_COUPLED_RECEPTOR_SIGNALING_PATHWAY	3.30E-12
GOBP_PROTEIN_FOLDING	3.30E-12
GOBP_ENDOSOMAL_TRANSPORT	3.52E-12
GOBP_POSITIVE_REGULATION_OF_PROTEIN_KINASE_ACTIVITY	3.56E-12
GOBP_TUBE_DEVELOPMENT	3.75E-12
GOBP_CELLULAR_RESPONSE_TO_OXYGEN_LEVELS	3.79E-12
GOBP_NEGATIVE_REGULATION_OF_CELL_CELL_ADHESION	3.93E-12
GOBP_POSITIVE_REGULATION_OF_PROTEIN_LOCALIZATION	4.38E-12
GOBP_EMBRYONIC_ORGAN_DEVELOPMENT	4.46E-12
GOBP_REGULATORY_NCRNA_MEDIATED_GENE_SILENCING	4.50E-12
GOBP_PROTEIN_POLYMERIZATION	5.07E-12
GOBP_GLIOGENESIS	5.65E-12
GOBP_REGULATION_OF_MICROTUBULE_CYTOSKELETON_ORGANIZATION	5.74E-12
GOBP_REGULATION_OF_ADAPTIVE_IMMUNE_RESPONSE	5.75E-12
GOBP_POSITIVE_REGULATION_OF_INFLAMMATORY_RESPONSE	7.04E-12
GOBP_RESPONSE_TO_ALCOHOL	7.16E-12
GOBP_ADAPTIVE_THERMOGENESIS	7.44E-12
GOBP_REGULATION_OF_METAL_ION_TRANSPORT	7.57E-12
GOBP_FATTY_ACID_METABOLIC_PROCESS	7.65E-12
GOBP_CELLULAR_RESPONSE_TO_NITROGEN_COMPOUND	7.65E-12
GOBP_HEMATOPOIETIC_OR_LYMPHOID_ORGAN_DEVELOPMENT	7.81E-12
GOBP_ENDOCYTOSIS	8.21E-12
GOBP_RESPONSE_TO_INSULIN	8.35E-12
GOBP_REGULATION_OF_CELL_CYCLE_G1_S_PHASE_TRANSITION	8.39E-12
GOBP_RECEPTOR_MEDIATED_ENDOCYTOSIS	8.89E-12
GOBP_ORGANELLE_ASSEMBLY	9.10E-12
GOBP_MITOTIC_CELL_CYCLE	9.22E-12
GOBP_REGULATION_OF_INTRACELLULAR_PROTEIN_TRANSPORT	9.60E-12
GOBP_TRANSMEMBRANE_RECEPTOR_PROTEIN_SERINE_THREONINE_KINASE_SIGNALING_PATHWAY	9.61E-12
GOBP_ERAD_PATHWAY	9.86E-12
GOBP_POSITIVE_REGULATION_OF_CELL_CYCLE_PHASE_TRANSITION	1.01E-11
GOBP_REGULATION_OF_PROTEIN_POLYMERIZATION	1.07E-11
GOBP_STEROID_HORMONE_MEDIATED_SIGNALING_PATHWAY	1.08E-11
GOBP_SYNAPTIC_SIGNALING	1.09E-11
GOBP_PEPTIDYL_SERINE_MODIFICATION	1.11E-11
GOBP_ORGANIC_HYDROXY_COMPOUND_METABOLIC_PROCESS	1.12E-11

GOBP_MUSCLE_SYSTEM_PROCESS	1.20E-11
GOBP_REGULATION_OF_BINDING	1.24E-11
GOBP_NEGATIVE_REGULATION_OF_TRANSFERASE_ACTIVITY	1.31E-11
GOBP_POSITIVE_REGULATION_OF_DNA_METABOLIC_PROCESS	1.36E-11
GOBP_REGULATION_OF_MONOATOMIC_ION_TRANSPORT	1.37E-11
GOBP_MITOTIC_CELL_CYCLE_PROCESS	1.37E-11
GOBP_REGULATION_OF_BODY_FLUID_LEVELS	1.46E-11
GOBP_MICROTUBULE_BASED_PROCESS	1.47E-11
GOBP_PROTEIN_DNA_COMPLEX_ASSEMBLY	1.48E-11
GOBP_MRNA_CATABOLIC_PROCESS	1.49E-11
GOBP_REGULATION_OF_VESICLE_MEDIATED_TRANSPORT	1.52E-11
GOBP_REGULATION_OF_NERVOUS_SYSTEM_DEVELOPMENT	1.58E-11
GOBP_HORMONE_TRANSPORT	1.61E-11
GOBP_REGULATION_OF_SMALL_GTPASE_MEDIATED_SIGNAL_TRANSDUCTION	1.62E-11
GOBP_REGULATION_OF_CELL_CYCLE_PROCESS	1.66E-11
GOBP_REGULATION_OF_DNA_METABOLIC_PROCESS	1.76E-11
GOBP_REGULATION_OF_SMALL_MOLECULE_METABOLIC_PROCESS	1.76E-11
GOBP_POSITIVE_REGULATION_OF_PRODUCTION_OF_MOLECULAR_MEDIATOR_OF_IMMUNE_RESPONSE	1.77E-11
GOBP_REGULATION_OF_ESTABLISHMENT_OF_PROTEIN_LOCALIZATION	1.89E-11
GOBP_REGULATION_OF_CELL_SUBSTRATE_ADHESION	2.07E-11
GOBP_EMBRYO_DEVELOPMENT	2.15E-11
GOBP_POSITIVE_REGULATION_OF_CELLULAR_CATABOLIC_PROCESS	2.32E-11
GOBP_REGULATION_OF_CELL_PROJECTION_ORGANIZATION	2.42E-11
GOBP_DEVELOPMENTAL_GROWTH_INVOLVED_IN_MORPHOGENESIS	2.49E-11
GOBP_DNA_DAMAGE_RESPONSE	2.57E-11
GOBP_REGULATION_OF_PROTEIN_SECRETION	2.65E-11
GOBP_NEGATIVE_REGULATION_OF_APOPTOTIC_SIGNALING_PATHWAY	2.66E-11
GOBP_MITOTIC_SISTER_CHROMATID_SEGREGATION	2.73E-11
GOBP_RNA_PROCESSING	2.84E-11
GOBP_SISTER_CHROMATID_SEGREGATION	3.01E-11
GOBP_B_CELL_ACTIVATION_INVOLVED_IN_IMMUNE_RESPONSE	3.13E-11
GOBP_PLACENTA_DEVELOPMENT	3.36E-11
GOBP_REGULATION_OF_MITOCHONDRION_ORGANIZATION	3.49E-11
GOBP_ORGANIC_ACID_BIOSYNTHETIC_PROCESS	4.01E-11
GOBP_POSITIVE_REGULATION_OF_CELL_CYCLE	4.03E-11
GOBP_REGULATION_OF_TRANSLATIONAL_INITIATION	4.07E-11
GOBP_VIRAL_GENE_EXPRESSION	4.09E-11
GOBP_MUSCLE_ORGAN_DEVELOPMENT	4.30E-11
GOBP_ELECTRON_TRANSPORT_CHAIN	4.32E-11
GOBP_STEM_CELL_DIFFERENTIATION	4.41E-11
GOBP_NEGATIVE_REGULATION_OF_PHOSPHORYLATION	4.41E-11
GOBP_REGULATION_OF_CELL_DIVISION	4.85E-11
GOBP_REGULATION_OF_NEURON_PROJECTION_DEVELOPMENT	4.86E-11
GOBP_REGULATION_OF_PROTEIN_LOCALIZATION_TO_NUCLEUS	5.00E-11
GOBP_NEGATIVE_REGULATION_OF_CYTOSKELETON_ORGANIZATION	5.01E-11
GOBP_GLIAL_CELL_DIFFERENTIATION	5.46E-11
GOBP_ORGANIC_ANION_TRANSPORT	5.52E-11
GOBP_HEAD_DEVELOPMENT	5.71E-11
GOBP_REGULATION_OF_CELL_CYCLE_PHASE_TRANSITION	6.21E-11
GOBP_REGULATION_OF_VASCULATURE_DEVELOPMENT	6.32E-11
GOBP_CHROMOSOME_SEGREGATION	6.46E-11
GOBP_CELLULAR_RESPONSE_TO_UNFOLDED_PROTEIN	6.89E-11
GOBP_RESPONSE_TO_TOXIC_SUBSTANCE	7.23E-11
GOBP_MIRNA_METABOLIC_PROCESS	8.21E-11
GOBP_PATTERN_SPECIFICATION_PROCESS	8.28E-11
GOBP_REGULATION_OF_PROTEIN_BINDING	8.58E-11
GOBP_REGULATION_OF_EPITHELIAL_CELL_PROLIFERATION	8.59E-11
GOBP_CARBOHYDRATE_HOMEOSTASIS	8.71E-11
GOBP_CELLULAR_RESPONSE_TO_INSULIN_STIMULUS	9.00E-11
GOBP_REGULATION_OF_HORMONE_SECRETION	9.66E-11
GOBP_REGULATION_OF_CALCIUM_ION_TRANSPORT	9.70E-11
GOBP_PROTEIN_LOCALIZATION_TO_VACUOLE	9.70E-11
GOBP_ESTABLISHMENT_OF_ORGANELLE_LOCALIZATION	1.00E-10
GOBP_ENDOPLASMIC_RETICULUM_UNFOLDED_PROTEIN_RESPONSE	1.01E-10

GOBP_REGULATION_OF_MICROTUBULE_BASED_PROCESS	1.01E-10
GOBP_PROTEIN_MODIFICATION_BY_SMALL_PROTEIN_CONJUGATION	1.07E-10
GOBP_MONOCARBOXYLIC_ACID_BIOSYNTHETIC_PROCESS	1.09E-10
GOBP_CELLULAR_RESPONSE_TO_TYPE_II_INTERFERON	1.13E-10
GOBP_NEGATIVE_REGULATION_OF_ESTABLISHMENT_OF_PROTEIN_LOCALIZATION	1.22E-10
GOBP_MITOCHONDRIAL_MEMBRANE_ORGANIZATION	1.25E-10
GOBP_HEMATOPOIETIC_PROGENITOR_CELL_DIFFERENTIATION	1.30E-10
GOBP_DNA_TEMPLATED_DNA_REPLICATION	1.51E-10
GOBP_EPITHELIUM_DEVELOPMENT	1.53E-10
GOBP_NEGATIVE_REGULATION_OF_CATABOLIC_PROCESS	1.57E-10
GOBP_HEART_DEVELOPMENT	1.63E-10
GOBP_PROTEIN_RNA_COMPLEX_ORGANIZATION	1.65E-10
GOBP_POSITIVE_REGULATION_OF_RESPONSE_TO_BIOTIC_STIMULUS	1.76E-10
GOBP_NEGATIVE_REGULATION_OF_CELL_CYCLE	1.77E-10
GOBP_CENTRAL_NERVOUS_SYSTEM_DEVELOPMENT	1.97E-10
GOBP_REGULATION_OF_GENERATION_OF_PRECURSOR_METABOLITES_AND_ENERGY	2.16E-10
GOBP_NERVOUS_SYSTEM_PROCESS	2.18E-10
GOBP_NUCLEAR_CHROMOSOME_SEGREGATION	2.32E-10
GOBP_PHOSPHATIDYLINOSITOL_3_KINASE_PROTEIN_KINASE_B_SIGNAL_TRANSDUCTION	2.38E-10
GOBP_GLYCEROLIPID_BIOSYNTHETIC_PROCESS	2.39E-10
GOBP_SMALL_MOLECULE_CATABOLIC_PROCESS	2.40E-10
GOBP_VACUOLAR_TRANSPORT	2.41E-10
GOBP_LEUKOCYTE_APOPTOTIC_PROCESS	2.62E-10
GOBP_MITOCHONDRIAL_TRANSPORT	2.97E-10
GOBP_RNA_CATABOLIC_PROCESS	3.65E-10
GOBP_REGULATION_OF_RESPONSE_TO_CYTOKINE_STIMULUS	3.77E-10
GOBP_RESPIRATORY_ELECTRON_TRANSPORT_CHAIN	3.88E-10
GOBP_NEURAL_PRECURSOR_CELL_PROLIFERATION	3.89E-10
GOBP_NEGATIVE_REGULATION_OF_GROWTH	3.91E-10
GOBP_NUCLEUS_ORGANIZATION	3.94E-10
GOBP_CYTOKINESIS	4.35E-10
GOBP_TISSUE_MIGRATION	4.36E-10
GOBP_TUMOR_NECROSIS_FACTOR_MEDIATED_SIGNALING_PATHWAY	4.87E-10
GOBP_REGULATION_OF_MRNA_METABOLIC_PROCESS	4.96E-10
GOBP_SPINDLE_ASSEMBLY	5.23E-10
GOBP_ATP_SYNTHESIS_COUPLED_ELECTRON_TRANSPORT	5.59E-10
GOBP_CELL_CYCLE_PHASE_TRANSITION	5.90E-10
GOBP_NEGATIVE_REGULATION_OF_BINDING	6.02E-10
GOBP_EMBRYO_DEVELOPMENT_ENDING_IN_BIRTH_OR_EGG_HATCHING	6.35E-10
GOBP_RESPONSE_TO_TEMPERATURE_STIMULUS	7.51E-10
GOBP_CONNECTIVE_TISSUE_DEVELOPMENT	8.08E-10
GOBP_PROTEIN_LOCALIZATION_TO_NUCLEUS	8.84E-10
GOBP_ORGANELLE_LOCALIZATION	9.30E-10
GOBP_TRANSFORMING_GROWTH_FACTOR_BETA_RECEPTOR_SIGNALING_PATHWAY	9.39E-10
GOBP_TEMPERATURE_HOMEOSTASIS	9.44E-10
GOBP_RESPONSE_TO_MONOSACCHARIDE	1.01E-09
GOBP_REGULATION_OF_CYSTEINE_TYPE_ENDOPEPTIDASE_ACTIVITY	1.02E-09
GOBP_SELECTIVE_AUTOPHAGY	1.05E-09
GOBP_REGULATION_OF_PROTEOLYSIS_INVOLVED_IN_PROTEIN_CATABOLIC_PROCESS	1.09E-09
GOBP_CALCIUM_ION_TRANSMEMBRANE_TRANSPORT	1.10E-09
GOBP_CELLULAR_KETONE_METABOLIC_PROCESS	1.10E-09
GOBP_CELL_SUBSTRATE_ADHESION	1.11E-09
GOBP_NEGATIVE_REGULATION_OF_CELL_PROJECTION_ORGANIZATION	1.12E-09
GOBP_CYTOSOLIC_TRANSPORT	1.13E-09
GOBP_RESPONSE_TO_HEAT	1.15E-09
GOBP_POSITIVE_REGULATION_OF_CHROMOSOME_ORGANIZATION	1.18E-09
GOBP_MITOTIC_CELL_CYCLE_PHASE_TRANSITION	1.27E-09
GOBP_EPIGENETIC_REGULATION_OF_GENE_EXPRESSION	1.28E-09
GOBP_EPITHELIAL_CELL_DEVELOPMENT	1.30E-09
GOBP_POSITIVE_REGULATION_OF_GTPASE_ACTIVITY	1.33E-09
GOBP_POSITIVE_REGULATION_OF_NEURON_PROJECTION_DEVELOPMENT	1.34E-09
GOBP_POSITIVE_REGULATION_OF_AUTOPHAGY	1.36E-09
GOBP_REGULATION_OF_NEUROGENESIS	1.39E-09
GOBP_ORGANELLE_MEMBRANE_FUSION	1.39E-09

GOBP_REGULATION_OF_UBIQUITIN_DEPENDENT_PROTEIN_CATABOLIC_PROCESS	1.46E-09
GOBP_PROTEIN_HOMOOLIGOMERIZATION	1.49E-09
GOBP_MITOTIC_SPINDLE_ORGANIZATION	1.55E-09
GOBP_CANONICAL_WNT_SIGNALING_PATHWAY	1.69E-09
GOBP_RNA_MODIFICATION	1.86E-09
GOBP_HORMONE_MEDIATED_SIGNALING_PATHWAY	1.87E-09
GOBP_REGULATION_OF_DNA_REPLICATION	2.02E-09
GOBP_NEGATIVE_REGULATION_OF_MITOTIC_CELL_CYCLE_PHASE_TRANSITION	2.09E-09
GOBP_NUCLEOSOME_ORGANIZATION	2.10E-09
GOBP_MRNA_METABOLIC_PROCESS	2.14E-09
GOBP_REGULATION_OF_INNATE_IMMUNE_RESPONSE	2.22E-09
GOBP_CELLULAR_RESPONSE_TO_REACTIVE_OXYGEN_SPECIES	2.26E-09
GOBP_REGULATION_OF_MONONUCLEAR_CELL_MIGRATION	2.26E-09
GOBP_REGULATION_OF_CHROMOSOME_ORGANIZATION	2.32E-09
GOBP_CELL_JUNCTION_ASSEMBLY	2.34E-09
GOBP_REGULATION_OF_CANONICAL_WNT_SIGNALING_PATHWAY	2.45E-09
GOBP_POSITIVE_REGULATION_OF_PHOSPHATIDYLINOSITOL_3_KINASE_PROTEIN_KINASE_B_SIGNAL_TRANSDUCTION	2.45E-09
GOBP_POSITIVE_REGULATION_OF_PROTEOLYSIS	2.51E-09
GOBP_CARBOHYDRATE_METABOLIC_PROCESS	2.53E-09
GOBP_REGULATION_OF_ENDOCYTOSIS	2.55E-09
GOBP_VESICLE_LOCALIZATION	2.58E-09
GOBP_NEGATIVE_REGULATION_OF_VIRAL_PROCESS	2.58E-09
GOBP_IN_UTERO_EMBRYONIC_DEVELOPMENT	2.75E-09
GOBP_SPINDLE_ORGANIZATION	2.80E-09
GOBP_INTERFERON_MEDIATED_SIGNALING_PATHWAY	2.85E-09
GOBP_MITOCHONDRIAL_GENE_EXPRESSION	2.96E-09
GOBP_POSITIVE_REGULATION_OF_EPITHELIAL_CELL_MIGRATION	3.01E-09
GOBP_ENDOPLASMIC_RETICULUM_ORGANIZATION	3.10E-09
GOBP_CELLULAR_RESPONSE_TO_STARVATION	3.13E-09
GOBP_NEGATIVE_REGULATION_OF_CELLULAR_CATABOLIC_PROCESS	3.21E-09
GOBP_REGULATION_OF_PROTEIN_CATABOLIC_PROCESS	3.69E-09
GOBP_CELL_CYCLE_G1_S_PHASE_TRANSITION	3.70E-09
GOBP_REGULATION_OF_MITOTIC_CELL_CYCLE_PHASE_TRANSITION	3.72E-09
GOBP_REGULATION_OF_CELL_MORPHOGENESIS	3.75E-09
GOBP_MEMBRANE_FUSION	3.86E-09
GOBP_SEXUAL_REPRODUCTION	3.88E-09
GOBP_NEGATIVE_REGULATION_OF_TRANSMEMBRANE_RECEPTOR_PROTEIN_SERINE_THREONINE_KINASE_SIGNALING_PATHW/	3.90E-09
GOBP_NEGATIVE_REGULATION_OF_RESPONSE_TO_BIOTIC_STIMULUS	4.00E-09
GOBP_REGULATION_OF_MITOTIC_CELL_CYCLE	4.25E-09
GOBP_DNA_RECOMBINATION	4.78E-09
GOBP_REGULATION_OF_MRNA_CATABOLIC_PROCESS	5.15E-09
GOBP_POSITIVE_REGULATION_OF_CANONICAL_WNT_SIGNALING_PATHWAY	5.22E-09
GOBP_NON_CANONICAL_NF_KAPPAB_SIGNAL_TRANSDUCTION	5.62E-09
GOBP_RESPONSE_TO_NUTRIENT	6.34E-09
GOBP_PHOSPHOLIPID_BIOSYNTHETIC_PROCESS	7.01E-09
GOBP_REGULATION_OF_CHROMOSOME_SEGREGATION	7.73E-09
GOBP_GLYCEROPHOSPHOLIPID_BIOSYNTHETIC_PROCESS	8.11E-09
GOBP_TELOMERE_MAINTENANCE	8.52E-09
GOBP_CELL_MORPHOGENESIS_INVOLVED_IN_NEURON_DIFFERENTIATION	8.57E-09
GOBP_LIPID_CATABOLIC_PROCESS	9.22E-09
GOBP_CYTOSKELETON_DEPENDENT_CYTOKINESIS	9.53E-09
GOBP_REGULATION_OF_PROTEASOMAL_PROTEIN_CATABOLIC_PROCESS	9.77E-09
GOBP_COGNITION	1.06E-08
GOBP_POSITIVE_REGULATION_OF_MAPK_CASCADE	1.19E-08
GOBP_RNA_LOCALIZATION	1.20E-08
GOBP_POSITIVE_REGULATION_OF_WNT_SIGNALING_PATHWAY	1.34E-08
GOBP_RESPONSE_TO_STARVATION	1.51E-08
GOBP_MITOCHONDRIAL_TRANSLATION	1.52E-08
GOBP_MICROTUBULE_ORGANIZING_CENTER_ORGANIZATION	1.62E-08
GOBP_REGULATION_OF_WNT_SIGNALING_PATHWAY	2.00E-08
GOBP_CARBOHYDRATE_DERIVATIVE_METABOLIC_PROCESS	2.02E-08
GOBP_NEURAL_TUBE_DEVELOPMENT	2.08E-08
GOBP_RESPONSE_TO_CARBOHYDRATE	2.25E-08
GOBP_EPITHELIAL_CELL_DIFFERENTIATION	2.26E-08

GOBP_REGULATION_OF_TRANSMEMBRANE_RECEPTOR_PROTEIN_SERINE_THREONINE_KINASE_SIGNALING_PATHWAY	2.26E-08
GOBP_NEGATIVE_REGULATION_OF_DNA_METABOLIC_PROCESS	2.47E-08
GOBP_INSULIN_RECEPTOR_SIGNALING_PATHWAY	2.50E-08
GOBP_NEGATIVE_REGULATION_OF_LEUKOCYTE_CELL_CELL_ADHESION	2.53E-08
GOBP_HEMOSTASIS	2.54E-08
GOBP_POSITIVE_REGULATION_OF_PROTEIN_LOCALIZATION_TO_MEMBRANE	2.61E-08
GOBP_REGULATION_OF_TELOMERE_MAINTENANCE	2.66E-08
GOBP_EXTRINSIC_APOPTOTIC_SIGNALING_PATHWAY	2.68E-08
GOBP_RETROGRADE_TRANSPORT_ENDOSOME_TO_GOLGI	2.96E-08
GOBP_MICROTUBULE_CYTOSKELETON_ORGANIZATION_INVOLVED_IN_MITOSIS	2.99E-08
GOBP_LYTIC_VACUOLE_ORGANIZATION	3.14E-08
GOBP_NEGATIVE_REGULATION_OF_INNATE_IMMUNE_RESPONSE	3.26E-08
GOBP_GAMETE_GENERATION	3.39E-08
GOBP_DEVELOPMENT_OF_PRIMARY_SEXUAL_CHARACTERISTICS	3.57E-08
GOBP_NUCLEAR_TRANSCRIBED_MRNA_CATABOLIC_PROCESS	3.65E-08
GOBP_HEPATICOBILIARY_SYSTEM_DEVELOPMENT	3.68E-08
GOBP_POSITIVE_REGULATION_OF_TRANSLATION	4.00E-08
GOBP_REGULATION_OF_CALCIUM_ION_TRANSMEMBRANE_TRANSPORT	4.01E-08
GOBP_MAINTENANCE_OF_PROTEIN_LOCATION	4.21E-08
GOBP_REGULATION_OF_TRANSMEMBRANE_TRANSPORT	4.37E-08
GOBP_METHYLATION	4.39E-08
GOBP_SMALL_MOLECULE_METABOLIC_PROCESS	4.61E-08
GOBP_ACTIVATION_OF_INNATE_IMMUNE_RESPONSE	4.65E-08
GOBP_NEGATIVE_REGULATION_OF_INTRINSIC_APOPTOTIC_SIGNALING_PATHWAY	4.70E-08
GOBP_BEHAVIOR	4.74E-08
GOBP_EPITHELIAL_CELL_PROLIFERATION	4.97E-08
GOBP_NEGATIVE_REGULATION_OF_CELL_GROWTH	5.23E-08
GOBP_VESICLE_BUDDING_FROM_MEMBRANE	5.35E-08
GOBP_NEGATIVE_REGULATION_OF_CELL_CYCLE_PROCESS	5.36E-08
GOBP_REGULATION_OF_DEPHOSPHORYLATION	5.42E-08
GOBP_REGULATION_OF_MRNA_SPLICING_VIA_SPLICEOSOME	5.82E-08
GOBP_REGULATION_OF_PROTEASOMAL_UBIQUITIN_DEPENDENT_PROTEIN_CATABOLIC_PROCESS	6.76E-08
GOBP_POSITIVE_REGULATION_OF_CELL_PROJECTION_ORGANIZATION	7.02E-08
GOBP_POSITIVE_REGULATION_OF_PROTEIN_CATABOLIC_PROCESS	7.29E-08
GOBP_POSITIVE_REGULATION_OF_PROTEOLYSIS_INVOLVED_IN_PROTEIN_CATABOLIC_PROCESS	7.57E-08
GOBP_PHAGOCYTOSIS	8.15E-08
GOBP_POSITIVE_REGULATION_OF_UBIQUITIN_DEPENDENT_PROTEIN_CATABOLIC_PROCESS	8.45E-08
GOBP_MACROMOLECULE_METHYLATION	9.51E-08
GOBP_REGULATION_OF_CELLULAR_RESPONSE_TO_TRANSFORMING_GROWTH_FACTOR_BETA_STIMULUS	1.07E-07
GOBP_REGULATION_OF_TRANSPORTER_ACTIVITY	1.38E-07
GOBP_NEGATIVE_REGULATION_OF_DNA_BINDING_TRANSCRIPTION_FACTOR_ACTIVITY	1.40E-07
GOBP_PROTEIN_AUTOPHOSPHORYLATION	1.43E-07
GOBP_REGULATION_OF_EPITHELIAL_CELL_MIGRATION	1.71E-07
GOBP_MITOTIC_NUCLEAR_DIVISION	1.97E-07
GOBP_STRESS_FIBER_ASSEMBLY	2.02E-07
GOBP_PHOSPHOLIPID_METABOLIC_PROCESS	2.10E-07
GOBP_ORGANIC_HYDROXY_COMPOUND_BIOSYNTHETIC_PROCESS	2.20E-07
GOBP_FOREBRAIN_DEVELOPMENT	2.21E-07
GOBP_REGULATION_OF_ORGANELLE_ASSEMBLY	2.31E-07
GOBP_POSITIVE_REGULATION_OF_PEPTIDASE_ACTIVITY	2.41E-07
GOBP_CELLULAR_SENESCENCE	2.58E-07
GOBP_POSITIVE_REGULATION_OF_PROTEASOMAL_PROTEIN_CATABOLIC_PROCESS	2.94E-07
GOBP_POSTSYNAPSE_ORGANIZATION	2.97E-07
GOBP_NEGATIVE_REGULATION_OF_MRNA_METABOLIC_PROCESS	3.01E-07
GOBP_POSITIVE_REGULATION_OF_CYSTEINE_TYPE_ENDOPEPTIDASE_ACTIVITY	3.35E-07
GOBP_MUSCLE_CONTRACTION	3.41E-07
GOBP_ALCOHOL_METABOLIC_PROCESS	3.42E-07
GOBP_REGULATION_OF_EXTRINSIC_APOPTOTIC_SIGNALING_PATHWAY	3.54E-07
GOBP_NUCLEAR_TRANSPORT	3.82E-07
GOBP_BIOLOGICAL_PROCESS_INVOLVED_IN_INTERACTION_WITH_SYMBIONT	4.01E-07
GOBP_REGULATION_OF_RECEPTOR_MEDIATED_ENDOCYTOSIS	4.03E-07
GOBP_REGULATION_OF_MONOATOMIC_CATION_TRANSMEMBRANE_TRANSPORT	4.26E-07
GOBP_REGULATION_OF_MONOATOMIC_ION_TRANSMEMBRANE_TRANSPORT	4.41E-07
GOBP_SENSORY_ORGAN_DEVELOPMENT	4.42E-07

GOBP_MITOTIC_CYTOKINESIS	4.45E-07
GOBP_REGULATION_OF_NON_CANONICAL_NF_KAPPAB_SIGNAL_TRANSDUCTION	4.55E-07
GOBP_REGULATION_OF_PROTEIN_UBIQUITINATION	4.71E-07
GOBP_GLYCEROLIPID_METABOLIC_PROCESS	4.71E-07
GOBP_REGULATION_OF_MUSCLE_CELL_DIFFERENTIATION	4.89E-07
GOBP_SENSORY_SYSTEM_DEVELOPMENT	4.92E-07
GOBP_ORGANIC_ACID_METABOLIC_PROCESS	5.45E-07
GOBP_PROTEIN_LOCALIZATION_TO_MITOCHONDRION	5.71E-07
GOBP_CARBOHYDRATE_BIOSYNTHETIC_PROCESS	5.82E-07
GOBP_POSITIVE_REGULATION_OF_PLASMA_MEMBRANE_BOUNDED_CELL_PROJECTION_ASSEMBLY	5.84E-07
GOBP_REGULATION_OF_SISTER_CHROMATID_SEGREGATION	5.94E-07
GOBP_NEGATIVE_REGULATION_OF_MITOTIC_CELL_CYCLE	6.31E-07
GOBP_RNA_SPLICING_VIA_TRANSESTERIFICATION_REACTIONS	6.40E-07
GOBP_TRANSLATIONAL_INITIATION	6.83E-07
GOBP_ORGANIC_ACID_TRANSPORT	7.40E-07
GOBP_SPLICEOSOMAL_COMPLEX_ASSEMBLY	7.40E-07
GOBP_HEART_PROCESS	7.53E-07
GOBP_REGULATION_OF_POST_TRANSLATIONAL_PROTEIN_MODIFICATION	8.50E-07
GOBP_CARBOHYDRATE_DERIVATIVE_BIOSYNTHETIC_PROCESS	8.64E-07
GOBP_RECEPTOR_SIGNALING_PATHWAY_VIA_STAT	9.57E-07
GOBP_REGULATION_OF_VIRAL_INDUCED_CYTOPLASMIC_PATTERN_RECOGNITION_RECEPTOR_SIGNALING_PATHWAY	1.03E-06
GOBP_NCRNA_TRANSCRIPTION	1.03E-06
GOBP_IMPORT_ACROSS_PLASMA_MEMBRANE	1.07E-06
GOBP_GENERATION_OF_PRECURSOR_METABOLITES_AND_ENERGY	1.14E-06
GOBP_RNA_DESTABILIZATION	1.21E-06
GOBP_CELLULAR_RESPONSE_TO_TOPOLOGICALLY_INCORRECT_PROTEIN	1.22E-06
GOBP_DNA_TEMPLATED_TRANSCRIPTION_ELONGATION	1.24E-06
GOBP_REGULATION_OF_NCRNA_TRANSCRIPTION	1.27E-06
GOBP_MICROTUBULE_BASED_MOVEMENT	1.27E-06
GOBP_TELENCEPHALON_DEVELOPMENT	1.42E-06
GOBP_ORGANELLE_FUSION	1.46E-06
GOBP_REGULATION_OF_MRNA_PROCESSING	1.48E-06
GOBP_GLYCEROPHOSPHOLIPID_METABOLIC_PROCESS	1.59E-06
GOBP_REGULATION_OF_MEMBRANE_POTENTIAL	1.60E-06
GOBP_RENAL_SYSTEM_DEVELOPMENT	1.93E-06
GOBP_POSITIVE_REGULATION_OF_EPITHELIAL_CELL_PROLIFERATION	2.30E-06
GOBP_REGULATION_OF_MAP_KINASE_ACTIVITY	2.47E-06
GOBP_MRNA_PROCESSING	2.58E-06
GOBP_SKELETAL_MUSCLE_ORGAN_DEVELOPMENT	2.87E-06
GOBP_ENSHEATHMENT_OF_NEURONS	3.18E-06
GOBP_TISSUE_REMODELING	3.40E-06
GOBP_REGULATION_OF_DNA_RECOMBINATION	3.49E-06
GOBP_MALE_GAMETE_GENERATION	3.51E-06
GOBP_REGULATION_OF_NUCLEOCYTOPLASMIC_TRANSPORT	3.59E-06
GOBP_PROTEIN_LOCALIZATION_TO_CELL_PERIPHERY	3.67E-06
GOBP_PALLIUM_DEVELOPMENT	3.78E-06
GOBP_NEGATIVE_REGULATION_OF_KINASE_ACTIVITY	3.85E-06
GOBP_ORGANELLE_DISASSEMBLY	4.11E-06
GOBP_RHYTHMIC_PROCESS	4.19E-06
GOBP_CYTOSKELETON_DEPENDENT_INTRACELLULAR_TRANSPORT	4.53E-06
GOBP_DNA_MODIFICATION	4.80E-06
GOBP_POSITIVE_REGULATION_OF_DOUBLE_STRAND_BREAK_REPAIR	5.37E-06
GOBP_DNA_TEMPLATED_TRANSCRIPTION_INITIATION	5.54E-06
GOBP_INTRACELLULAR_GLUCOSE_HOMEOSTASIS	5.58E-06
GOBP_CELL_MATRIX_ADHESION	5.65E-06
GOBP_REGULATION_OF_TRANS_SYNAPTIC_SIGNALING	6.72E-06
GOBP_REGULATION_OF_MACROAUTOPHAGY	7.30E-06
GOBP_PROTEIN_TARGETING_TO_MITOCHONDRION	7.43E-06
GOBP_REGULATION_OF_CELL_MATRIX_ADHESION	8.25E-06
GOBP_RNA_SPLICING	8.95E-06
GOBP_REGULATION_OF_LIPID_METABOLIC_PROCESS	9.22E-06
GOBP_DNA_REPAIR	9.70E-06
GOBP_REGULATION_OF_PROTEIN_DEPHOSPHORYLATION	1.05E-05
GOBP_REGULATION_OF_CARBOHYDRATE_METABOLIC_PROCESS	1.06E-05

GOBP_RNA_3_END_PROCESSING	1.07E-05
GOBP_GASTRULATION	1.17E-05
GOBP_REGULATION_OF_CYSTEINE_TYPE_ENDOPEPTIDASE_ACTIVITY_INVOLVED_IN_APOPTOTIC_PROCESS	1.30E-05
GOBP_TRANSPORT_ALONG_MICROTUBULE	1.30E-05
GOBP_REGULATION_OF_RNA_SPLICING	1.32E-05
GOBP_CELL_PROJECTION_ASSEMBLY	1.45E-05
GOBP_NUCLEOBASE_CONTAINING_COMPOUND_TRANSPORT	1.49E-05
GOBP_INTRINSIC_APOPTOTIC_SIGNALING_PATHWAY_BY_P53_CLASS_MEDIATOR	1.81E-05
GOBP_VACUOLE_ORGANIZATION	2.06E-05
GOBP_ERK1_AND_ERK2_CASCADE	2.17E-05
GOBP_REPRODUCTIVE_SYSTEM_DEVELOPMENT	2.27E-05
GOBP_POSITIVE_REGULATION_OF_MITOTIC_CELL_CYCLE	2.38E-05
GOBP_MITOCHONDRION_ORGANIZATION	2.51E-05
GOBP_REGULATION_OF_EXOCYTOSIS	2.57E-05
GOBP_RESPONSE_TO_UV	2.65E-05
GOBP_CELL_CELL_JUNCTION_ORGANIZATION	2.81E-05
GOBP_MICROTUBULE_BASED_TRANSPORT	4.10E-05
GOBP_REGULATION_OF_MUSCLE_SYSTEM_PROCESS	4.21E-05
GOBP_MALE_SEX_DIFFERENTIATION	4.48E-05
GOBP_DENDRITE_DEVELOPMENT	4.65E-05
GOBP_DEPHOSPHORYLATION	4.69E-05
GOBP_OXIDATIVE_PHOSPHORYLATION	5.47E-05
GOBP_PROTEIN_ACYLATION	5.55E-05
GOBP_ORGANIC_ACID_CATABOLIC_PROCESS	6.61E-05
GOBP_RESPONSE_TO_ACID_CHEMICAL	7.49E-05
GOBP_PROTEIN_ACETYLATION	7.58E-05
GOBP_RECOMBINATIONAL_REPAIR	7.58E-05
GOBP_REGULATION_OF_PROTEIN_LOCALIZATION_TO_MEMBRANE	7.66E-05
GOBP_AXON_DEVELOPMENT	8.32E-05
GOBP_NEGATIVE_REGULATION_OF_POST_TRANSLATIONAL_PROTEIN_MODIFICATION	8.90E-05
GOBP_SENSORY_PERCEPTION	9.73E-05
GOBP_ESTABLISHMENT_OF_RNA_LOCALIZATION	0.00010166
GOBP_VESICLE_TARGETING	0.00010505
GOBP_STRESS_ACTIVATED_PROTEIN_KINASE_SIGNALING_CASCADE	0.00010991
GOBP_EPITHELIAL_CELL_APOPTOTIC_PROCESS	0.00011299
GOBP_EPIDERMIS_DEVELOPMENT	0.00012052
GOBP_TRNA_METABOLIC_PROCESS	0.00013237
GOBP_PROTEIN_LOCALIZATION_TO_PLASMA_MEMBRANE	0.00014692
GOBP_HEART_MORPHOGENESIS	0.00015471
GOBP_NEGATIVE_REGULATION_OF_RNA_CATABOLIC_PROCESS	0.00016427
GOBP_POSITIVE_REGULATION_OF_ESTABLISHMENT_OF_PROTEIN_LOCALIZATION	0.00016936
GOBP_NUCLEAR_EXPORT	0.00018425
GOBP_REGULATION_OF_REACTIVE_OXYGEN_SPECIES_METABOLIC_PROCESS	0.00020312
GOBP_CELLULAR_RESPIRATION	0.00020942
GOBP_REGULATION_OF_CELL_PROJECTION_ASSEMBLY	0.00021963
GOBP_SULFUR_COMPOUND_BIOSYNTHETIC_PROCESS	0.00027075
GOBP_REGULATION_OF_RAS_PROTEIN_SIGNAL_TRANSDUCTION	0.00029835
GOBP_PLATELET_ACTIVATION	0.00032209
GOBP_POSITIVE_REGULATION_OF_DNA_REPAIR	0.00037056
GOBP_ENDOSOME_ORGANIZATION	0.0003856
GOBP_AEROBIC_RESPIRATION	0.00042135
GOBP_AUTOPHAGOSOME_ORGANIZATION	0.00044153
GOBP_CELLULAR_LIPID_CATABOLIC_PROCESS	0.00044241
GOBP_TOR_SIGNALING	0.00051816
GOBP_REGULATION_OF_TRANSCRIPTION_ELONGATION_BY_RNA_POLYMERASE_II	0.0005424
GOBP_REGULATION_OF_DNA_TEMPLATED_TRANSCRIPTION_ELONGATION	0.00074335
GOBP_NUCLEAR_TRANSCRIBED_MRNA_CATABOLIC_PROCESS_DEADENYLATION_DEPENDENT_DECAY	0.00075793
GOBP_POSITIVE_REGULATION_OF_POST_TRANSLATIONAL_PROTEIN_MODIFICATION	0.0007628
GOBP_REGULATION_OF_PROTEIN_SERINE_THREONINE_KINASE_ACTIVITY	0.00079002
GOBP_ORGANELLE_FISSION	0.00081916
GOBP_ORGANOPHOSPHATE_ESTER_TRANSPORT	0.00089287
GOBP_MAINTENANCE_OF_CELL_NUMBER	0.00100081
GOBP_ENERGY_DERIVATION_BY_OXIDATION_OF_ORGANIC_COMPOUNDS	0.00111562
GOBP_STEROID_METABOLIC_PROCESS	0.00132351

GOBP_RESPONSE_TO_RADIATION	0.00132768
GOBP_RESPONSE_TO_LIGHT_STIMULUS	0.00132835
GOBP_ORGANOPHOSPHATE_METABOLIC_PROCESS	0.00158068
GOBP_REGULATION_OF_LIPID_BIOSYNTHETIC_PROCESS	0.00172634
GOBP_REGULATION_OF_CELL_JUNCTION_ASSEMBLY	0.00184534
GOBP_IMPORT_INTO_NUCLEUS	0.00214058
GOBP_HINDBRAIN_DEVELOPMENT	0.00232836
GOBP_PYRUVATE_METABOLIC_PROCESS	0.00241191
GOBP_MODULATION_BY_HOST_OF_SYMBIONT_PROCESS	0.00252416
GOBP_SEX_DIFFERENTIATION	0.00262601
GOBP_SULFUR_COMPOUND_METABOLIC_PROCESS	0.00305278
GOBP_MRNA_TRANSPORT	0.00309173
GOBP_TISSUE_MORPHOGENESIS	0.00334563
GOBP_PHOSPHATIDYLINOSITOL_METABOLIC_PROCESS	0.00368589
GOBP_MITOTIC_CELL_CYCLE_CHECKPOINT_SIGNALING	0.00384611
GOBP_REGULATION_OF_PATTERN_RECOGNITION_RECEPTOR_SIGNALING_PATHWAY	0.00419437
GOBP_CELL_FATE_COMMITMENT	0.00456409
GOBP_REGULATION_OF_STRESS_ACTIVATED_PROTEIN_KINASE_SIGNALING_CASCADE	0.00484957
GOBP_CELL_CYCLE_CHECKPOINT_SIGNALING	0.00487851
GOBP_MUSCLE_CELL_PROLIFERATION	0.00496034
GOBP_EPITHELIAL_TUBE_MORPHOGENESIS	0.00516805
GOBP_CALCIUM_ION_TRANSMEMBRANE_IMPORT_INTO_CYTOSOL	0.00600512
GOBP_REGULATION_OF_VIRAL_PROCESS	0.00624414
GOBP_POSITIVE_REGULATION_OF_INTRACELLULAR_TRANSPORT	0.00764332
GOBP_CHROMOSOME_LOCALIZATION	0.00851707
GOBP_TORC1_SIGNALING	0.00874666
GOBP_REGULATION_OF_CELL_SIZE	0.00886727
GOBP_REGULATION_OF_DOUBLE_STRAND_BREAK_REPAIR	0.01005111
GOBP_SMOOTH_MUSCLE_CELL_PROLIFERATION	0.01052743
GOBP_POSITIVE_REGULATION_OF_PROTEIN_MODIFICATION_BY_SMALL_PROTEIN_CONJUGATION_OR_REMOVAL	0.0112303
GOBP_REACTIVE_OXYGEN_SPECIES_METABOLIC_PROCESS	0.01160608
GOBP_NEGATIVE_REGULATION_OF_PROTEIN_CATABOLIC_PROCESS	0.01248885
GOBP_OSTEOBLAST_DIFFERENTIATION	0.0137346
GOBP_PEPTIDYL_LYSINE_MODIFICATION	0.01527704
GOBP_POSITIVE_REGULATION_OF_TRANSMEMBRANE_TRANSPORT	0.01943394
GOBP_PROTEIN_DEPHOSPHORYLATION	0.01987419
GOBP_ORGANOPHOSPHATE_BIOSYNTHETIC_PROCESS	0.02457913
GOBP_AMINO_ACID_METABOLIC_PROCESS	0.02521637
GOBP_POSITIVE_REGULATION_OF_PROTEIN_UBIQUITINATION	0.02529373
GOBP_POSITIVE_REGULATION_OF_DNA_TEMPLATED_TRANSCRIPTION_ELONGATION	0.02757185
GOBP_MEMBRANE_DOCKING	0.03010571
GOBP_POSITIVE_REGULATION_OF_ORGANELLE_ASSEMBLY	0.03034529
GOBP_REGULATION_OF_PROTEIN_TARGETING	0.03062698
GOBP_REGENERATION	0.03323192
GOBP_RECEPTOR_INTERNALIZATION	0.03366811
GOBP_CIRCADIAN_RHYTHM	0.03380161
GOBP_PHOSPHATIDYLINOSITOL_BIOSYNTHETIC_PROCESS	0.03659138
GOBP_MONOSACCHARIDE_METABOLIC_PROCESS	0.03876699
GOBP_POSITIVE_REGULATION_OF_INTRACELLULAR_PROTEIN_TRANSPORT	0.04074782
GOBP_POST_GOLGI_VESICLE_MEDIATED_TRANSPORT	0.04101703
GOBP_RNA_EXPORT_FROM_NUCLEUS	0.04832488
GOBP_GERM_CELL_DEVELOPMENT	0.06509256
GOBP_NEGATIVE_REGULATION_OF_GENE_EXPRESSION_EPIGENETIC	0.06663504
GOBP_MORPHOGENESIS_OF_AN_EPITHELIUM	0.07037522
GOBP_POSITIVE_REGULATION_OF_MRNA_METABOLIC_PROCESS	0.07232332
GOBP_POSITIVE_REGULATION_OF_APOPTOTIC_SIGNALING_PATHWAY	0.07276646
GOBP_SIGNAL_TRANSDUCTION_IN_RESPONSE_TO_DNA_DAMAGE	0.07367675
GOBP_CYTOSOLIC_PATTERN_RECOGNITION_RECEPTOR_SIGNALING_PATHWAY	0.08315577
GOBP_HISTONE_MODIFICATION	0.08579987
GOBP_EPIDERMAL_CELL_DIFFERENTIATION	0.09181065
GOBP_MEIOTIC_CELL_CYCLE_PROCESS	0.10619611
GOBP_REGULATION_OF_PEPTIDYL_SERINE_PHOSPHORYLATION	0.11996547
GOBP_TRANSCRIPTION_INITIATION_AT_RNA_POLYMERASE_II_PROMOTER	0.12964704
GOBP_NUCLEOTIDE_EXCISION_REPAIR	0.13920938

GOBP_RESPONSE_TO_IONIZING_RADIATION	0.14285166
GOBP_DOUBLE_STRAND_BREAK_REPAIR	0.18540098
GOBP_STEROID_BIOSYNTHETIC_PROCESS	0.20664965
GOBP_LOCOMOTORY_BEHAVIOR	0.21532036
GOBP_DNA_INTEGRITY_CHECKPOINT_SIGNALING	0.23262953
GOBP_NUCLEOBASE_CONTAINING_SMALL_MOLECULE_METABOLIC_PROCESS	0.23697594
GOBP_REGULATION_OF_PROTEIN_LOCALIZATION_TO_CELL_PERIPHERY	0.30169463
GOBP_REGULATION_OF_ENDOTHELIAL_CELL_MIGRATION	0.34538563
GOBP_CELL_CYCLE_G2_M_PHASE_TRANSITION	0.38359853
GOBP_GLUCOSE_METABOLIC_PROCESS	0.3923123
GOBP_MEIOTIC_CELL_CYCLE	0.39818074
GOBP_CELLULAR_RESPONSE_TO_RADIATION	0.40097505
GOBP_VESICLE_MEDIATED_TRANSPORT_TO_THE_PLASMA_MEMBRANE	0.41099812
GOBP_PROTEIN_K48_LINKED_UBIQUITINATION	0.4740841
GOBP_POSITIVE_REGULATION_OF_NEUROGENESIS	0.48424093
GOBP_POSITIVE_REGULATION_OF_PROTEIN_SERINE_THREONINE_KINASE_ACTIVITY	0.49206483
GOBP_REGULATION_OF_CIRCADIAN_RHYTHM	0.49495369
GOBP_REGULATION_OF_SYNAPTIC_PLASTICITY	0.50863228
GOBP_PEPTIDYL_THREONINE_MODIFICATION	0.52633059
GOBP_POSITIVE_REGULATION_OF_NERVOUS_SYSTEM_DEVELOPMENT	0.5379948
GOBP_ENDOTHELIAL_CELL_MIGRATION	0.56080974
GOBP_CELL_CELL_SIGNALING_BY_WNT	0.57501118
GOBP_MESENCHYME_DEVELOPMENT	0.58944124
GOBP_CARBOHYDRATE_DERIVATIVE_CATABOLIC_PROCESS	0.67425332
GOBP_MESENCHYMAL_CELL_DIFFERENTIATION	0.6819199
GOBP_REGULATION_OF_CELL_SHAPE	0.69164973
GOBP_LIPOPROTEIN_METABOLIC_PROCESS	0.69261637
GOBP_TYPE_I_INTERFERON_PRODUCTION	0.69954451
GOBP_PROTEIN_POLYUBIQUITINATION	0.73950391
GOBP_REGULATION_OF_DNA_REPAIR	0.74493161
GOBP_VESICLE_MEDIATED_TRANSPORT_IN_SYNAPSE	0.7571809
GOBP_MEMBRANE_LIPID_METABOLIC_PROCESS	0.76018462
GOBP_ESTABLISHMENT_OF_CELL_POLARITY	0.76705941
GOBP_ESTABLISHMENT_OR_MAINTENANCE_OF_CELL_POLARITY	0.79501724
GOBP_CELLULAR_RESPONSE_TO_LIGHT_STIMULUS	0.83057168
GOBP_POSITIVE_REGULATION_OF_STRESS_ACTIVATED_PROTEIN_KINASE_SIGNALING_CASCADE	0.8365226
GOBP_BLASTOCYST_DEVELOPMENT	0.86415279
GOBP_SKIN_DEVELOPMENT	0.87553069
GOBP_CARBOHYDRATE_CATABOLIC_PROCESS	0.88498292
GOBP_PURINE_CONTAINING_COMPOUND_METABOLIC_PROCESS	0.89529753
GOBP_PROTEIN_CONTAINING_COMPLEX_LOCALIZATION	0.92078496
GOBP_CILIUM_ORGANIZATION	0.94040496
GOBP_PROTEIN_SUMOYLATION	0.94546412
GOBP_RIBOSE_PHOSPHATE_METABOLIC_PROCESS	0.94692908
GOBP_MRNA_EXPORT_FROM_NUCLEUS	0.95626339
GOBP_EPITHELIAL_TO_MESENCHYMAL_TRANSITION	0.96072375
GOBP_ATP_METABOLIC_PROCESS	0.969604
GOBP_CELLULAR_PROCESS_INVOLVED_IN_REPRODUCTION_IN_MULTICELLULAR_ORGANISM	0.9716661
GOBP_NUCLEOSIDE_TRIPHOSPHATE_BIOSYNTHETIC_PROCESS	0.97625672
GOBP_CELLULAR_RESPONSE_TO_UV	0.97719085
GOBP_AUTOPHAGY_OF_MITOCHONDRION	0.98288847
GOBP_ATP_BIOSYNTHETIC_PROCESS	0.98510534
GOBP_REGULATION_OF_JNK_CASCADE	0.9899152
GOBP_BLOOD_VESSEL_ENDOTHELIAL_CELL_MIGRATION	0.99028607
GOBP_NUCLEOSIDE_TRIPHOSPHATE_METABOLIC_PROCESS	0.99145894
GOBP_RIBOSE_PHOSPHATE_BIOSYNTHETIC_PROCESS	0.99441514
GOBP_PROTON_MOTIVE_FORCE_DRIVEN_ATP_SYNTHESIS	0.99535713
GOBP_ENDOTHELIUM_DEVELOPMENT	0.99535868
GOBP_NUCLEOSIDE_PHOSPHATE_BIOSYNTHETIC_PROCESS	0.99591814
GOBP_PROTEIN_MODIFICATION_BY_SMALL_PROTEIN_REMOVAL	0.99614023
GOBP_HETEROCHROMATIN_ORGANIZATION	0.99687979
GOBP_ANATOMICAL_STRUCTURE_MATURATION	0.99724662
GOBP_REGULATION_OF_PROTEIN_LOCALIZATION_TO_PLASMA_MEMBRANE	0.99732218
GOBP_DEVELOPMENTAL_MATURATION	0.99755602

GOBP_JNK_CASCADE	0.9997476
GOBP_MICROTUBULE_POLYMERIZATION	0.99981908
GOBP_RESPIRATORY_SYSTEM_DEVELOPMENT	0.99989481
GOBP_ALTERNATIVE_MRNA_SPLICING_VIA_SPLICEOSOME	0.99994204
GOBP_REGULATION_OF_CELL_CYCLE_G2_M_PHASE_TRANSITION	0.99994996
GOBP_ERBB_SIGNALING_PATHWAY	0.99996657
GOBP_MITOCHONDRIAL_RESPIRATORY_CHAIN_COMPLEX_ASSEMBLY	0.99999086
GOBP_MICROTUBULE_POLYMERIZATION_OR_DEPOLYMERIZATION	0.99999791
