# Supplementary material for: GSNCASCR: An R Package to Identify Differentially Co-Expressed Curated Gene Sets with Single-Cell RNA-Seq Data
Source: Int J Mol Sci. 2025 May 16;26(10):4771. doi: 10.3390/ijms26104771 (PMC12112291; doi:10.3390/ijms26104771)
Supplement: Supplementary file 1 [file ijms-26-04771-s001.zip › Table S2-GO enrichment analysis of CD4+ T cells.pdf]

Table S2. Gene Ontology enrichment analysis of CD4$^+$ T cells.

GO term	*P* value
GOBP_DEFENSE_RESPONSE_TO_SYMBIONT	8.83E-22
GOBP_CYTOPLASMIC_TRANSLATION	1.36E-21
GOBP_REGULATION_OF_VIRAL_GENOME_REPLICATION	1.50E-19
GOBP_POSITIVE_REGULATION_OF_IMMUNE_SYSTEM_PROCESS	4.30E-19
GOBP_RESPONSE_TO_VIRUS	1.92E-18
GOBP_AMIDE_BIOSYNTHETIC_PROCESS	4.09E-18
GOBP_PEPTIDE_BIOSYNTHETIC_PROCESS	6.79E-18
GOBP_PROTEIN_ACETYLATION	1.04E-17
GOBP_NEGATIVE_REGULATION_OF_VIRAL_PROCESS	1.61E-17
GOBP_ANTIGEN_RECEPTOR_MEDIATED_SIGNALING_PATHWAY	3.30E-17
GOBP_RIBOSOME_BIOGENESIS	2.05E-16
GOBP_VIRAL_GENOME_REPLICATION	2.14E-16
GOBP_PROTEIN_ACYLATION	4.28E-16
GOBP_REGULATION_OF_IMMUNE_SYSTEM_PROCESS	4.72E-16
GOBP_PEPTIDE_METABOLIC_PROCESS	9.71E-16
GOBP_HUMORAL_IMMUNE_RESPONSE	2.27E-15
GOBP_POSITIVE_REGULATION_OF_IMMUNE_RESPONSE	2.77E-15
GOBP_TAXIS	3.46E-15
GOBP_TUBE_DEVELOPMENT	4.33E-15
GOBP_TUBE_MORPHOGENESIS	6.93E-15
GOBP_B_CELL_MEDIATED_IMMUNITY	8.60E-15
GOBP_VASCULATURE_DEVELOPMENT	1.09E-14
GOBP_PEPTIDYL_SERINE_MODIFICATION	2.65E-14
GOBP_NEGATIVE_REGULATION_OF_PROTEOLYSIS_INVOLVED_IN_PROTEIN_CATABOLIC_PROCESS	2.73E-14
GOBP_ACTIVATION_OF_IMMUNE_RESPONSE	3.26E-14
GOBP_CELL_CHEMOTAXIS	3.73E-14
GOBP_INTRINSIC_APOPTOTIC_SIGNALING_PATHWAY_IN_RESPONSE_TO_DNA_DAMAGE	4.72E-14
GOBP_LOCOMOTION	5.42E-14
GOBP_REGULATION_OF_MITOCHONDRION_ORGANIZATION	6.27E-14
GOBP_ANATOMICAL_STRUCTURE_FORMATION_INVOLVED_IN_MORPHOGENESIS	7.25E-14
GOBP_REGULATION_OF_PEPTIDYL_SERINE_PHOSPHORYLATION	7.27E-14
GOBP_MONOATOMIC_ION_TRANSPORT	1.10E-13
GOBP_NEGATIVE_REGULATION_OF_PROTEIN_CATABOLIC_PROCESS	1.12E-13
GOBP_AMIDE_METABOLIC_PROCESS	1.29E-13
GOBP_IMMUNE_RESPONSE_REGULATING_CELL_SURFACE_RECEPTOR_SIGNALING_PATHWAY	1.29E-13
GOBP_ORGANONITROGEN_COMPOUND_BIOSYNTHETIC_PROCESS	1.33E-13
GOBP_RIBOSOMAL_LARGE_SUBUNIT_BIOGENESIS	1.46E-13
GOBP_IMMUNE_RESPONSE_REGULATING_SIGNALING_PATHWAY	1.56E-13
GOBP_RIBONUCLEOPROTEIN_COMPLEX_BIOGENESIS	1.60E-13
GOBP_REGULATION_OF_IMMUNE_RESPONSE	2.14E-13
GOBP_BLOOD_VESSEL_MORPHOGENESIS	2.90E-13
GOBP_REGULATION_OF_CELLULAR_RESPONSE_TO_STRESS	3.81E-13
GOBP_REGULATION_OF_PATTERN_RECOGNITION_RECEPTOR_SIGNALING_PATHWAY	4.30E-13
GOBP_ADAPTIVE_IMMUNE_RESPONSE_BASED_ON_SOMATIC_RECOMBINATION_OF_IMMUNE_RECEPTORS_BUILT_ FROM_IMMUNOGLOBULIN_SUPERFAMILY_DOMAINS	4.98E-13
GOBP_LEUKOCYTE_MEDIATED_IMMUNITY	7.74E-13
GOBP_RESPONSE_TO_TYPE_I_INTERFERON	7.86E-13
GOBP_REGULATION_OF_RESPONSE_TO_STRESS	8.58E-13
GOBP_ADAPTIVE_IMMUNE_RESPONSE	9.02E-13
GOBP_INTERFERON_MEDIATED_SIGNALING_PATHWAY	9.97E-13
GOBP_ERYTHROCYTE_HOMEOSTASIS	1.21E-12
GOBP_REGULATION_OF_PROTEIN_MODIFICATION_PROCESS	1.24E-12
GOBP_ENDOTHELIAL_CELL_MIGRATION	1.50E-12
GOBP_PROTEIN_PHOSPHORYLATION	1.55E-12
GOBP_IMMUNE_EFFECTOR_PROCESS	1.66E-12
GOBP_NEGATIVE_REGULATION_OF_APOPTOTIC_SIGNALING_PATHWAY	1.71E-12
GOBP_CIRCULATORY_SYSTEM_DEVELOPMENT	1.72E-12
GOBP_LEUKOCYTE_CHEMOTAXIS	1.97E-12
GOBP_CELL_MOTILITY	2.26E-12

GOBP_POSITIVE_REGULATION_OF_EPITHELIAL_CELL_MIGRATION	2.57E-12
GOBP_POSITIVE_REGULATION_OF_CYTOKINE_PRODUCTION	2.63E-12
GOBP_MONOATOMIC_ION_TRANSMEMBRANE_TRANSPORT	2.81E-12
GOBP_RESPONSE_TO_SALT	3.07E-12
GOBP_ESTABLISHMENT_OF_PROTEIN_LOCALIZATION	3.12E-12
GOBP_REGULATION_OF_CELL_DIFFERENTIATION	3.25E-12
GOBP_BIOLOGICAL_PROCESS_INVOLVED_IN_INTERSPECIES_INTERACTION_BETWEEN_ORGANISMS	3.57E-12
GOBP_LEUKOCYTE_MIGRATION	3.61E-12
GOBP_CYTOSOLIC_PATTERN_RECOGNITION_RECEPTOR_SIGNALING_PATHWAY	3.99E-12
GOBP_REGULATION_OF_VIRAL_PROCESS	4.24E-12
GOBP_CELLULAR_RESPONSE_TO_STRESS	4.56E-12
GOBP_DEFENSE_RESPONSE_TO_OTHER_ORGANISM	5.32E-12
GOBP_LYMPHOCYTE_MEDIATED_IMMUNITY	5.52E-12
GOBP_MONOATOMIC_CATION_TRANSPORT	6.03E-12
GOBP_REGULATION_OF_HEMOPOIESIS	6.35E-12
GOBP_APOPTOTIC_SIGNALING_PATHWAY	6.96E-12
GOBP_REGULATION_OF_ENDOTHELIAL_CELL_MIGRATION	7.64E-12
GOBP_NEGATIVE_REGULATION_OF_NUCLEOBASE_CONTAINING_COMPOUND_METABOLIC_PROCESS	8.01E-12
GOBP_NEGATIVE_REGULATION_OF_CATABOLIC_PROCESS	9.30E-12
GOBP_INTRACELLULAR_RECEPTOR_SIGNALING_PATHWAY	9.82E-12
GOBP_PROTEIN_RNA_COMPLEX_ORGANIZATION	1.49E-11
GOBP_POSITIVE_REGULATION_OF_PROTEIN_MODIFICATION_PROCESS	1.51E-11
GOBP_RESPONSE_TO_CYTOKINE	1.54E-11
GOBP_REGULATION_OF_CELL_ADHESION	1.63E-11
GOBP_AMEBOIDAL_TYPE_CELL_MIGRATION	1.64E-11
GOBP_POSITIVE_REGULATION_OF_CELL_DIFFERENTIATION	1.72E-11
GOBP_INNATE_IMMUNE_RESPONSE	1.74E-11
GOBP_INTRACELLULAR_PROTEIN_TRANSPORT	1.83E-11
GOBP_DEFENSE_RESPONSE	1.95E-11
GOBP_RIBOSOMAL_SMALL_SUBUNIT_BIOGENESIS	1.99E-11
GOBP_PLATELET_ACTIVATION	2.10E-11
GOBP_RRNA_METABOLIC_PROCESS	2.32E-11
GOBP_MACROMOLECULE_CATABOLIC_PROCESS	2.55E-11
GOBP_POSITIVE_REGULATION_OF_DEVELOPMENTAL_PROCESS	3.25E-11
GOBP_MYELOID_CELL_HOMEOSTASIS	3.33E-11
GOBP_CELL_ADHESION	4.22E-11
GOBP_MYELOID_LEUKOCYTE_MIGRATION	4.73E-11
GOBP_CELL_CELL_JUNCTION_ORGANIZATION	5.25E-11
GOBP_NEGATIVE_REGULATION_OF_RNA_BIOSYNTHETIC_PROCESS	5.28E-11
GOBP_PROTEOLYSIS_INVOLVED_IN_PROTEIN_CATABOLIC_PROCESS	6.08E-11
GOBP_MITOTIC_SISTER_CHROMATID_SEGREGATION	8.03E-11
GOBP_MONOATOMIC_ION_HOMEOSTASIS	8.24E-11
GOBP_REGULATION_OF_SUPRAMOLECULAR_FIBER_ORGANIZATION	8.45E-11
GOBP_NEGATIVE_REGULATION_OF_PROTEIN_METABOLIC_PROCESS	8.65E-11
GOBP_MONOATOMIC_CATION_TRANSMEMBRANE_TRANSPORT	9.38E-11
GOBP_VIRAL_LIFE_CYCLE	1.00E-10
GOBP_PROTEIN_CATABOLIC_PROCESS	1.11E-10
GOBP_CELLULAR_CATABOLIC_PROCESS	1.13E-10
GOBP_POSITIVE_REGULATION_OF_LOCOMOTION	1.28E-10
GOBP_CELLULAR_HOMEOSTASIS	1.29E-10
GOBP_ORGANONITROGEN_COMPOUND_CATABOLIC_PROCESS	1.31E-10
GOBP_PROTON_TRANSMEMBRANE_TRANSPORT	1.33E-10
GOBP_REGULATION_OF_T_CELL_ACTIVATION	1.67E-10
GOBP_CELL_ACTIVATION	2.04E-10
GOBP_REGULATION_OF_APOPTOTIC_SIGNALING_PATHWAY	2.15E-10
GOBP_TISSUE_MIGRATION	2.16E-10
GOBP_REGULATION_OF_RESPONSE_TO_CYTOKINE_STIMULUS	2.47E-10
GOBP_TRANSMEMBRANE_TRANSPORT	2.59E-10
GOBP_PROCESS_UTILIZING_AUTOPHAGIC_MECHANISM	2.65E-10
GOBP_REGULATION_OF_PROTEIN_SERINE_THREONINE_KINASE_ACTIVITY	2.75E-10
GOBP_REGULATION_OF_CELL_CELL_ADHESION	2.94E-10
GOBP_REGULATION_OF_MICROTUBULE_BASED_PROCESS	3.35E-10
GOBP_REGULATION_OF_PROTEIN_STABILITY	3.70E-10
GOBP_REGULATION_OF_MONOATOMIC_ION_TRANSPORT	3.89E-10

GOBP_GLYCOPROTEIN_BIOSYNTHETIC_PROCESS	4.09E-10
GOBP_REGULATION_OF_POST_TRANSLATIONAL_PROTEIN_MODIFICATION	4.79E-10
GOBP_REGULATION_OF_CHEMOTAXIS	4.80E-10
GOBP_REGULATION_OF_LYMPHOCYTE_ACTIVATION	5.26E-10
GOBP_REGULATION_OF_CATABOLIC_PROCESS	5.35E-10
GOBP_POSITIVE_REGULATION_OF_RNA_METABOLIC_PROCESS	5.51E-10
GOBP_MONOCARBOXYLIC_ACID_BIOSYNTHETIC_PROCESS	5.55E-10
GOBP_REGULATION_OF_LEUKOCYTE_MIGRATION	5.79E-10
GOBP_REGULATION_OF_PROTEIN_UBIQUITINATION	6.75E-10
GOBP_REGULATION_OF_EXTRINSIC_APOPTOTIC_SIGNALING_PATHWAY	7.43E-10
GOBP_REGULATION_OF_LYMPHOCYTE_DIFFERENTIATION	7.46E-10
GOBP_REGULATION_OF_PHOSPHORYLATION	7.88E-10
GOBP_REGULATION_OF_RNA_SPLICING	8.41E-10
GOBP_REGULATION_OF_UBIQUITIN_DEPENDENT_PROTEIN_CATABOLIC_PROCESS	8.81E-10
GOBP_REGULATION_OF_EPITHELIAL_CELL_MIGRATION	8.92E-10
GOBP_PROTEIN_STABILIZATION	9.08E-10
GOBP_HEMOSTASIS	9.19E-10
GOBP_APOPTOTIC_PROCESS	9.91E-10
GOBP_NEGATIVE_REGULATION_OF_INTRINSIC_APOPTOTIC_SIGNALING_PATHWAY	9.97E-10
GOBP_NEGATIVE_REGULATION_OF_PROGRAMMED_CELL_DEATH	1.06E-09
GOBP_AXO_DENDRITIC_TRANSPORT	1.17E-09
GOBP_RESPONSE_TO_WOUNDING	1.40E-09
GOBP_REGULATION_OF_INTRACELLULAR_SIGNAL_TRANSDUCTION	1.50E-09
GOBP_HISTONE_MODIFICATION	1.53E-09
GOBP_CELL_JUNCTION_ORGANIZATION	1.54E-09
GOBP_REGULATION_OF_PROTEASOMAL_UBIQUITIN_DEPENDENT_PROTEIN_CATABOLIC_PROCESS	1.58E-09
GOBP_REGULATION_OF_PROGRAMMED_CELL_DEATH	1.62E-09
GOBP_POSITIVE_REGULATION_OF_AUTOPHAGY	1.69E-09
GOBP_PROTEOLYSIS	2.04E-09
GOBP_NEGATIVE_REGULATION_OF_PROTEOLYSIS	2.16E-09
GOBP_INTRINSIC_APOPTOTIC_SIGNALING_PATHWAY	2.52E-09
GOBP_REGULATION_OF_CELL_DEVELOPMENT	2.59E-09
GOBP_SPINDLE_ASSEMBLY	2.71E-09
GOBP_CELLULAR_RESPONSE_TO_TYPE_II_INTERFERON	2.79E-09
GOBP_POSITIVE_REGULATION_OF_LEUKOCYTE_MIGRATION	2.88E-09
GOBP_NON_MEMBRANE_BOUNDED_ORGANELLE_ASSEMBLY	2.95E-09
GOBP_DNA_DAMAGE_RESPONSE	3.06E-09
GOBP_REGULATION_OF_CELL_ACTIVATION	3.61E-09
GOBP_REGULATION_OF_TRANSPORT	3.94E-09
GOBP_CELL_CELL_ADHESION	3.98E-09
GOBP_MEMBRANE_ORGANIZATION	4.06E-09
GOBP_NEGATIVE_REGULATION_OF_TRANSCRIPTION_BY_RNA_POLYMERASE_II	4.28E-09
GOBP_BLASTOCYST_DEVELOPMENT	4.48E-09
GOBP_REGULATION_OF_PROTEIN_CATABOLIC_PROCESS	4.88E-09
GOBP_DNA_RECOMBINATION	4.89E-09
GOBP_POSITIVE_REGULATION_OF_PHOSPHORUS_METABOLIC_PROCESS	5.41E-09
GOBP_VIRAL_PROCESS	5.49E-09
GOBP_REGULATION_OF_PROTEIN_LOCALIZATION_TO_MEMBRANE	5.84E-09
GOBP_PROTEIN_CONTAINING_COMPLEX_ASSEMBLY	6.16E-09
GOBP_REGULATION_OF_MICROTUBULE_CYTOSKELETON_ORGANIZATION	6.26E-09
GOBP_IN_UTERO_EMBRYONIC_DEVELOPMENT	6.35E-09
GOBP_AEROBIC_RESPIRATION	6.40E-09
GOBP_EMBRYO_DEVELOPMENT_ENDING_IN_BIRTH_OR_EGG_HATCHING	6.54E-09
GOBP_REGULATION_OF_CYTOSKELETON_ORGANIZATION	7.27E-09
GOBP_REGULATION_OF_MULTICELLULAR_ORGANISMAL_DEVELOPMENT	7.70E-09
GOBP_POSITIVE_REGULATION_OF_POST_TRANSLATIONAL_PROTEIN_MODIFICATION	7.82E-09
GOBP_POSITIVE_REGULATION_OF_RESPONSE_TO_EXTERNAL_STIMULUS	8.30E-09
GOBP_ANIMAL_ORGAN_MORPHOGENESIS	8.40E-09
GOBP_POSITIVE_REGULATION_OF_PRODUCTION_OF_MOLECULAR_MEDIATOR_OF_IMMUNE_RESPONSE	8.53E-09
GOBP_REGULATION_OF_PHOSPHORUS_METABOLIC_PROCESS	9.58E-09
GOBP_REGULATION_OF_PROTEOLYSIS_INVOLVED_IN_PROTEIN_CATABOLIC_PROCESS	9.72E-09
GOBP_REGULATION_OF_LEUKOCYTE_DIFFERENTIATION	9.91E-09
GOBP_SULFUR_COMPOUND_METABOLIC_PROCESS	1.23E-08
GOBP_EXTRINSIC_APOPTOTIC_SIGNALING_PATHWAY	1.32E-08

GOBP_HEMOPOIESIS	1.36E-08
GOBP_NEGATIVE_REGULATION_OF_POST_TRANSLATIONAL_PROTEIN_MODIFICATION	1.39E-08
GOBP_MACROAUTOPHAGY	1.42E-08
GOBP_REGULATION_OF_TRANSMEMBRANE_TRANSPORT	1.59E-08
GOBP_REGULATION_OF_RESPONSE_TO_EXTERNAL_STIMULUS	1.63E-08
GOBP_POSITIVE_REGULATION_OF_CELL_ACTIVATION	1.72E-08
GOBP_PROTEIN_DNA_COMPLEX_ASSEMBLY	1.72E-08
GOBP_INTRACELLULAR_TRANSPORT	1.72E-08
GOBP_WOUND_HEALING	1.80E-08
GOBP_PROTEASOMAL_PROTEIN_CATABOLIC_PROCESS	1.98E-08
GOBP_VESICLE_MEDIATED_TRANSPORT	2.01E-08
GOBP_POSITIVE_REGULATION_OF_PHOSPHORYLATION	2.01E-08
GOBP_NEGATIVE_REGULATION_OF_CYTOSKELETON_ORGANIZATION	2.05E-08
GOBP_SIGNAL_TRANSDUCTION_BY_P53_CLASS_MEDIATOR	2.06E-08
GOBP_POSITIVE_REGULATION_OF_LYMPHOCYTE_ACTIVATION	2.31E-08
GOBP_REGULATION_OF_PROTEIN_CONTAINING_COMPLEX_ASSEMBLY	2.33E-08
GOBP_POSITIVE_REGULATION_OF_NF_KAPPAB_TRANSCRIPTION_FACTOR_ACTIVITY	2.34E-08
GOBP_EMBRYO_DEVELOPMENT	2.48E-08
GOBP_SENSORY_SYSTEM_DEVELOPMENT	2.50E-08
GOBP_SENSORY_ORGAN_DEVELOPMENT	2.65E-08
GOBP_LOCALIZATION_WITHIN_MEMBRANE	2.85E-08
GOBP_SISTER_CHROMATID_SEGREGATION	2.94E-08
GOBP_SPINDLE_ORGANIZATION	3.06E-08
GOBP_APOPTOTIC_MITOCHONDRIAL_CHANGES	3.13E-08
GOBP_PHOSPHORYLATION	3.23E-08
GOBP_CELL_JUNCTION_ASSEMBLY	3.65E-08
GOBP_POSITIVE_REGULATION_OF_TRANSCRIPTION_BY_RNA_POLYMERASE_II	3.69E-08
GOBP_POSITIVE_REGULATION_OF_PROTEIN_KINASE_ACTIVITY	3.90E-08
GOBP_POSITIVE_REGULATION_OF_DEFENSE_RESPONSE	3.94E-08
GOBP_REGULATION_OF_ENDOCYTOSIS	4.01E-08
GOBP_POSITIVE_REGULATION_OF_ENDOCYTOSIS	4.13E-08
GOBP_REGULATION_OF_LOCOMOTION	4.65E-08
GOBP_CELLULAR_RESPONSE_TO_LIPID	4.97E-08
GOBP_POSITIVE_REGULATION_OF_SIGNALING	5.09E-08
GOBP_POSITIVE_REGULATION_OF_GENE_EXPRESSION	5.59E-08
GOBP_REGULATION_OF_MONOATOMIC_ION_TRANSMEMBRANE_TRANSPORT	6.21E-08
GOBP_MITOTIC_NUCLEAR_DIVISION	6.44E-08
GOBP_CHROMOSOME_ORGANIZATION	7.19E-08
GOBP_REGULATION_OF_PROTEASOMAL_PROTEIN_CATABOLIC_PROCESS	7.40E-08
GOBP_REGULATION_OF_RESPONSE_TO_ENDOPLASMIC_RETICULUM_STRESS	7.42E-08
GOBP_RESPONSE_TO_BACTERIUM	8.14E-08
GOBP_REGULATION_OF_CELL_CYCLE_G1_S_PHASE_TRANSITION	8.48E-08
GOBP_STEM_CELL_DIFFERENTIATION	8.66E-08
GOBP_POSITIVE_REGULATION_OF_CELL_ADHESION	8.76E-08
GOBP_VACUOLAR_TRANSPORT	9.53E-08
GOBP_POSITIVE_REGULATION_OF_INFLAMMATORY_RESPONSE	9.63E-08
GOBP_POSITIVE_REGULATION_OF_CATABOLIC_PROCESS	9.87E-08
GOBP_INORGANIC_ION_HOMEOSTASIS	1.01E-07
GOBP_GENERATION_OF_NEURONS	1.08E-07
GOBP_POSITIVE_REGULATION_OF_CELL_POPULATION_PROLIFERATION	1.19E-07
GOBP_T_CELL_ACTIVATION	1.25E-07
GOBP_ORGANELLE_ASSEMBLY	1.33E-07
GOBP_REGULATION_OF_GENERATION_OF_PRECURSOR_METABOLITES_AND_ENERGY	1.44E-07
GOBP_LYMPHOCYTE_ACTIVATION	1.49E-07
GOBP_PROTEIN_LOCALIZATION_TO_CELL_PERIPHERY	1.59E-07
GOBP_REGULATION_OF_CELL_MORPHOGENESIS	1.73E-07
GOBP_REGULATION_OF_MYELOID_CELL_DIFFERENTIATION	1.84E-07
GOBP_LEUKOCYTE_CELL_CELL_ADHESION	1.84E-07
GOBP_MONONUCLEAR_CELL_MIGRATION	1.84E-07
GOBP_AUTOPHAGY_OF_MITOCHONDRION	1.85E-07
GOBP_CELLULAR_RESPIRATION	1.88E-07
GOBP_REGULATION_OF_ACTIN_FILAMENT_BASED_PROCESS	1.88E-07
GOBP_NEGATIVE_REGULATION_OF_ORGANELLE_ORGANIZATION	1.93E-07
GOBP_POSITIVE_REGULATION_OF_CELL_DEVELOPMENT	2.10E-07

GOBP_RNA_SPLICING	2.33E-07
GOBP_REGULATION_OF_TRANSMEMBRANE_RECEPTOR_PROTEIN_SERINE_THREONINE_KINASE_SIGNALING_PATHWAY	2.57E-07
GOBP_PURINE_CONTAINING_COMPOUND_METABOLIC_PROCESS	2.68E-07
GOBP_NUCLEAR_CHROMOSOME_SEGREGATION	2.78E-07
GOBP_GLYCOPROTEIN_METABOLIC_PROCESS	2.79E-07
GOBP_REGULATION_OF_CELLULAR_RESPONSE_TO_GROWTH_FACTOR_STIMULUS	2.80E-07
GOBP_POSITIVE_REGULATION_OF_MULTICELLULAR_ORGANISMAL_PROCESS	2.82E-07
GOBP_CELL_FATE_COMMITMENT	2.90E-07
GOBP_MITOCHONDRIAL_MEMBRANE_ORGANIZATION	3.21E-07
GOBP_PHOSPHATIDYLINOSITOL_3_KINASE_PROTEIN_KINASE_B_SIGNAL_TRANSDUCTION	3.27E-07
GOBP_POSITIVE_REGULATION_OF_RESPONSE_TO_BIOTIC_STIMULUS	3.39E-07
GOBP_RESPONSE_TO_LIPID	3.61E-07
GOBP_AMINO_ACID_METABOLIC_PROCESS	4.31E-07
GOBP_MITOCHONDRION_ORGANIZATION	4.59E-07
GOBP_RESPONSE_TO_GROWTH_FACTOR	4.81E-07
GOBP_MITOCHONDRIAL_TRANSPORT	4.95E-07
GOBP_RESPONSE_TO_STEROID_HORMONE	5.49E-07
GOBP_PROTEIN_LOCALIZATION_TO_PLASMA_MEMBRANE	6.01E-07
GOBP_REGULATION_OF_CELL_CYCLE	6.48E-07
GOBP_MRNA_TRANSPORT	6.49E-07
GOBP_MITOTIC_CYTOKINESIS	6.85E-07
GOBP_IMPORT_INTO_CELL	7.23E-07
GOBP_REGULATION_OF_DEFENSE_RESPONSE	7.77E-07
GOBP_REGULATION_OF_ANATOMICAL_STRUCTURE_MORPHOGENESIS	9.00E-07
GOBP_RNA_LOCALIZATION	9.06E-07
GOBP_ESTABLISHMENT_OF_RNA_LOCALIZATION	9.29E-07
GOBP_POSITIVE_REGULATION_OF_KINASE_ACTIVITY	9.94E-07
GOBP_POSITIVE_REGULATION_OF_PROTEIN_METABOLIC_PROCESS	1.05E-06
GOBP_MONONUCLEAR_CELL_DIFFERENTIATION	1.09E-06
GOBP_CYTOSKELETON_DEPENDENT_CYTOKINESIS	1.11E-06
GOBP_POST_TRANSLATIONAL_PROTEIN_MODIFICATION	1.17E-06
GOBP_HOMEOSTATIC_PROCESS	1.24E-06
GOBP_NCRNA_PROCESSING	1.46E-06
GOBP_NEGATIVE_REGULATION_OF_INTRACELLULAR_SIGNAL_TRANSDUCTION	1.49E-06
GOBP_HINDBRAIN_DEVELOPMENT	1.61E-06
GOBP_POSITIVE_REGULATION_OF_HEMOPOIESIS	1.65E-06
GOBP_POSITIVE_REGULATION_OF_GROWTH	1.77E-06
GOBP_NUCLEOBASE_CONTAINING_SMALL_MOLECULE_METABOLIC_PROCESS	2.09E-06
GOBP_LEUKOCYTE_DIFFERENTIATION	2.13E-06
GOBP_REGULATION_OF_DNA_TEMPLATED_TRANSCRIPTION_INITIATION	2.15E-06
GOBP_ENERGY_DERIVATION_BY_OXIDATION_OF_ORGANIC_COMPOUNDS	2.22E-06
GOBP_POSITIVE_REGULATION_OF_PROTEIN_SERINE_THREONINE_KINASE_ACTIVITY	2.31E-06
GOBP_LIPID_BIOSYNTHETIC_PROCESS	2.36E-06
GOBP_INFLAMMATORY_RESPONSE	2.37E-06
GOBP_DOUBLE_STRAND_BREAK_REPAIR	2.67E-06
GOBP_NEGATIVE_REGULATION_OF_PROTEIN_MODIFICATION_PROCESS	2.69E-06
GOBP_ORGANIC_ACID_METABOLIC_PROCESS	3.11E-06
GOBP_POSITIVE_REGULATION_OF_DNA_BINDING_TRANSCRIPTION_FACTOR_ACTIVITY	3.13E-06
GOBP_REGULATION_OF_B_CELL_ACTIVATION	3.21E-06
GOBP_POSITIVE_REGULATION_OF_LEUKOCYTE_CELL_CELL_ADHESION	3.45E-06
GOBP_HEAD_DEVELOPMENT	3.57E-06
GOBP_TRANSLATIONAL_INITIATION	3.76E-06
GOBP_MITOTIC_SPINDLE_ORGANIZATION	3.91E-06
GOBP_REGULATION_OF_ACTIN_FILAMENT_ORGANIZATION	3.93E-06
GOBP_PROTEIN_TARGETING	4.29E-06
GOBP_HOMEOSTASIS_OF_NUMBER_OF_CELLS	4.84E-06
GOBP_POSITIVE_REGULATION_OF_EPITHELIAL_CELL_PROLIFERATION	5.26E-06
GOBP_ALCOHOL_METABOLIC_PROCESS	5.28E-06
GOBP_POSITIVE_REGULATION_OF_CELL_CELL_ADHESION	6.51E-06
GOBP_ERAD_PATHWAY	6.97E-06
GOBP_POSITIVE_REGULATION_OF_CELLULAR_CATABOLIC_PROCESS	7.46E-06
GOBP_ALPHA_BETA_T_CELL_ACTIVATION	7.49E-06
GOBP_CELL_MORPHOGENESIS	7.81E-06
GOBP_POSITIVE_REGULATION_OF_MOLECULAR_FUNCTION	8.19E-06

GOBP_SUPRAMOLECULAR_FIBER_ORGANIZATION	8.27E-06
GOBP_ACTIVATION_OF_INNATE_IMMUNE_RESPONSE	8.29E-06
GOBP_POSITIVE_REGULATION_OF_TRANSPORT	8.49E-06
GOBP_GERM_CELL_DEVELOPMENT	8.52E-06
GOBP_REGULATION_OF_INTRINSIC_APOPTOTIC_SIGNALING_PATHWAY	8.73E-06
GOBP_REGULATION_OF_ALPHA_BETA_T_CELL_ACTIVATION	8.76E-06
GOBP_NEGATIVE_REGULATION_OF_AMIDE_METABOLIC_PROCESS	9.07E-06
GOBP_OSSIFICATION	9.38E-06
GOBP_REGULATION_OF_NEURON_DIFFERENTIATION	9.56E-06
GOBP_REGULATION_OF_LEUKOCYTE_PROLIFERATION	9.98E-06
GOBP_ORGANIC_HYDROXY_COMPOUND_METABOLIC_PROCESS	1.02E-05
GOBP_CYTOKINE_PRODUCTION	1.05E-05
GOBP_REGULATION_OF_IMMUNE_EFFECTOR_PROCESS	1.05E-05
GOBP_POSITIVE_REGULATION_OF_TRANSMEMBRANE_TRANSPORT	1.05E-05
GOBP_RESPONSE_TO_HEAT	1.10E-05
GOBP_CHAPERONE_MEDIATED_PROTEIN_FOLDING	1.18E-05
GOBP_T_CELL_DIFFERENTIATION	1.20E-05
GOBP_UBIQUITIN_DEPENDENT_ERAD_PATHWAY	1.27E-05
GOBP_NCRNA_METABOLIC_PROCESS	1.30E-05
GOBP_NEUROGENESIS	1.45E-05
GOBP_POSITIVE_REGULATION_OF_CELL_PROJECTION_ORGANIZATION	1.65E-05
GOBP_REGULATION_OF_BODY_FLUID_LEVELS	1.69E-05
GOBP_CELLULAR_RESPONSE_TO_ORGANIC_CYCLIC_COMPOUND	1.71E-05
GOBP_CELL_CELL_SIGNALING_BY_WNT	1.72E-05
GOBP_EXPORT_FROM_CELL	1.76E-05
GOBP_REGULATION_OF_PROTEIN_DEPHOSPHORYLATION	1.81E-05
GOBP_REGULATION_OF_KINASE_ACTIVITY	1.87E-05
GOBP_LEUKOCYTE_MEDIATED_CYTOTOXICITY	1.90E-05
GOBP_GENERATION_OF_PRECURSOR_METABOLITES_AND_ENERGY	1.93E-05
GOBP_REGULATION_OF_PROTEIN_LOCALIZATION_TO_CELL_PERIPHERY	1.93E-05
GOBP_PROTEIN_MODIFICATION_BY_SMALL_PROTEIN_REMOVAL	1.97E-05
GOBP_REGULATION_OF_INNATE_IMMUNE_RESPONSE	1.99E-05
GOBP_REGULATION_OF_SIGNAL_TRANSDUCTION_BY_P53_CLASS_MEDIATOR	1.99E-05
GOBP_REGULATION_OF_CELLULAR_CATABOLIC_PROCESS	2.09E-05
GOBP_REGULATION_OF_PROTEIN_BINDING	2.11E-05
GOBP_LEUKOCYTE_PROLIFERATION	2.19E-05
GOBP_CHROMOSOME_SEGREGATION	2.19E-05
GOBP_NCRNA_TRANSCRIPTION	2.20E-05
GOBP_B_CELL_ACTIVATION	2.25E-05
GOBP_PROTEIN_TARGETING_TO_MEMBRANE	2.32E-05
GOBP_CARBOHYDRATE_DERIVATIVE_METABOLIC_PROCESS	2.32E-05
GOBP_ALPHA_BETA_T_CELL_DIFFERENTIATION	2.40E-05
GOBP_REGULATION_OF_REPRODUCTIVE_PROCESS	2.45E-05
GOBP_DEFENSE_RESPONSE_TO_BACTERIUM	2.46E-05
GOBP_REGULATION_OF_RESPONSE_TO_BIOTIC_STIMULUS	2.67E-05
GOBP_ENDOSOME_ORGANIZATION	2.77E-05
GOBP_REGULATION_OF_DNA_BINDING_TRANSCRIPTION_FACTOR_ACTIVITY	2.80E-05
GOBP_REGULATION_OF_METAL_ION_TRANSPORT	2.82E-05
GOBP_REGULATION_OF_MONOATOMIC_CATION_TRANSMEMBRANE_TRANSPORT	2.89E-05
GOBP_NEGATIVE_REGULATION_OF_RNA_CATABOLIC_PROCESS	3.47E-05
GOBP_ENDOCYTOSIS	3.48E-05
GOBP_AUTOPHAGOSOME_ORGANIZATION	3.51E-05
GOBP_VESICLE_LOCALIZATION	3.58E-05
GOBP_MYELOID_CELL_DIFFERENTIATION	3.70E-05
GOBP_REGULATION_OF_AUTOPHAGY	3.83E-05
GOBP_MICROTUBULE_BASED_TRANSPORT	3.88E-05
GOBP_REGULATION_OF_CELLULAR_LOCALIZATION	3.90E-05
GOBP_ORGANELLE_LOCALIZATION	4.34E-05
GOBP_TRANSPORT_ALONG_MICROTUBULE	4.51E-05
GOBP_CARBOHYDRATE_DERIVATIVE_BIOSYNTHETIC_PROCESS	4.70E-05
GOBP_NEURON_DEVELOPMENT	4.80E-05
GOBP_REGULATION_OF_DNA_REPAIR	4.82E-05
GOBP_NEGATIVE_REGULATION_OF_SIGNALING	4.89E-05
GOBP_REGULATION_OF_DNA_METABOLIC_PROCESS	5.70E-05

GOBP_POSTSYNAPSE_ORGANIZATION	5.81E-05
GOBP_SMALL_MOLECULE_BIOSYNTHETIC_PROCESS	5.83E-05
GOBP_CYTOSKELETON_ORGANIZATION	5.84E-05
GOBP_PROTEIN_TARGETING_TO_MITOCHONDRION	6.65E-05
GOBP_ENTRY_INTO_HOST	6.96E-05
GOBP_ESTABLISHMENT_OF_PROTEIN_LOCALIZATION_TO_ORGANELLE	6.98E-05
GOBP_REGULATION_OF_VESICLE_MEDIATED_TRANSPORT	7.04E-05
GOBP_CELLULAR_RESPONSE_TO_INORGANIC_SUBSTANCE	7.32E-05
GOBP_NEGATIVE_REGULATION_OF_RESPONSE_TO_STIMULUS	7.41E-05
GOBP_POSITIVE_REGULATION_OF_IMMUNE_EFFECTOR_PROCESS	7.60E-05
GOBP_PROTEIN_LOCALIZATION_TO_MITOCHONDRION	7.62E-05
GOBP_REGULATION_OF_CELL_SUBSTRATE_ADHESION	7.86E-05
GOBP_REGULATION_OF_ADAPTIVE_IMMUNE_RESPONSE	8.48E-05
GOBP_ESTABLISHMENT_OF_PROTEIN_LOCALIZATION_TO_MEMBRANE	8.59E-05
GOBP_PROTEIN_DNA_COMPLEX_ORGANIZATION	8.79E-05
GOBP_POSITIVE_REGULATION_OF_PEPTIDASE_ACTIVITY	8.93E-05
GOBP_ORGANIC_CYCLIC_COMPOUND_CATABOLIC_PROCESS	9.32E-05
GOBP_REGULATION_OF_PROTEIN_LOCALIZATION_TO_PLASMA_MEMBRANE	0.00011431
GOBP_RNA_PROCESSING	0.00011475
GOBP_T_CELL_ACTIVATION_INVOLVED_IN_IMMUNE_RESPONSE	0.00011619
GOBP_POSITIVE_REGULATION_OF_CELL_CYCLE_PHASE_TRANSITION	0.00012543
GOBP_REGULATION_OF_CELL_PROJECTION_ORGANIZATION	0.00013413
GOBP_CARBOHYDRATE_BIOSYNTHETIC_PROCESS	0.0001343
GOBP_NEGATIVE_REGULATION_OF_SUPRAMOLECULAR_FIBER_ORGANIZATION	0.00015389
GOBP_RESPONSE_TO_ABIOTIC_STIMULUS	0.00016268
GOBP_DNA_METABOLIC_PROCESS	0.00017814
GOBP_REGULATION_OF_DNA_RECOMBINATION	0.00018469
GOBP_RIBOSE_PHOSPHATE_METABOLIC_PROCESS	0.0001926
GOBP_RESPONSE_TO_ENDOPLASMIC_RETICULUM_STRESS	0.00019733
GOBP_PEPTIDYL_THREONINE_MODIFICATION	0.00020939
GOBP_MODULATION_BY_HOST_OF_SYMBIONT_PROCESS	0.00021084
GOBP_POSITIVE_REGULATION_OF_PROTEIN_CONTAINING_COMPLEX_ASSEMBLY	0.00021217
GOBP_NUCLEOSIDE_PHOSPHATE_BIOSYNTHETIC_PROCESS	0.00021558
GOBP_CELL_CELL_SIGNALING	0.0002223
GOBP_NEGATIVE_REGULATION_OF_NEURON_APOPTOTIC_PROCESS	0.00023186
GOBP_POSITIVE_REGULATION_OF_PHOSPHATIDYLINOSITOL_3_KINASE_PROTEIN_KINASE_B_SIGNAL_TRANSDUCTION	0.00023521
GOBP_DNA_REPAIR	0.00023674
GOBP_SECRETION	0.00025765
GOBP_TRANSMEMBRANE_RECEPTOR_PROTEIN_SERINE_THREONINE_KINASE_SIGNALING_PATHWAY	0.00027033
GOBP_REGULATION_OF_JNK_CASCADE	0.00028041
GOBP_CARBOHYDRATE_METABOLIC_PROCESS	0.00028208
GOBP_CHEMICAL_HOMEOSTASIS	0.00028318
GOBP_REGULATION_OF_ORGANELLE_ORGANIZATION	0.00028453
GOBP_FATTY_ACID_METABOLIC_PROCESS	0.00028589
GOBP_ORGANIC_ACID_BIOSYNTHETIC_PROCESS	0.00028781
GOBP_CELLULAR_PROCESS_INVOLVED_IN_REPRODUCTION_IN_MULTICELLULAR_ORGANISM	0.00029543
GOBP_CELLULAR_RESPONSE_TO_UNFOLDED_PROTEIN	0.00030672
GOBP_REGULATION_OF_PROTEOLYSIS	0.00032191
GOBP_BIOLOGICAL_PROCESS_INVOLVED_IN_INTERACTION_WITH_HOST	0.00032287
GOBP_POST_TRANSCRIPTIONAL_REGULATION_OF_GENE_EXPRESSION	0.00033539
GOBP_NEURON_PROJECTION_EXTENSION	0.00033696
GOBP_POSITIVE_REGULATION_OF_T_CELL_PROLIFERATION	0.00034833
GOBP_INTERLEUKIN_6_PRODUCTION	0.00035193
GOBP_MICROTUBULE_CYTOSKELETON_ORGANIZATION_INVOLVED_IN_MITOSIS	0.0003525
GOBP_CELLULAR_RESPONSE_TO_STARVATION	0.0003528
GOBP_HETEROCYCLE_CATABOLIC_PROCESS	0.00037383
GOBP_RESPONSE_TO_TRANSFORMING_GROWTH_FACTOR_BETA	0.00037578
GOBP_DNA_TEMPLATED_TRANSCRIPTION_INITIATION	0.00038385
GOBP_SYNAPSE_ORGANIZATION	0.00042681
GOBP_ESTABLISHMENT_OF_CELL_POLARITY	0.00043818
GOBP_NUCLEOBASE_CONTAINING_COMPOUND_TRANSPORT	0.00044138
GOBP_MIRNA_METABOLIC_PROCESS	0.00044631
GOBP_ACTIN_FILAMENT_BASED_PROCESS	0.00045292
GOBP_POSITIVE_REGULATION_OF_CELL_GROWTH	0.00047076

GOBP_RESPONSE_TO_ORGANIC_CYCLIC_COMPOUND	0.00047861
GOBP_CELL_CYCLE_G1_S_PHASE_TRANSITION	0.00049216
GOBP_T_CELL_PROLIFERATION	0.00052325
GOBP_G_PROTEIN_COUPLED_RECEPTOR_SIGNALING_PATHWAY	0.00052388
GOBP_HEART_DEVELOPMENT	0.00052629
GOBP_FAT_CELL_DIFFERENTIATION	0.00054387
GOBP_ESTABLISHMENT_OF_ORGANELLE_LOCALIZATION	0.00060815
GOBP_REGULATION_OF_LEUKOCYTE_MEDIATED_IMMUNITY	0.00061084
GOBP_POSITIVE_REGULATION_OF_CYTOSKELETON_ORGANIZATION	0.00061392
GOBP_POSITIVE_REGULATION_OF_CELLULAR_COMPONENT_ORGANIZATION	0.0006238
GOBP_RESPONSE_TO_TYPE_II_INTERFERON	0.00064778
GOBP_NON_CANONICAL_NF_KAPPAB_SIGNAL_TRANSDUCTION	0.00064863
GOBP_RECOMBINATIONAL_REPAIR	0.00067242
GOBP_ANTIGEN_PROCESSING_AND_PRESENTATION	0.00067506
GOBP_RESPONSE_TO_MOLECULE_OF_BACTERIAL_ORIGIN	0.00072878
GOBP_POSITIVE_REGULATION_OF_PROTEIN_MODIFICATION_BY_SMALL_PROTEIN_CONJUGATION_OR_REMOVAL	0.00072973
GOBP_CANONICAL_NF_KAPPAB_SIGNAL_TRANSDUCTION	0.00073316
GOBP_MUSCLE_CELL_PROLIFERATION	0.00074463
GOBP_GASTRULATION	0.00074914
GOBP_PROTEIN_LOCALIZATION_TO_ENDOPLASMIC_RETICULUM	0.00075228
GOBP_MEMBRANE_DOCKING	0.00079299
GOBP_SMALL_GTPASE_MEDIATED_SIGNAL_TRANSDUCTION	0.00083264
GOBP_CHROMATIN_REMODELING	0.00092685
GOBP_REGULATION_OF_SMALL_GTPASE_MEDIATED_SIGNAL_TRANSDUCTION	0.00095844
GOBP_TRANSCRIPTION_INITIATION_AT_RNA_POLYMERASE_II_PROMOTER	0.00096198
GOBP_BIOLOGICAL_PROCESS_INVOLVED_IN_SYMBIOTIC_INTERACTION	0.00098039
GOBP_CALCIUM_ION_HOMEOSTASIS	0.00100754
GOBP_RNA_CATABOLIC_PROCESS	0.00106373
GOBP_REGULATION_OF_CELL_CYCLE_PROCESS	0.00107065
GOBP_GOLGI_VESICLE_TRANSPORT	0.00112505
GOBP_CELL_KILLING	0.00112767
GOBP_MUSCLE_CELL_DIFFERENTIATION	0.0011587
GOBP_POSITIVE_REGULATION_OF_HYDROLASE_ACTIVITY	0.00118574
GOBP_CELL_SUBSTRATE_ADHESION	0.00119439
GOBP_PROTEIN_MODIFICATION_BY_SMALL_PROTEIN_CONJUGATION	0.0012693
GOBP_RESPONSE_TO_XENOBIOTIC_STIMULUS	0.00127271
GOBP_NEGATIVE_REGULATION_OF_GENE_EXPRESSION	0.00127371
GOBP_HEART_MORPHOGENESIS	0.00130595
GOBP_PROTEIN_LOCALIZATION_TO_ORGANELLE	0.00133735
GOBP_NEGATIVE_REGULATION_OF_CELLULAR_COMPONENT_ORGANIZATION	0.00134096
GOBP_POSITIVE_REGULATION_OF_LEUKOCYTE_PROLIFERATION	0.00139919
GOBP_RNA_3_END_PROCESSING	0.00144256
GOBP_REGULATION_OF_STRESS_ACTIVATED_PROTEIN_KINASE_SIGNALING_CASCADE	0.001446
GOBP_HEART_PROCESS	0.00149816
GOBP_REGULATION_OF_PROTEIN_POLYMERIZATION	0.0015176
GOBP_TELOMERE_MAINTENANCE	0.00154414
GOBP_OXIDATIVE_PHOSPHORYLATION	0.00169974
GOBP_BLOOD_VESSEL_ENDOTHELIAL_CELL_MIGRATION	0.0017223
GOBP_ORGANELLE_FISSION	0.00186727
GOBP_REGULATION_OF_MEMBRANE_POTENTIAL	0.00192882
GOBP_REGULATION_OF_CATALYTIC_ACTIVITY	0.00195402
GOBP_PRODUCTION_OF_MOLECULAR_MEDIATOR_OF_IMMUNE_RESPONSE	0.00196051
GOBP_TISSUE_HOMEOSTASIS	0.00199443
GOBP_REGULATION_OF_SYNAPSE_STRUCTURE_OR_ACTIVITY	0.00200939
GOBP_REGULATION_OF_SYNAPTIC_PLASTICITY	0.00207688
GOBP_CENTRAL_NERVOUS_SYSTEM_DEVELOPMENT	0.00209226
GOBP_T_CELL_MEDIATED_IMMUNITY	0.00214047
GOBP_CHROMOSOME_LOCALIZATION	0.00234994
GOBP_REGULATION_OF_REACTIVE_OXYGEN_SPECIES_METABOLIC_PROCESS	0.0023905
GOBP_VIRAL_GENE_EXPRESSION	0.00245883
GOBP_REGULATION_OF_MACROAUTOPHAGY	0.00258543
GOBP_NEGATIVE_REGULATION_OF_GENE_EXPRESSION_EPIGENETIC	0.00274725
GOBP_MONOCARBOXYLIC_ACID_METABOLIC_PROCESS	0.00278434
GOBP_MULTICELLULAR_ORGANISMAL_LEVEL_HOMEOSTASIS	0.00279253

GOBP_RENAL_SYSTEM_DEVELOPMENT	0.00296599
GOBP_NEGATIVE_REGULATION_OF_PHOSPHORYLATION	0.00298598
GOBP_PEPTIDYL_AMINO_ACID_MODIFICATION	0.00308464
GOBP_RESPONSE_TO_HORMONE	0.00314224
GOBP_HETEROCHROMATIN_ORGANIZATION	0.00317382
GOBP_DEVELOPMENTAL_CELL_GROWTH	0.00325377
GOBP_CELLULAR_RESPONSE_TO_STEROID_HORMONE_STIMULUS	0.00325406
GOBP_MONOSACCHARIDE_METABOLIC_PROCESS	0.00326614
GOBP_CIRCADIAN_RHYTHM	0.0033317
GOBP_CELL_ACTIVATION_INVOLVED_IN_IMMUNE_RESPONSE	0.00346453
GOBP_REGULATION_OF_HORMONE_LEVELS	0.00348231
GOBP_ATP_METABOLIC_PROCESS	0.00356822
GOBP_CYTOKINE_MEDIATED_SIGNALING_PATHWAY	0.00364765
GOBP_REGULATION_OF_MITOTIC_CELL_CYCLE	0.00372779
GOBP_POSITIVE_REGULATION_OF_PROTEIN_UBIQUITINATION	0.00373144
GOBP_ATP_SYNTHESIS_COUPLED_ELECTRON_TRANSPORT	0.00385416
GOBP_MICROTUBULE_BASED_PROCESS	0.00387658
GOBP_RESPONSE_TO_TEMPERATURE_STIMULUS	0.00391749
GOBP_MICROTUBULE_BASED_MOVEMENT	0.00410452
GOBP_ORGANOPHOSPHATE_BIOSYNTHETIC_PROCESS	0.00432178
GOBP_SMALL_MOLECULE_METABOLIC_PROCESS	0.00437992
GOBP_JNK_CASCADE	0.00447327
GOBP_RNA_DESTABILIZATION	0.00447502
GOBP_POSITIVE_REGULATION_OF_ORGANELLE_ORGANIZATION	0.00477447
GOBP_PROTEIN_MATURATION	0.00522881
GOBP_VESICLE_TARGETING	0.00535323
GOBP_NEGATIVE_REGULATION_OF_PROTEIN_CONTAINING_COMPLEX_ASSEMBLY	0.00556979
GOBP_REGULATION_OF_PROTEIN_SECRETION	0.00562651
GOBP_MUSCLE_STRUCTURE_DEVELOPMENT	0.0058007
GOBP_NEGATIVE_REGULATION_OF_MAPK_CASCADE	0.00587209
GOBP_ELECTRON_TRANSPORT_CHAIN	0.00590194
GOBP_REGULATION_OF_NEURON_PROJECTION_DEVELOPMENT	0.00594656
GOBP_NUCLEAR_EXPORT	0.00610679
GOBP_CYTOKINESIS	0.0064225
GOBP_TOR_SIGNALING	0.00643813
GOBP_CIRCULATORY_SYSTEM_PROCESS	0.00667021
GOBP_PROTEIN_HOMOOLIGOMERIZATION	0.00667297
GOBP_REGULATION_OF_ACTIN_FILAMENT_LENGTH	0.00712784
GOBP_CD4_POSITIVE_ALPHA_BETA_T_CELL_ACTIVATION	0.00730994
GOBP_ORGANELLE_DISASSEMBLY	0.00738649
GOBP_SMOOTH_MUSCLE_CELL_PROLIFERATION	0.00751483
GOBP_NEGATIVE_REGULATION_OF_DNA_BINDING_TRANSCRIPTION_FACTOR_ACTIVITY	0.00812726
GOBP_CELL_MORPHOGENESIS_INVOLVED_IN_NEURON_DIFFERENTIATION	0.00831395
GOBP_SYNAPTIC_SIGNALING	0.00845914
GOBP_ESTABLISHMENT_OR_MAINTENANCE_OF_CELL_POLARITY	0.00868576
GOBP_TELOMERE_ORGANIZATION	0.00898803
GOBP_MRNA_METABOLIC_PROCESS	0.00928428
GOBP_REGULATION_OF_CELL_SHAPE	0.00963909
GOBP_TRANSFORMING_GROWTH_FACTOR_BETA_RECEPTOR_SIGNALING_PATHWAY	0.00983523
GOBP_TUMOR_NECROSIS_FACTOR_MEDIATED_SIGNALING_PATHWAY	0.00989363
GOBP_LYMPHOCYTE_ACTIVATION_INVOLVED_IN_IMMUNE_RESPONSE	0.01016726
GOBP_REGULATION_OF_MITOTIC_CELL_CYCLE_PHASE_TRANSITION	0.01063651
GOBP_POSITIVE_REGULATION_OF_PROTEASOMAL_PROTEIN_CATABOLIC_PROCESS	0.01110731
GOBP_NEGATIVE_REGULATION_OF_KINASE_ACTIVITY	0.01179019
GOBP_LEUKOCYTE_HOMEOSTASIS	0.01205845
GOBP_POSITIVE_REGULATION_OF_VIRAL_PROCESS	0.01208171
GOBP_AXON_DEVELOPMENT	0.01295564
GOBP_NEGATIVE_REGULATION_OF_CELL_CELL_ADHESION	0.01365737
GOBP_VESICLE_ORGANIZATION	0.01402023
GOBP_NEGATIVE_REGULATION_OF_TRANSFERASE_ACTIVITY	0.01454318
GOBP_REGULATION_OF_HYDROLASE_ACTIVITY	0.01485243
GOBP_REGULATION_OF_WNT_SIGNALING_PATHWAY	0.01499213
GOBP_REGULATION_OF_TRANSPORTER_ACTIVITY	0.01520224
GOBP_POSITIVE_REGULATION_OF_PROTEOLYSIS_INVOLVED_IN_PROTEIN_CATABOLIC_PROCESS	0.01600339

GOBP_ACTOMYOSIN_STRUCTURE_ORGANIZATION	0.01600667
GOBP_RESPONSE_TO_HYDROGEN_PEROXIDE	0.01658229
GOBP_MEMBRANE_FUSION	0.01685976
GOBP_MAINTENANCE_OF_LOCATION	0.01691089
GOBP_REGENERATION	0.01703773
GOBP_MYELOID_LEUKOCYTE_ACTIVATION	0.01717886
GOBP_POSITIVE_REGULATION_OF_LEUKOCYTE_MEDIATED_IMMUNITY	0.01751868
GOBP_NEGATIVE_REGULATION_OF_LEUKOCYTE_CELL_CELL_ADHESION	0.01782463
GOBP_ACTIN_FILAMENT_ORGANIZATION	0.01813039
GOBP_RESPONSE_TO_METAL_ION	0.01861767
GOBP_CYTOSKELETON_DEPENDENT_INTRACELLULAR_TRANSPORT	0.01895273
GOBP_VACUOLE_ORGANIZATION	0.01917911
GOBP_REGULATION_OF_INFLAMMATORY_RESPONSE	0.01977231
GOBP_DEVELOPMENTAL_PROCESS_INVOLVED_IN_REPRODUCTION	0.01986166
GOBP_NEGATIVE_REGULATION_OF_TRANSMEMBRANE_RECEPTOR_PROTEIN_SERINE_THREONINE_ KINASE_SIGNALING_PATHWAY	0.02059509
GOBP_REGULATION_OF_CELL_CYCLE_G2_M_PHASE_TRANSITION	0.02120665
GOBP_CELLULAR_COMPONENT_MORPHOGENESIS	0.02149994
GOBP_REGULATION_OF_LYMPHOCYTE_MEDIATED_IMMUNITY	0.02182497
GOBP_ACTIN_FILAMENT_BUNDLE_ORGANIZATION	0.02287514
GOBP_STRESS_ACTIVATED_PROTEIN_KINASE_SIGNALING_CASCADE	0.02376755
GOBP_REGULATION_OF_CELLULAR_COMPONENT_BIOGENESIS	0.02540733
GOBP_RESPIRATORY_ELECTRON_TRANSPORT_CHAIN	0.02579576
GOBP_CELL_PART_MORPHOGENESIS	0.0263307
GOBP_REGULATION_OF_PRODUCTION_OF_MOLECULAR_MEDIATOR_OF_IMMUNE_RESPONSE	0.0265701
GOBP_TUMOR_NECROSIS_FACTOR_SUPERFAMILY_CYTOKINE_PRODUCTION	0.02669967
GOBP_CALCIUM_ION_TRANSPORT	0.02699859
GOBP_MITOTIC_CELL_CYCLE_PROCESS	0.02725029
GOBP_REGULATION_OF_CHROMOSOME_ORGANIZATION	0.02758126
GOBP_B_CELL_DIFFERENTIATION	0.02832955
GOBP_ENDOCYTIC_RECYCLING	0.03152139
GOBP_PROTEIN_POLYMERIZATION	0.03290928
GOBP_NEGATIVE_REGULATION_OF_LYMPHOCYTE_ACTIVATION	0.03304913
GOBP_POSITIVE_REGULATION_OF_ADAPTIVE_IMMUNE_RESPONSE	0.03351106
GOBP_RESPONSE_TO_TUMOR_NECROSIS_FACTOR	0.03473519
GOBP_POSITIVE_REGULATION_OF_CATALYTIC_ACTIVITY	0.03479531
GOBP_NEGATIVE_REGULATION_OF_PROTEIN_LOCALIZATION	0.03571387
GOBP_ORGANELLE_FUSION	0.03581516
GOBP_PROTEIN_FOLDING	0.03593937
GOBP_RESPONSE_TO_ENDOGENOUS_STIMULUS	0.03646789
GOBP_CELLULAR_RESPONSE_TO_HORMONE_STIMULUS	0.03836989
GOBP_REGULATION_OF_CELLULAR_RESPONSE_TO_TRANSFORMING_GROWTH_FACTOR_BETA_STIMULUS	0.03842632
GOBP_EPITHELIAL_TUBE_MORPHOGENESIS	0.03921227
GOBP_REGULATION_OF_NON_CANONICAL_NF_KAPPAB_SIGNAL_TRANSDUCTION	0.03964659
GOBP_NEGATIVE_REGULATION_OF_MITOTIC_CELL_CYCLE	0.04129955
GOBP_PROTON_MOTIVE_FORCE_DRIVEN_ATP_SYNTHESIS	0.04201254
GOBP_MICROTUBULE_POLYMERIZATION	0.04281381
GOBP_POSITIVE_REGULATION_OF_PROTEOLYSIS	0.04284042
GOBP_MUSCLE_TISSUE_DEVELOPMENT	0.04500896
GOBP_STEROID_METABOLIC_PROCESS	0.04513123
GOBP_SELECTIVE_AUTOPHAGY	0.04517676
GOBP_RESPONSE_TO_ALCOHOL	0.04580898
GOBP_EXOCYTOSIS	0.04630608
GOBP_LIPID_METABOLIC_PROCESS	0.04755122
GOBP_NUCLEOSIDE_TRIPHOSPHATE_METABOLIC_PROCESS	0.04799637
GOBP_SKIN_DEVELOPMENT	0.04833807
GOBP_MYELOID_LEUKOCYTE_DIFFERENTIATION	0.04915658
GOBP_TRNA_METABOLIC_PROCESS	0.05013002
GOBP_REGULATORY_NCRNA_MEDIATED_GENE_SILENCING	0.05016146
GOBP_POSITIVE_REGULATION_OF_TRANSFERASE_ACTIVITY	0.0511406
GOBP_NEGATIVE_REGULATION_OF_CELL_ADHESION	0.05230543
GOBP_POSITIVE_REGULATION_OF_INTRACELLULAR_SIGNAL_TRANSDUCTION	0.05475069
GOBP_PHOSPHATIDYLINOSITOL_BIOSYNTHETIC_PROCESS	0.05476842
GOBP_REGULATION_OF_TRANSCRIPTION_ELONGATION_BY_RNA_POLYMERASE_II	0.06565539

GOBP_POSITIVE_REGULATION_OF_CELL_CYCLE	0.06601886
GOBP_RESPONSE_TO_NITROGEN_COMPOUND	0.06642185
GOBP_MRNA_CATABOLIC_PROCESS	0.06642458
GOBP_DENDRITE_MORPHOGENESIS	0.0668873
GOBP_MUSCLE_CONTRACTION	0.06971563
GOBP_PHAGOCYTOSIS	0.07545647
GOBP_GLUCOSE_METABOLIC_PROCESS	0.07609243
GOBP_VESICLE_MEDIATED_TRANSPORT_IN_SYNAPSE	0.07618605
GOBP_POSITIVE_REGULATION_OF_CELLULAR_COMPONENT_BIOGENESIS	0.07805517
GOBP_POSITIVE_REGULATION_OF_APOPTOTIC_SIGNALING_PATHWAY	0.07838556
GOBP_ORGANELLE_MEMBRANE_FUSION	0.08162099
GOBP_MAINTENANCE_OF_PROTEIN_LOCATION	0.08211418
GOBP_PLASMA_MEMBRANE_ORGANIZATION	0.08485585
GOBP_PROTEIN_CONTAINING_COMPLEX_LOCALIZATION	0.08647785
GOBP_CELLULAR_RESPONSE_TO_PEPTIDE_HORMONE_STIMULUS	0.08835772
GOBP_NEGATIVE_REGULATION_OF_DEVELOPMENTAL_PROCESS	0.09223286
GOBP_MICROTUBULE_CYTOSKELETON_ORGANIZATION	0.09232447
GOBP_REGULATION_OF_GTPASE_ACTIVITY	0.09285576
GOBP_NEGATIVE_REGULATION_OF_MOLECULAR_FUNCTION	0.09641162
GOBP_REGULATION_OF_TRANSLATIONAL_INITIATION	0.09775888
GOBP_POSITIVE_REGULATION_OF_BINDING	0.09838582
GOBP_CALCIUM_ION_TRANSMEMBRANE_TRANSPORT	0.10410149
GOBP_T_CELL_DIFFERENTIATION_IN_THYMUS	0.10667946
GOBP_RESPONSE_TO_ACID_CHEMICAL	0.10791288
GOBP_NEGATIVE_REGULATION_OF_CELL_ACTIVATION	0.10911835
GOBP_REGULATION_OF_TRANS_SYNAPTIC_SIGNALING	0.1102871
GOBP_PYRUVATE_METABOLIC_PROCESS	0.11098197
GOBP_REGULATION_OF_AMIDE_METABOLIC_PROCESS	0.11439604
GOBP_CELLULAR_RESPONSE_TO_DECREASED_OXYGEN_LEVELS	0.12101489
GOBP_HORMONE_TRANSPORT	0.12409767
GOBP_RIBOSE_PHOSPHATE_BIOSYNTHETIC_PROCESS	0.12639305
GOBP_ORGANOPHOSPHATE_METABOLIC_PROCESS	0.13137859
GOBP_RESPONSE_TO_MECHANICAL_STIMULUS	0.13250675
GOBP_DNA_TEMPLATED_DNA_REPLICATION	0.13318473
GOBP_REGULATION_OF_PEPTIDASE_ACTIVITY	0.13549754
GOBP_TEMPERATURE_HOMEOSTASIS	0.1377298
GOBP_INTRACELLULAR_GLUCOSE_HOMEOSTASIS	0.14575482
GOBP_POSITIVE_REGULATION_OF_SECRETION	0.14798504
GOBP_ATP_BIOSYNTHETIC_PROCESS	0.14834663
GOBP_POSITIVE_REGULATION_OF_LYMPHOCYTE_DIFFERENTIATION	0.15269583
GOBP_STEROID_HORMONE_MEDIATED_SIGNALING_PATHWAY	0.15504447
GOBP_RESPONSE_TO_RADIATION	0.15677769
GOBP_PROTEIN_LOCALIZATION_TO_EXTRACELLULAR_REGION	0.15686219
GOBP_REGULATION_OF_MRNA_SPLICING_VIA_SPLICEOSOME	0.16464291
GOBP_SIGNAL_TRANSDUCTION_IN_RESPONSE_TO_DNA_DAMAGE	0.16684931
GOBP_STRIATED_MUSCLE_TISSUE_DEVELOPMENT	0.16782325
GOBP_REGULATION_OF_TRANSFERASE_ACTIVITY	0.16854031
GOBP_NEGATIVE_REGULATION_OF_IMMUNE_SYSTEM_PROCESS	0.17162639
GOBP_MICROTUBULE_POLYMERIZATION_OR_DEPOLYMERIZATION	0.17273725
GOBP_REGULATION_OF_CELL_JUNCTION_ASSEMBLY	0.18268991
GOBP_POSITIVE_REGULATION_OF_MRNA_METABOLIC_PROCESS	0.18480376
GOBP_REGULATION_OF_BINDING	0.18503384
GOBP_MULTICELLULAR_ORGANISM_REPRODUCTION	0.18528486
GOBP_GAMETE_GENERATION	0.19353917
GOBP_CELL_CYCLE_PROCESS	0.19451862
GOBP_VESICLE_BUDDING_FROM_MEMBRANE	0.20138227
GOBP_DNA_INTEGRITY_CHECKPOINT_SIGNALING	0.20446021
GOBP_CELL_CYCLE	0.20483426
GOBP_REGULATION_OF_EPITHELIAL_CELL_PROLIFERATION	0.20838288
GOBP_RESPONSE_TO_TOPOLOGICALLY_INCORRECT_PROTEIN	0.21173222
GOBP_REGULATION_OF_CELL_SIZE	0.21700888
GOBP_POSITIVE_REGULATION_OF_LYMPHOCYTE_MEDIATED_IMMUNITY	0.21799793
GOBP_ORGANIC_HYDROXY_COMPOUND_BIOSYNTHETIC_PROCESS	0.22106389
GOBP_MITOTIC_CELL_CYCLE	0.2251848

GOBP_EPIGENETIC_REGULATION_OF_GENE_EXPRESSION	0.23150895
GOBP_CALCIUM_ION_TRANSMEMBRANE_IMPORT_INTO_CYTOSOL	0.23602995
GOBP_RESPONSE_TO_UV	0.24155024
GOBP_RESPONSE_TO_PEPTIDE_HORMONE	0.24649193
GOBP_NEGATIVE_REGULATION_OF_CELLULAR_CATABOLIC_PROCESS	0.24979321
GOBP_REGULATION_OF_ESTABLISHMENT_OF_PROTEIN_LOCALIZATION	0.25197922
GOBP_NEGATIVE_REGULATION_OF_TRANSPORT	0.25204734
GOBP_CELLULAR_RESPONSE_TO_INSULIN_STIMULUS	0.25435928
GOBP_REGULATION_OF_ORGANELLE_ASSEMBLY	0.25439877
GOBP_CELL_DIVISION	0.25513539
GOBP_CELLULAR_LIPID_CATABOLIC_PROCESS	0.25617339
GOBP_STRIATED_MUSCLE_CELL_DIFFERENTIATION	0.26092928
GOBP_RESPONSE_TO_LIGHT_STIMULUS	0.26176068
GOBP_POSITIVE_REGULATION_OF_PROTEIN_CATABOLIC_PROCESS	0.26539952
GOBP_RHYTHMIC_PROCESS	0.27237606
GOBP_BEHAVIOR	0.27845552
GOBP_PEPTIDYL_LYSINE_MODIFICATION	0.28045209
GOBP_TYPE_I_INTERFERON_PRODUCTION	0.28290386
GOBP_REGULATION_OF_CALCIUM_ION_TRANSPORT	0.28923723
GOBP_BIOLOGICAL_PROCESS_INVOLVED_IN_INTERACTION_WITH_SYMBIONT	0.28992521
GOBP_SIGNAL_RELEASE	0.29286207
GOBP_POSITIVE_REGULATION_OF_GTPASE_ACTIVITY	0.31116648
GOBP_NEGATIVE_REGULATION_OF_MITOTIC_CELL_CYCLE_PHASE_TRANSITION	0.31344637
GOBP_RNA_METHYLATION	0.32641328
GOBP_REGULATION_OF_NUCLEOCYTOPLASMIC_TRANSPORT	0.33060639
GOBP_MAINTENANCE_OF_LOCATION_IN_CELL	0.33129434
GOBP_POSITIVE_REGULATION_OF_INTRACELLULAR_TRANSPORT	0.33559751
GOBP_PEPTIDE_TRANSPORT	0.34446966
GOBP_POSITIVE_REGULATION_OF_CELL_CYCLE_PROCESS	0.35045435
GOBP_POSITIVE_REGULATION_OF_TRANSLATION	0.35225615
GOBP_CELLULAR_RESPONSE_TO_OXIDATIVE_STRESS	0.35538181
GOBP_ORGANELLE_TRANSPORT_ALONG_MICROTUBULE	0.36185088
GOBP_CELLULAR_RESPONSE_TO_PEPTIDE	0.38183008
GOBP_PROTEIN_K48_LINKED_UBIQUITINATION	0.38583772
GOBP_ENDOPLASMIC_RETICULUM_TO_GOLGI_VESICLE_MEDIATED_TRANSPORT	0.39123978
GOBP_RECEPTOR_SIGNALING_PATHWAY_VIA_STAT	0.39518286
GOBP_CELLULAR_LIPID_METABOLIC_PROCESS	0.39705263
GOBP_PROTEIN_POLYUBIQUITINATION	0.39806323
GOBP_DNA_REPLICATION	0.4036234
GOBP_ENDOPLASMIC_RETICULUM_UNFOLDED_PROTEIN_RESPONSE	0.40433321
GOBP_DEVELOPMENTAL_GROWTH_INVOLVED_IN_MORPHOGENESIS	0.41011589
GOBP_RESPONSE_TO_INSULIN	0.419388
GOBP_REGULATION_OF_CALCIUM_ION_TRANSMEMBRANE_TRANSPORT	0.43272207
GOBP_RESPONSE_TO_OXIDATIVE_STRESS	0.43542112
GOBP_CELLULAR_RESPONSE_TO_BIOTIC_STIMULUS	0.43589235
GOBP_REGULATION_OF_SYSTEM_PROCESS	0.43631399
GOBP_PLACENTA_DEVELOPMENT	0.47709177
GOBP_ORGANIC_ACID_CATABOLIC_PROCESS	0.48072778
GOBP_AMIDE_TRANSPORT	0.48100777
GOBP_POSITIVE_REGULATION_OF_AMIDE_METABOLIC_PROCESS	0.48316222
GOBP_CELLULAR_RESPONSE_TO_TOPOLOGICALLY_INCORRECT_PROTEIN	0.48812984
GOBP_REGULATION_OF_CELL_PROJECTION_ASSEMBLY	0.49143554
GOBP_NUCLEAR_TRANSCRIBED_MRNA_CATABOLIC_PROCESS_DEADENYLATION_DEPENDENT_DECAY	0.50896959
GOBP_REGULATION_OF_MRNA_CATABOLIC_PROCESS	0.5118387
GOBP_NEGATIVE_REGULATION_OF_INFLAMMATORY_RESPONSE	0.51481311
GOBP_POSITIVE_REGULATION_OF_PROGRAMMED_CELL_DEATH	0.51675506
GOBP_NUCLEOSOME_ORGANIZATION	0.5168922
GOBP_NEGATIVE_REGULATION_OF_PHOSPHORUS_METABOLIC_PROCESS	0.51858939
GOBP_MALE_GAMETE_GENERATION	0.52269462
GOBP_LYMPHOCYTE_APOPTOTIC_PROCESS	0.52351724
GOBP_RESPONSE_TO_OXYGEN_LEVELS	0.52420085
GOBP_REGULATION_OF_TELOMERE_MAINTENANCE	0.52872915
GOBP_NUCLEAR_TRANSCRIBED_MRNA_CATABOLIC_PROCESS	0.52950452
GOBP_RESPONSE_TO_PEPTIDE	0.52981904

GOBP_REGULATION_OF_VASCULATURE_DEVELOPMENT	0.53093992
GOBP_REGULATION_OF_CELLULAR_COMPONENT_SIZE	0.53157577
GOBP_MUSCLE_SYSTEM_PROCESS	0.53787437
GOBP_T_CELL_RECEPTOR_SIGNALING_PATHWAY	0.53910339
GOBP_REGULATION_OF_DNA_REPLICATION	0.54763855
GOBP_GLYCOSYLATION	0.54888845
GOBP_ANATOMICAL_STRUCTURE_MATURATION	0.55052221
GOBP_NEURAL_TUBE_DEVELOPMENT	0.55239047
GOBP_STRESS_FIBER_ASSEMBLY	0.55331707
GOBP_POSITIVE_REGULATION_OF_ERK1_AND_ERK2_CASCADE	0.56237896
GOBP_REGULATION_OF_RAS_PROTEIN_SIGNAL_TRANSDUCTION	0.58643361
GOBP_CELLULAR_RESPONSE_TO_OXYGEN_LEVELS	0.58937068
GOBP_MITOTIC_CELL_CYCLE_CHECKPOINT_SIGNALING	0.59045813
GOBP_NEGATIVE_REGULATION_OF_LOCOMOTION	0.59288438
GOBP_MACROMOLECULE_METHYLATION	0.59312758
GOBP_NEGATIVE_REGULATION_OF_MRNA_METABOLIC_PROCESS	0.59555671
GOBP_REPRODUCTION	0.59781147
GOBP_CILIUM_ORGANIZATION	0.59869642
GOBP_LIPOPROTEIN_METABOLIC_PROCESS	0.61203065
GOBP_REACTIVE_OXYGEN_SPECIES_METABOLIC_PROCESS	0.62569319
GOBP_GOLGI_ORGANIZATION	0.63165065
GOBP_IMMUNE_SYSTEM_DEVELOPMENT	0.63689476
GOBP_PROTEIN_PROCESSING	0.64680256
GOBP_PROTEIN_DEPHOSPHORYLATION	0.64753705
GOBP_MITOCHONDRIAL_RESPIRATORY_CHAIN_COMPLEX_ASSEMBLY	0.6578649
GOBP_REGULATION_OF_CYSTEINE_TYPE_ENDOPEPTIDASE_ACTIVITY_INVOLVED_IN_APOPTOTIC_PROCESS	0.65888608
GOBP_MICROTUBULE_ORGANIZING_CENTER_ORGANIZATION	0.66220182
GOBP_REGULATION_OF_CELL_MATRIX_ADHESION	0.66431865
GOBP_REGULATION_OF_SECRETION	0.66692701
GOBP_PROTEIN_LOCALIZATION_TO_VACUOLE	0.67553254
GOBP_NEGATIVE_REGULATION_OF_BINDING	0.6778161
GOBP_GLIOGENESIS	0.68382578
GOBP_MAPK_CASCADE	0.68463386
GOBP_REGULATION_OF_LEUKOCYTE_APOPTOTIC_PROCESS	0.69165042
GOBP_TRANSMEMBRANE_RECEPTOR_PROTEIN_TYROSINE_KINASE_SIGNALING_PATHWAY	0.69398927
GOBP_RESPONSE_TO_INORGANIC_SUBSTANCE	0.69679994
GOBP_POSITIVE_REGULATION_OF_NERVOUS_SYSTEM_DEVELOPMENT	0.69834722
GOBP_POSITIVE_REGULATION_OF_CANONICAL_NF_KAPPAB_SIGNAL_TRANSDUCTION	0.71744003
GOBP_DEVELOPMENTAL_GROWTH	0.7178368
GOBP_GLIAL_CELL_DIFFERENTIATION	0.7183447
GOBP_MORPHOGENESIS_OF_AN_EPITHELIUM	0.72047541
GOBP_ENZYME_LINKED_RECEPTOR_PROTEIN_SIGNALING_PATHWAY	0.72097626
GOBP_REGULATION_OF_CHROMOSOME_SEGREGATION	0.72836148
GOBP_TUBE_FORMATION	0.73410022
GOBP_DNA_TEMPLATED_TRANSCRIPTION_ELONGATION	0.74897761
GOBP_CELL_PROJECTION_ORGANIZATION	0.75201039
GOBP_REPRODUCTIVE_SYSTEM_DEVELOPMENT	0.7606793
GOBP_POSITIVE_REGULATION_OF_NEUROGENESIS	0.76346544
GOBP_GLAND_DEVELOPMENT	0.77628267
GOBP_POSITIVE_REGULATION_OF_PROTEIN_LOCALIZATION	0.77735575
GOBP_REGULATION_OF_CYSTEINE_TYPE_ENDOPEPTIDASE_ACTIVITY	0.77770842
GOBP_NEGATIVE_REGULATION_OF_CELL_CYCLE	0.77827332
GOBP_ACTIN_POLYMERIZATION_OR_DEPOLYMERIZATION	0.78606677
GOBP_MULTI_MULTICELLULAR_ORGANISM_PROCESS	0.79470113
GOBP_PHOSPHATIDYLINOSITOL_METABOLIC_PROCESS	0.79636639
GOBP_GLYCEROPHOSPHOLIPID_BIOSYNTHETIC_PROCESS	0.79923203
GOBP_GLYCEROPHOSPHOLIPID_METABOLIC_PROCESS	0.79926588
GOBP_NUCLEOTIDE_EXCISION_REPAIR	0.80381647
GOBP_INSULIN_SECRETION	0.8075811
GOBP_MUSCLE_ORGAN_DEVELOPMENT	0.81117286
GOBP_SMALL_MOLECULE_CATABOLIC_PROCESS	0.81579067
GOBP_CELL_CYCLE_G2_M_PHASE_TRANSITION	0.81857171
GOBP_CELLULAR_RESPONSE_TO_ABIOTIC_STIMULUS	0.82154666
GOBP_REGULATION_OF_CELL_CYCLE_PHASE_TRANSITION	0.82890077

GOBP_DEVELOPMENT_OF_PRIMARY_SEXUAL_CHARACTERISTICS	0.82905817
GOBP_NUCLEAR_TRANSPORT	0.8295663
GOBP_REGULATION_OF_DEVELOPMENTAL_GROWTH	0.8332057
GOBP_NEGATIVE_REGULATION_OF_IMMUNE_EFFECTOR_PROCESS	0.83492963
GOBP_OSTEOBLAST_DIFFERENTIATION	0.83570887
GOBP_NEGATIVE_REGULATION_OF_CYTOKINE_PRODUCTION	0.83816851
GOBP_REGULATION_OF_NEURON_APOPTOTIC_PROCESS	0.83938478
GOBP_CELLULAR_RESPONSE_TO_EXTRACELLULAR_STIMULUS	0.8409113
GOBP_NEGATIVE_REGULATION_OF_CELL_POPULATION_PROLIFERATION	0.84124307
GOBP_SKELETAL_MUSCLE_ORGAN_DEVELOPMENT	0.84418493
GOBP_REGULATION_OF_LIPID_BIOSYNTHETIC_PROCESS	0.84652253
GOBP_POSITIVE_REGULATION_OF_UBIQUITIN_DEPENDENT_PROTEIN_CATABOLIC_PROCESS	0.84686845
GOBP_NUCLEOSIDE_TRIPHOSPHATE_BIOSYNTHETIC_PROCESS	0.84774652
GOBP_RNA_MODIFICATION	0.84803733
GOBP_ERBB_SIGNALING_PATHWAY	0.84906391
GOBP_IMPORT_INTO_NUCLEUS	0.8540282
GOBP_EPIDERMIS_DEVELOPMENT	0.86450081
GOBP_RESPONSE_TO_STARVATION	0.86954671
GOBP_METHYLATION	0.88089516
GOBP_HEPATICOBILIARY_SYSTEM_DEVELOPMENT	0.8829022
GOBP_RESPONSE_TO_CARBOHYDRATE	0.88388199
GOBP_EPITHELIAL_TO_MESENCHYMAL_TRANSITION	0.88741336
GOBP_RESPONSE_TO_OXYGEN_CONTAINING_COMPOUND	0.89035849
GOBP_RECEPTOR_MEDIATED_ENDOCYTOSIS	0.89242343
GOBP_SEXUAL_REPRODUCTION	0.8932057
GOBP_RAS_PROTEIN_SIGNAL_TRANSDUCTION	0.89337879
GOBP_CELLULAR_RESPONSE_TO_RADIATION	0.89735394
GOBP_TELOMERE_MAINTENANCE_VIA_TELOMERE_LENGTHENING	0.89871557
GOBP_CELLULAR_SENESCENCE	0.89891991
GOBP_CELLULAR_RESPONSE_TO_SALT	0.90183326
GOBP_LYSOSOMAL_TRANSPORT	0.90187252
GOBP_REGULATION_OF_PROTEIN_LOCALIZATION_TO_NUCLEUS	0.90326544
GOBP_NERVOUS_SYSTEM_PROCESS	0.90656332
GOBP_REGULATION_OF_SISTER_CHROMATID_SEGREGATION	0.91131262
GOBP_CYTOSOLIC_TRANSPORT	0.91262499
GOBP_SEX_DIFFERENTIATION	0.9130189
GOBP_CELL_CYCLE_CHECKPOINT_SIGNALING	0.91339777
GOBP_REGULATION_OF_PROTEIN_CONTAINING_COMPLEX_DISASSEMBLY	0.91603009
GOBP_REGULATION_OF_MUSCLE_SYSTEM_PROCESS	0.92141522
GOBP_NEGATIVE_REGULATION_OF_MULTICELLULAR_ORGANISMAL_PROCESS	0.92193013
GOBP_CELLULAR_RESPONSE_TO_CHEMICAL_STRESS	0.92196438
GOBP_LIPID_LOCALIZATION	0.92834576
GOBP_MESENCHYMAL_CELL_DIFFERENTIATION	0.92888492
GOBP_SENSORY_ORGAN_MORPHOGENESIS	0.92987728
GOBP_REGULATION_OF_INTRACELLULAR_TRANSPORT	0.93139065
GOBP_ERK1_AND_ERK2_CASCADE	0.93720746
GOBP_HEMATOPOIETIC_PROGENITOR_CELL_DIFFERENTIATION	0.93803392
GOBP_LEUKOCYTE_APOPTOTIC_PROCESS	0.94156144
GOBP_POSITIVE_REGULATION_OF_MAPK_CASCADE	0.94217455
GOBP_ENDOMEMBRANE_SYSTEM_ORGANIZATION	0.94645179
GOBP_SECOND_MESSENGER_MEDIATED_SIGNALING	0.94850931
GOBP_NEGATIVE_REGULATION_OF_CELL_CYCLE_PROCESS	0.94926473
GOBP_MITOTIC_CELL_CYCLE_PHASE_TRANSITION	0.94959784
GOBP_CELL_MATRIX_ADHESION	0.95040236
GOBP_POSITIVE_REGULATION_OF_CYSTEINE_TYPE_ENDOPEPTIDASE_ACTIVITY	0.95104236
GOBP_RESPONSE_TO_MONOSACCHARIDE	0.95172853
GOBP_RNA_SPLICING_VIA_TRANSESTERIFICATION_REACTIONS	0.95279366
GOBP_NEGATIVE_REGULATION_OF_AUTOPHAGY	0.95327694
GOBP_SENSORY_PERCEPTION	0.95331408
GOBP_REGULATION_OF_ANATOMICAL_STRUCTURE_SIZE	0.95343162
GOBP_POSITIVE_REGULATION_OF_TELOMERE_MAINTENANCE	0.95827562
GOBP_REGULATED_EXOCYTOSIS	0.95913413
GOBP_POSITIVE_REGULATION_OF_CELL_SUBSTRATE_ADHESION	0.96058307
GOBP_ORGANIC_ANION_TRANSPORT	0.96189971

GOBP_CELLULAR_COMPONENT_DISASSEMBLY	0.9630832
GOBP_PROTEIN_LOCALIZATION_TO_CHROMOSOME	0.96427141
GOBP_DNA_MODIFICATION	0.96537385
GOBP_PROTEIN_COMPLEX_OLIGOMERIZATION	0.96755105
GOBP_MEIOTIC_CELL_CYCLE	0.96928651
GOBP_CELLULAR_RESPONSE_TO_REACTIVE_OXYGEN_SPECIES	0.96968011
GOBP_VESICLE_MEDIATED_TRANSPORT_TO_THE_PLASMA_MEMBRANE	0.97089208
GOBP_NOTCH_SIGNALING_PATHWAY	0.97220944
GOBP_REGULATION_OF_NEUROGENESIS	0.97257706
GOBP_PALLIUM_DEVELOPMENT	0.97307165
GOBP_POSITIVE_REGULATION_OF_CHROMOSOME_ORGANIZATION	0.97345636
GOBP_RESPONSE_TO_NUTRIENT	0.97468489
GOBP_ADAPTIVE_THERMOGENESIS	0.9751106
GOBP_NEGATIVE_REGULATION_OF_CATALYTIC_ACTIVITY	0.97547181
GOBP_ENDOPLASMIC_RETICULUM_ORGANIZATION	0.97585011
GOBP_CARBOHYDRATE_HOMEOSTASIS	0.9771678
GOBP_ORGANIC_HYDROXY_COMPOUND_TRANSPORT	0.9775902
GOBP_REGULATION_OF_CARBOHYDRATE_METABOLIC_PROCESS	0.97800323
GOBP_RESPONSE_TO_REACTIVE_OXYGEN_SPECIES	0.97808865
GOBP_CELLULAR_RESPONSE_TO_EXTERNAL_STIMULUS	0.97855371
GOBP_REGULATION_OF_CELL_DIVISION	0.97863311
GOBP_NEGATIVE_REGULATION_OF_NERVOUS_SYSTEM_DEVELOPMENT	0.97910306
GOBP_POST_GOLGI_VESICLE_MEDIATED_TRANSPORT	0.97913973
GOBP_POSITIVE_REGULATION_OF_DNA_METABOLIC_PROCESS	0.97918663
GOBP_NEGATIVE_REGULATION_OF_CELL_DIFFERENTIATION	0.97939679
GOBP_MULTICELLULAR_ORGANISM_GROWTH	0.9797636
GOBP_EMBRYONIC_MORPHOGENESIS	0.97990798
GOBP_LIPID_CATABOLIC_PROCESS	0.98222819
GOBP_HORMONE_MEDIATED_SIGNALING_PATHWAY	0.98349766
GOBP_REGULATION_OF_PEPTIDE_TRANSPORT	0.98351852
GOBP_NEGATIVE_REGULATION_OF_DNA_METABOLIC_PROCESS	0.98454407
GOBP_MRNA_PROCESSING	0.98534821
GOBP_EMBRYONIC_ORGAN_MORPHOGENESIS	0.98582706
GOBP_CONNECTIVE_TISSUE_DEVELOPMENT	0.98631718
GOBP_REGULATION_OF_PROTEIN_TARGETING	0.98642628
GOBP_EPITHELIAL_CELL_DEVELOPMENT	0.98698505
GOBP_REGULATION_OF_MRNA_METABOLIC_PROCESS	0.98714
GOBP_EPITHELIUM_DEVELOPMENT	0.98776121
GOBP_PROTEIN_CONTAINING_COMPLEX_DISASSEMBLY	0.98820012
GOBP_TISSUE_MORPHOGENESIS	0.98886505
GOBP_REGULATION_OF_DNA_BIOSYNTHETIC_PROCESS	0.98956393
GOBP_REGULATION_OF_INTRACELLULAR_PROTEIN_TRANSPORT	0.99035971
GOBP_CELLULAR_RESPONSE_TO_LIGHT_STIMULUS	0.99078888
GOBP_RNA_TEMPLATED_DNA_BIOSYNTHETIC_PROCESS	0.99154628
GOBP_RESPIRATORY_SYSTEM_DEVELOPMENT	0.9918237
GOBP_REGULATION_OF_GROWTH	0.99201775
GOBP_REGULATION_OF_HORMONE_SECRETION	0.99245261
GOBP_NEGATIVE_REGULATION_OF_DEFENSE_RESPONSE	0.99264557
GOBP_MRNA_EXPORT_FROM_NUCLEUS	0.99283888
GOBP_DEPHOSPHORYLATION	0.992874
GOBP_RHO_PROTEIN_SIGNAL_TRANSDUCTION	0.99293333
GOBP_MITOCHONDRIAL_TRANSLATION	0.9930317
GOBP_REGULATION_OF_DNA_TEMPLATED_TRANSCRIPTION_ELONGATION	0.99322413
GOBP_PHOSPHOLIPID_BIOSYNTHETIC_PROCESS	0.99400985
GOBP_REGULATION_OF_MRNA_PROCESSING	0.99417051
GOBP_NEGATIVE_REGULATION_OF_GROWTH	0.99455716
GOBP_NEGATIVE_REGULATION_OF_CELL_DEVELOPMENT	0.99468733
GOBP_EPITHELIAL_CELL_APOPTOTIC_PROCESS	0.99494106
GOBP_REGULATION_OF_SMALL_MOLECULE_METABOLIC_PROCESS	0.99506128
GOBP_NEGATIVE_REGULATION_OF_IMMUNE_RESPONSE	0.99536606
GOBP_PROTEIN_AUTOPHOSPHORYLATION	0.99539047
GOBP_COGNITION	0.99543275
GOBP_POSITIVE_REGULATION_OF_PEPTIDYL_TYROSINE_PHOSPHORYLATION	0.99563184
GOBP_GROWTH	0.99564334

GOBP_POSITIVE_REGULATION_OF_PROTEIN_LOCALIZATION_TO_NUCLEUS	0.99580915
GOBP_RECEPTOR_INTERNALIZATION	0.99599389
GOBP_ENDOSOMAL_TRANSPORT	0.99603241
GOBP_CELL_PROJECTION_ASSEMBLY	0.99615365
GOBP_CELLULAR_RESPONSE_TO_OXYGEN_CONTAINING_COMPOUND	0.99619511
GOBP_EPITHELIAL_CELL_DIFFERENTIATION	0.99624596
GOBP_POSITIVE_REGULATION_OF_PROTEIN_LOCALIZATION_TO_MEMBRANE	0.99631655
GOBP_REGULATION_OF_LIPID_METABOLIC_PROCESS	0.99649721
GOBP_NEGATIVE_REGULATION_OF_SECRETION	0.99654422
GOBP_NEGATIVE_REGULATION_OF_RESPONSE_TO_BIOTIC_STIMULUS	0.99656945
GOBP_GLYCEROLIPID_METABOLIC_PROCESS	0.99661402
GOBP_FOREBRAIN_DEVELOPMENT	0.99666506
GOBP_CALCIUM_MEDIATED_SIGNALING	0.99680494
GOBP_CELLULAR_RESPONSE_TO_NITROGEN_COMPOUND	0.99764738
GOBP_REGULATION_OF_DOUBLE_STRAND_BREAK_REPAIR	0.99768106
GOBP_POSITIVE_REGULATION_OF_INTRACELLULAR_PROTEIN_TRANSPORT	0.99784585
GOBP_REGULATION_OF_NERVOUS_SYSTEM_DEVELOPMENT	0.99791241
GOBP_DEVELOPMENTAL_MATURATION	0.99793677
GOBP_NEGATIVE_REGULATION_OF_INNATE_IMMUNE_RESPONSE	0.99847136
GOBP_GLYCEROLIPID_BIOSYNTHETIC_PROCESS	0.99848666
GOBP_REGULATION_OF_DEPHOSPHORYLATION	0.99857605
GOBP_NEURON_APOPTOTIC_PROCESS	0.99876458
GOBP_NEGATIVE_REGULATION_OF_RESPONSE_TO_EXTERNAL_STIMULUS	0.99889428
GOBP_CELL_GROWTH	0.99906029
GOBP_DNA_BIOSYNTHETIC_PROCESS	0.99909698
GOBP_LOCOMOTORY_BEHAVIOR	0.99914937
GOBP_MAMMARY_GLAND_DEVELOPMENT	0.99921006
GOBP_REGULATION_OF_PEPTIDYL_TYROSINE_PHOSPHORYLATION	0.9992421
GOBP_NEGATIVE_REGULATION_OF_CELL_GROWTH	0.99929533
GOBP_RESPONSE_TO_TOXIC_SUBSTANCE	0.9993051
GOBP_CELL_CYCLE_PHASE_TRANSITION	0.99934547
GOBP_POSITIVE_REGULATION_OF_MITOTIC_CELL_CYCLE	0.99937299
GOBP_MESENCHYME_DEVELOPMENT	0.99944493
GOBP_REGULATION_OF_TELOMERE_MAINTENANCE_VIA_TELOMERE_LENGTHENING	0.99962048
GOBP_TELENCEPHALON_DEVELOPMENT	0.99971752
GOBP_EMBRYONIC_ORGAN_DEVELOPMENT	0.99973324
GOBP_DENDRITE_DEVELOPMENT	0.99974254
GOBP_REGULATION_OF_CELL_GROWTH	0.9997711
GOBP_RNA_EXPORT_FROM_NUCLEUS	0.99983389
GOBP_TRANSCRIPTION_BY_RNA_POLYMERASE_I	0.99983626
GOBP_PROTEIN_LOCALIZATION_TO_NUCLEUS	0.99985351
GOBP_POSITIVE_REGULATION_OF_DNA_REPAIR	0.99985847
GOBP_EPITHELIAL_CELL_PROLIFERATION	0.99988396
GOBP_CARBOHYDRATE_CATABOLIC_PROCESS	0.99988696
GOBP_RESPONSE_TO_EXTRACELLULAR_STIMULUS	0.99988915
GOBP_SPLICEOSOMAL_COMPLEX_ASSEMBLY	0.99989634
GOBP_RESPONSE_TO_IONIZING_RADIATION	0.99991
GOBP_RESPONSE_TO_KETONE	0.99991758
GOBP_MITOCHONDRIAL_GENE_EXPRESSION	0.99992693
GOBP_POSITIVE_REGULATION_OF_DOUBLE_STRAND_BREAK_REPAIR	0.99992843
GOBP_CELLULAR_KETONE_METABOLIC_PROCESS	0.99993815
GOBP_POSITIVE_REGULATION_OF_ESTABLISHMENT_OF_PROTEIN_LOCALIZATION	0.99994358
GOBP_NEGATIVE_REGULATION_OF_ESTABLISHMENT_OF_PROTEIN_LOCALIZATION	0.999957
GOBP_ANTERIOR_POSTERIOR_PATTERN_SPECIFICATION	0.9999601
GOBP_PATTERN_SPECIFICATION_PROCESS	0.99996583
GOBP_ORGANIC_ACID_TRANSPORT	0.9999695
GOBP_PHOSPHOLIPID_METABOLIC_PROCESS	0.99997325
GOBP_POSITIVE_REGULATION_OF_WNT_SIGNALING_PATHWAY	0.99997422
GOBP_LIPID_MODIFICATION	0.99998322
GOBP_PEPTIDYL_TYROSINE_MODIFICATION	0.99998338
GOBP_MEIOTIC_CELL_CYCLE_PROCESS	0.99998639
GOBP_SKELETAL_SYSTEM_DEVELOPMENT	0.99998672
GOBP_MAINTENANCE_OF_CELL_NUMBER	0.99998792
GOBP_ENDOTHELIUM_DEVELOPMENT	0.99998964

GOBP_REGULATION_OF_EXOCYTOSIS	0.99999414
GOBP_TORC1_SIGNALING	0.99999507
GOBP_REGULATION_OF_CANONICAL_WNT_SIGNALING_PATHWAY	0.99999699
GOBP_RETROGRADE_TRANSPORT_ENDOSOME_TO_GOLGI	0.99999719
GOBP_NEURAL_PRECURSOR_CELL_PROLIFERATION	0.9999973
GOBP_POSITIVE_REGULATION_OF_NEURON_PROJECTION_DEVELOPMENT	0.99999743
GOBP_NEGATIVE_REGULATION_OF_HYDROLASE_ACTIVITY	0.99999815
GOBP_CELL_SUBSTRATE_JUNCTION_ORGANIZATION	0.99999836
GOBP_NUCLEUS_ORGANIZATION	0.99999887
GOBP_INSULIN_RECEPTOR_SIGNALING_PATHWAY	0.99999959
GOBP_CANONICAL_WNT_SIGNALING_PATHWAY	0.99999963
GOBP_NEGATIVE_REGULATION_OF_CELL_PROJECTION_ORGANIZATION	0.99999996
GOBP_ALTERNATIVE_MRNA_SPLICING_VIA_SPLICEOSOME	0.99999999
GOBP_HEMATOPOIETIC_OR_LYMPHOID_ORGAN_DEVELOPMENT	1
