# Supplementary material for: GSNCASCR: An R Package to Identify Differentially Co-Expressed Curated Gene Sets with Single-Cell RNA-Seq Data
Source: Int J Mol Sci. 2025 May 16;26(10):4771. doi: 10.3390/ijms26104771 (PMC12112291; doi:10.3390/ijms26104771)
Supplement: Supplementary file 1 [file ijms-26-04771-s001.zip › Table S3-GO enrichment analysis of CD8+ T cells.pdf]

Table S3. Gene Ontology enrichment analysis of CD8$^+$ T cells.

GO term	*P* value
GOBP_REGULATION_OF_TELOMERE_MAINTENANCE	3.68E-24
GOBP_TELOMERE_MAINTENANCE	1.35E-23
GOBP_POSITIVE_REGULATION_OF_TELOMERE_MAINTENANCE	1.64E-22
GOBP_DEFENSE_RESPONSE_TO_OTHER_ORGANISM	1.90E-22
GOBP_POSITIVE_REGULATION_OF_IMMUNE_SYSTEM_PROCESS	2.13E-22
GOBP_AMIDE_BIOSYNTHETIC_PROCESS	2.42E-22
GOBP_PEPTIDE_BIOSYNTHETIC_PROCESS	3.80E-22
GOBP_PROTEIN_LOCALIZATION_TO_CHROMOSOME	4.39E-22
GOBP_TELOMERE_ORGANIZATION	7.69E-22
GOBP_REGULATION_OF_PHOSPHORUS_METABOLIC_PROCESS	7.85E-22
GOBP_PEPTIDE_METABOLIC_PROCESS	1.33E-21
GOBP_PROTEIN_MATURATION	1.66E-21
GOBP_TELOMERE_MAINTENANCE_VIA_TELOMERE_LENGTHENING	2.57E-21
GOBP_REGULATION_OF_TRANSFERASE_ACTIVITY	3.68E-21
GOBP_REPRODUCTION	5.34E-21
GOBP_RNA_TEMPLATED_DNA_BIOSYNTHETIC_PROCESS	5.62E-21
GOBP_POSITIVE_REGULATION_OF_ORGANELLE_ORGANIZATION	6.52E-21
GOBP_PROTEIN_STABILIZATION	7.20E-21
GOBP_DNA_REPLICATION	1.08E-20
GOBP_PROTEIN_FOLDING	1.18E-20
GOBP_PROTEIN_LOCALIZATION_TO_NUCLEUS	1.32E-20
GOBP_RIBOSOME_BIOGENESIS	1.35E-20
GOBP_POSITIVE_REGULATION_OF_IMMUNE_RESPONSE	1.91E-20
GOBP_REGULATION_OF_IMMUNE_RESPONSE	2.43E-20
GOBP_RIBONUCLEOPROTEIN_COMPLEX_BIOGENESIS	2.71E-20
GOBP_INNATE_IMMUNE_RESPONSE	2.96E-20
GOBP_AMIDE_METABOLIC_PROCESS	3.41E-20
GOBP_REGULATION_OF_PHOSPHORYLATION	4.33E-20
GOBP_CELL_KILLING	5.21E-20
GOBP_IMMUNE_EFFECTOR_PROCESS	5.41E-20
GOBP_RESPONSE_TO_CYTOKINE	5.63E-20
GOBP_REGULATION_OF_KINASE_ACTIVITY	6.63E-20
GOBP_REGULATION_OF_PROTEIN_STABILITY	7.18E-20
GOBP_RESPONSE_TO_ORGANIC_CYCLIC_COMPOUND	7.43E-20
GOBP_CHROMOSOME_ORGANIZATION	1.17E-19
GOBP_POSITIVE_REGULATION_OF_CHROMOSOME_ORGANIZATION	1.24E-19
GOBP_ADAPTIVE_IMMUNE_RESPONSE	1.28E-19
GOBP_OSSIFICATION	1.63E-19
GOBP_HOMEOSTATIC_PROCESS	1.65E-19
GOBP_RESPONSE_TO_TOPOLOGICALLY_INCORRECT_PROTEIN	1.93E-19
GOBP_RESPONSE_TO_VIRUS	1.98E-19
GOBP_POSITIVE_REGULATION_OF_PROTEIN_LOCALIZATION_TO_NUCLEUS	2.48E-19
GOBP_MITOTIC_NUCLEAR_DIVISION	2.81E-19
GOBP_POSITIVE_REGULATION_OF_GENE_EXPRESSION	2.93E-19
GOBP_POSITIVE_REGULATION_OF_TRANSFERASE_ACTIVITY	3.16E-19
GOBP_REGULATION_OF_DNA_BIOSYNTHETIC_PROCESS	3.34E-19
GOBP_RESPONSE_TO_NITROGEN_COMPOUND	3.34E-19
GOBP_POSITIVE_REGULATION_OF_TRANSCRIPTION_BY_RNA_POLYMERASE_II	4.31E-19
GOBP_ORGANELLE_FISSION	5.08E-19
GOBP_RESPONSE_TO_XENOBIOTIC_STIMULUS	7.32E-19
GOBP_PROTEIN_LOCALIZATION_TO_ORGANELLE	9.20E-19
GOBP_ACTIVATION_OF_IMMUNE_RESPONSE	1.24E-18
GOBP_POSITIVE_REGULATION_OF_PROGRAMMED_CELL_DEATH	1.36E-18
GOBP_POSITIVE_REGULATION_OF_PROTEIN_LOCALIZATION	1.43E-18
GOBP_CELLULAR_COMPONENT_DISASSEMBLY	1.50E-18
GOBP_NUCLEOSOME_ORGANIZATION	1.61E-18
GOBP_POSITIVE_REGULATION_OF_RESPONSE_TO_EXTERNAL_STIMULUS	1.70E-18
GOBP_REGULATION_OF_CATALYTIC_ACTIVITY	2.14E-18
GOBP_RESPONSE_TO_ABIOTIC_STIMULUS	2.52E-18

GOBP_REGULATION_OF_DEFENSE_RESPONSE	2.75E-18
GOBP_CYTOSKELETON_ORGANIZATION	2.82E-18
GOBP_POSITIVE_REGULATION_OF_CELLULAR_COMPONENT_ORGANIZATION	3.23E-18
GOBP_DNA_BIOSYNTHETIC_PROCESS	3.46E-18
GOBP_DNA_METABOLIC_PROCESS	3.83E-18
GOBP_POSITIVE_REGULATION_OF_RESPONSE_TO_BIOTIC_STIMULUS	4.00E-18
GOBP_RESPONSE_TO_ENDOGENOUS_STIMULUS	4.00E-18
GOBP_REGULATION_OF_RESPONSE_TO_BIOTIC_STIMULUS	4.21E-18
GOBP_CELLULAR_RESPONSE_TO_OXYGEN_CONTAINING_COMPOUND	4.34E-18
GOBP_RRNA_METABOLIC_PROCESS	4.68E-18
GOBP_LEUKOCYTE_MEDIATED_IMMUNITY	4.73E-18
GOBP_GLAND_DEVELOPMENT	5.19E-18
GOBP_ESTABLISHMENT_OF_PROTEIN_LOCALIZATION_TO_ORGANELLE	6.32E-18
GOBP_POSITIVE_REGULATION_OF_CELL_ACTIVATION	6.56E-18
GOBP_REGULATION_OF_RESPONSE_TO_EXTERNAL_STIMULUS	7.25E-18
GOBP_CYTOKINE_PRODUCTION	8.49E-18
GOBP_TRANSMEMBRANE_TRANSPORT	8.57E-18
GOBP_RIBOSOMAL_SMALL_SUBUNIT_BIOGENESIS	8.67E-18
GOBP_MITOTIC_SISTER_CHROMATID_SEGREGATION	9.55E-18
GOBP_POSITIVE_REGULATION_OF_CELL_ADHESION	1.13E-17
GOBP_POSITIVE_REGULATION_OF_CELL_CYCLE	1.22E-17
GOBP_NEGATIVE_REGULATION_OF_PROGRAMMED_CELL_DEATH	1.23E-17
GOBP_HUMORAL_IMMUNE_RESPONSE	1.29E-17
GOBP_SEXUAL_REPRODUCTION	1.29E-17
GOBP_PROTEIN_CONTAINING_COMPLEX_DISASSEMBLY	1.32E-17
GOBP_OSTEOBLAST_DIFFERENTIATION	1.43E-17
GOBP_PROTEIN_CATABOLIC_PROCESS	1.47E-17
GOBP_REGULATION_OF_PROTEIN_LOCALIZATION_TO_NUCLEUS	1.52E-17
GOBP_REGULATION_OF_CELL_ACTIVATION	1.55E-17
GOBP_REGULATION_OF_CELL_CYCLE	1.67E-17
GOBP_REGULATION_OF_IMMUNE_EFFECTOR_PROCESS	1.99E-17
GOBP_ACTIVATION_OF_INNATE_IMMUNE_RESPONSE	2.12E-17
GOBP_REGULATION_OF_CELLULAR_LOCALIZATION	2.31E-17
GOBP_ORGANOPHOSPHATE_METABOLIC_PROCESS	2.43E-17
GOBP_POSITIVE_REGULATION_OF_MULTICELLULAR_ORGANISMAL_PROCESS	2.67E-17
GOBP_POSITIVE_REGULATION_OF_CELL_CYCLE_PROCESS	2.79E-17
GOBP_NUCLEAR_TRANSPORT	2.92E-17
GOBP_INTRACELLULAR_PROTEIN_TRANSPORT	3.16E-17
GOBP_SMALL_MOLECULE_METABOLIC_PROCESS	3.37E-17
GOBP_CENTRAL_NERVOUS_SYSTEM_DEVELOPMENT	3.43E-17
GOBP_POSITIVE_REGULATION_OF_CYTOKINE_PRODUCTION	3.51E-17
GOBP_REGULATION_OF_MULTICELLULAR_ORGANISMAL_DEVELOPMENT	3.52E-17
GOBP_CELL_ADHESION	3.77E-17
GOBP_POSITIVE_REGULATION_OF_DEFENSE_RESPONSE	3.89E-17
GOBP_NEGATIVE_REGULATION_OF_GENE_EXPRESSION	4.05E-17
GOBP_RESPONSE_TO_OXYGEN_CONTAINING_COMPOUND	4.77E-17
GOBP_NATURAL_KILLER_CELL_MEDIATED_IMMUNITY	4.87E-17
GOBP_CELL_CELL_ADHESION	5.24E-17
GOBP_CELL_ACTIVATION	5.55E-17
GOBP_CELLULAR_RESPONSE_TO_NITROGEN_COMPOUND	5.55E-17
GOBP_RESPONSE_TO_LIGHT_STIMULUS	5.60E-17
GOBP_SUPRAMOLECULAR_FIBER_ORGANIZATION	5.79E-17
GOBP_PROTEIN_PHOSPHORYLATION	5.86E-17
GOBP_LYMPHOCYTE_MEDIATED_IMMUNITY	5.87E-17
GOBP_NEGATIVE_REGULATION_OF_RNA_BIOSYNTHETIC_PROCESS	6.53E-17
GOBP_REGULATION_OF_INNATE_IMMUNE_RESPONSE	6.77E-17
GOBP_PROTEOLYSIS_INVOLVED_IN_PROTEIN_CATABOLIC_PROCESS	7.37E-17
GOBP_REGULATION_OF_ORGANELLE_ORGANIZATION	8.60E-17
GOBP_RESPONSE_TO_ENDOPLASMIC_RETICULUM_STRESS	8.89E-17
GOBP_CARBOHYDRATE_DERIVATIVE_METABOLIC_PROCESS	9.87E-17
GOBP_ALPHA_BETA_T_CELL_ACTIVATION	1.24E-16
GOBP_RECOMBINATIONAL_REPAIR	1.25E-16
GOBP_REGULATION_OF_CELL_ADHESION	1.25E-16
GOBP_RNA_LOCALIZATION	1.29E-16

GOBP_HEAD_DEVELOPMENT	1.33E-16
GOBP_MRNA_PROCESSING	1.37E-16
GOBP_NEGATIVE_REGULATION_OF_TRANSCRIPTION_BY_RNA_POLYMERASE_II	1.57E-16
GOBP_POSITIVE_REGULATION_OF_MOLECULAR_FUNCTION	1.58E-16
GOBP_PROCESS_UTILIZING_AUTOPHAGIC_MECHANISM	1.61E-16
GOBP_POSITIVE_REGULATION_OF_CELL_CELL_ADHESION	1.68E-16
GOBP_PRODUCTION_OF_MOLECULAR_MEDIATOR_OF_IMMUNE_RESPONSE	1.85E-16
GOBP_POSITIVE_REGULATION_OF_LEUKOCYTE_CELL_CELL_ADHESION	2.02E-16
GOBP_PROTEIN_RNA_COMPLEX_ORGANIZATION	2.11E-16
GOBP_REGULATION_OF_LYMPHOCYTE_ACTIVATION	2.42E-16
GOBP_POSITIVE_REGULATION_OF_PHOSPHORUS_METABOLIC_PROCESS	2.59E-16
GOBP_POSITIVE_REGULATION_OF_IMMUNE_EFFECTOR_PROCESS	2.69E-16
GOBP_MONOATOMIC_ION_TRANSMEMBRANE_TRANSPORT	3.33E-16
GOBP_CYTOPLASMIC_TRANSLATION	3.40E-16
GOBP_RESPONSE_TO_TYPE_I_INTERFERON	3.45E-16
GOBP_APOPTOTIC_SIGNALING_PATHWAY	3.48E-16
GOBP_REGULATION_OF_DNA_REPLICATION	3.65E-16
GOBP_MONOATOMIC_CATION_TRANSMEMBRANE_TRANSPORT	3.76E-16
GOBP_DEFENSE_RESPONSE_TO_SYMBIONT	3.92E-16
GOBP_INTRACELLULAR_RECEPTOR_SIGNALING_PATHWAY	4.27E-16
GOBP_POSITIVE_REGULATION_OF_PROTEIN_MODIFICATION_PROCESS	4.71E-16
GOBP_RESPONSE_TO_UV	4.82E-16
GOBP_RNA_SPLICING_VIA_TRANSESTERIFICATION_REACTIONS	5.37E-16
GOBP_POST_TRANSCRIPTIONAL_REGULATION_OF_GENE_EXPRESSION	5.84E-16
GOBP_RESPONSE_TO_HORMONE	5.90E-16
GOBP_NEGATIVE_REGULATION_OF_CATALYTIC_ACTIVITY	7.65E-16
GOBP_POSITIVE_REGULATION_OF_INTRACELLULAR_SIGNAL_TRANSDUCTION	7.72E-16
GOBP_LEUKOCYTE_CELL_CELL_ADHESION	8.21E-16
GOBP_PROTEIN_DNA_COMPLEX_ORGANIZATION	8.84E-16
GOBP_POSITIVE_REGULATION_OF_LYMPHOCYTE_ACTIVATION	9.33E-16
GOBP_IMMUNE_RESPONSE_REGULATING_SIGNALING_PATHWAY	9.39E-16
GOBP_REGULATION_OF_CATABOLIC_PROCESS	9.62E-16
GOBP_LEUKOCYTE_MEDIATED_CYTOTOXICITY	9.97E-16
GOBP_REGULATION_OF_PROTEIN_CONTAINING_COMPLEX_DISASSEMBLY	1.11E-15
GOBP_CELL_MOTILITY	1.13E-15
GOBP_ERBB_SIGNALING_PATHWAY	1.25E-15
GOBP_CELLULAR_RESPONSE_TO_ORGANIC_CYCLIC_COMPOUND	1.28E-15
GOBP_REGULATION_OF_CELLULAR_COMPONENT_BIOGENESIS	1.36E-15
GOBP_REGULATION_OF_PROTEIN_MODIFICATION_PROCESS	1.41E-15
GOBP_REGULATION_OF_T_CELL_ACTIVATION	1.63E-15
GOBP_PEPTIDYL_SERINE_MODIFICATION	1.75E-15
GOBP_REGULATION_OF_SIGNAL_TRANSDUCTION_BY_P53_CLASS_MEDIATOR	1.75E-15
GOBP_EMBRYO_DEVELOPMENT	1.91E-15
GOBP_POSITIVE_REGULATION_OF_PRODUCTION_OF_MOLECULAR_MEDIATOR_OF_IMMUNE_RESPONSE	1.97E-15
GOBP_PHAGOCYTOSIS	2.19E-15
GOBP_MACROAUTOPHAGY	2.35E-15
GOBP_PURINE_CONTAINING_COMPOUND_METABOLIC_PROCESS	2.72E-15
GOBP_INTRINSIC_APOPTOTIC_SIGNALING_PATHWAY	2.84E-15
GOBP_MICROTUBULE_CYTOSKELETON_ORGANIZATION	2.84E-15
GOBP_REGULATION_OF_TRANSPORT	2.86E-15
GOBP_RESPONSE_TO_ACID_CHEMICAL	2.91E-15
GOBP_ORGANELLE_ASSEMBLY	3.29E-15
GOBP_B_CELL_ACTIVATION	3.32E-15
GOBP_POSITIVE_REGULATION_OF_PROTEIN_METABOLIC_PROCESS	3.52E-15
GOBP_RNA_SPLICING	3.54E-15
GOBP_SIGNAL_TRANSDUCTION_BY_P53_CLASS_MEDIATOR	3.75E-15
GOBP_CELLULAR_RESPONSE_TO_LIGHT_STIMULUS	3.94E-15
GOBP_REGULATION_OF_CELL_DIFFERENTIATION	4.15E-15
GOBP_LYMPHOCYTE_ACTIVATION	4.45E-15
GOBP_POSITIVE_REGULATION_OF_PHOSPHORYLATION	4.51E-15
GOBP_NCRNA_METABOLIC_PROCESS	5.09E-15
GOBP_POSITIVE_REGULATION_OF_AMIDE_METABOLIC_PROCESS	5.39E-15
GOBP_REGULATION_OF_CELL_CELL_ADHESION	6.09E-15
GOBP_MUSCLE_CELL_PROLIFERATION	6.11E-15

GOBP_NUCLEOBASE_CONTAINING_SMALL_MOLECULE_METABOLIC_PROCESS	6.20E-15
GOBP_NEGATIVE_REGULATION_OF_CELLULAR_COMPONENT_ORGANIZATION	6.51E-15
GOBP_RESPONSE_TO_OXIDATIVE_STRESS	6.56E-15
GOBP_ALPHA_BETA_T_CELL_DIFFERENTIATION	6.65E-15
GOBP_MITOTIC_SPINDLE_ORGANIZATION	6.78E-15
GOBP_NEGATIVE_REGULATION_OF_SIGNALING	6.95E-15
GOBP_POSITIVE_REGULATION_OF_KINASE_ACTIVITY	7.27E-15
GOBP_REGULATION_OF_EPITHELIAL_CELL_PROLIFERATION	8.12E-15
GOBP_POSITIVE_REGULATION_OF_CELL_DIFFERENTIATION	8.17E-15
GOBP_MICROTUBULE_CYTOSKELETON_ORGANIZATION_INVOLVED_IN_MITOSIS	8.54E-15
GOBP_NCRNA_PROCESSING	8.90E-15
GOBP_POSITIVE_REGULATION_OF_CATALYTIC_ACTIVITY	9.19E-15
GOBP_CELLULAR_RESPONSE_TO_RADIATION	9.25E-15
GOBP_MONOATOMIC_CATION_TRANSPORT	9.41E-15
GOBP_ACTOMYOSIN_STRUCTURE_ORGANIZATION	9.48E-15
GOBP_REGULATION_OF_CELL_DEVELOPMENT	1.05E-14
GOBP_NEGATIVE_REGULATION_OF_PHOSPHORYLATION	1.08E-14
GOBP_CELLULAR_RESPONSE_TO_HORMONE_STIMULUS	1.10E-14
GOBP_LEUKOCYTE_MIGRATION	1.14E-14
GOBP_REGULATION_OF_ANATOMICAL_STRUCTURE_MORPHOGENESIS	1.14E-14
GOBP_ADAPTIVE_IMMUNE_RESPONSE_BASED_ON_SOMATIC_RECOMBINATION_OF_IMMUNE_RECEPTORS_ BUILT_FROM_IMMUNOGLOBULIN_SUPERFAMILY_DOMAINS	1.21E-14
GOBP_CHAPERONE_MEDIATED_PROTEIN_FOLDING	1.21E-14
GOBP_NEGATIVE_REGULATION_OF_MULTICELLULAR_ORGANISMAL_PROCESS	1.24E-14
GOBP_CELLULAR_RESPONSE_TO_LIPID	1.32E-14
GOBP_REGULATION_OF_AMIDE_METABOLIC_PROCESS	1.33E-14
GOBP_DEVELOPMENTAL_PROCESS_INVOLVED_IN_REPRODUCTION	1.38E-14
GOBP_DNA_REPAIR	1.41E-14
GOBP_DNA_RECOMBINATION	1.56E-14
GOBP_REGULATION_OF_LYMPHOCYTE_DIFFERENTIATION	1.65E-14
GOBP_RESPONSE_TO_GROWTH_FACTOR	1.67E-14
GOBP_MRNA_METABOLIC_PROCESS	1.73E-14
GOBP_REGULATION_OF_CELL_KILLING	1.79E-14
GOBP_REGULATION_OF_POST_TRANSLATIONAL_PROTEIN_MODIFICATION	1.83E-14
GOBP_RESPONSE_TO_STEROID_HORMONE	1.85E-14
GOBP_RESPONSE_TO_TEMPERATURE_STIMULUS	1.90E-14
GOBP_HEPATICOBILIARY_SYSTEM_DEVELOPMENT	1.93E-14
GOBP_PROTEASOMAL_PROTEIN_CATABOLIC_PROCESS	2.15E-14
GOBP_MONOATOMIC_ION_TRANSPORT	2.21E-14
GOBP_DNA_TEMPLATED_DNA_REPLICATION	2.21E-14
GOBP_CHROMOSOME_LOCALIZATION	2.24E-14
GOBP_HISTONE_MODIFICATION	2.50E-14
GOBP_CELLULAR_RESPONSE_TO_UV	2.63E-14
GOBP_EPITHELIAL_CELL_DIFFERENTIATION	2.70E-14
GOBP_GENERATION_OF_NEURONS	2.72E-14
GOBP_NEGATIVE_REGULATION_OF_KINASE_ACTIVITY	2.75E-14
GOBP_REGULATION_OF_INFLAMMATORY_RESPONSE	2.76E-14
GOBP_NEUROGENESIS	2.88E-14
GOBP_EPITHELIUM_DEVELOPMENT	3.23E-14
GOBP_CHROMOSOME_SEGREGATION	3.37E-14
GOBP_NEGATIVE_REGULATION_OF_MOLECULAR_FUNCTION	3.43E-14
GOBP_NEGATIVE_REGULATION_OF_PHOSPHORUS_METABOLIC_PROCESS	3.45E-14
GOBP_POSITIVE_REGULATION_OF_TRANSLATION	3.67E-14
GOBP_REGULATION_OF_DEFENSE_RESPONSE_TO_VIRUS	3.76E-14
GOBP_REGULATION_OF_CELL_MATRIX_ADHESION	3.86E-14
GOBP_RESPONSE_TO_LIPID	4.04E-14
GOBP_REGULATION_OF_INTRINSIC_APOPTOTIC_SIGNALING_PATHWAY	5.27E-14
GOBP_CHROMATIN_REMODELING	5.49E-14
GOBP_POSITIVE_REGULATION_OF_DNA_METABOLIC_PROCESS	5.51E-14
GOBP_REGULATION_OF_CHROMOSOME_ORGANIZATION	6.04E-14
GOBP_REGULATION_OF_ESTABLISHMENT_OF_PROTEIN_LOCALIZATION	6.72E-14
GOBP_ACTIN_FILAMENT_ORGANIZATION	7.08E-14
GOBP_T_CELL_RECEPTOR_SIGNALING_PATHWAY	7.22E-14
GOBP_GENERATION_OF_PRECURSOR_METABOLITES_AND_ENERGY	7.32E-14

GOBP_VIRAL_PROCESS	7.62E-14
GOBP_NEGATIVE_REGULATION_OF_CHROMOSOME_ORGANIZATION	7.67E-14
GOBP_MEMBRANE_ORGANIZATION	7.71E-14
GOBP_IMMUNOGLOBULIN_PRODUCTION	7.73E-14
GOBP_REGULATION_OF_APOPTOTIC_SIGNALING_PATHWAY	7.84E-14
GOBP_NUCLEAR_CHROMOSOME_SEGREGATION	8.53E-14
GOBP_CARBOHYDRATE_DERIVATIVE_BIOSYNTHETIC_PROCESS	8.73E-14
GOBP_POSITIVE_REGULATION_OF_LEUKOCYTE_MEDIATED_IMMUNITY	8.79E-14
GOBP_ACTIN_FILAMENT_BASED_PROCESS	9.09E-14
GOBP_RESPONSE_TO_RADIATION	9.45E-14
GOBP_T_CELL_MEDIATED_IMMUNITY	9.48E-14
GOBP_CELL_MATRIX_ADHESION	9.56E-14
GOBP_POSITIVE_REGULATION_OF_ESTABLISHMENT_OF_PROTEIN_LOCALIZATION	1.04E-13
GOBP_REGULATION_OF_LEUKOCYTE_DIFFERENTIATION	1.05E-13
GOBP_DNA_DAMAGE_RESPONSE	1.06E-13
GOBP_POSITIVE_REGULATION_OF_CELL_POPULATION_PROLIFERATION	1.11E-13
GOBP_ORGANOPHOSPHATE_BIOSYNTHETIC_PROCESS	1.13E-13
GOBP_REGULATION_OF_ADAPTIVE_IMMUNE_RESPONSE	1.26E-13
GOBP_PEPTIDYL_AMINO_ACID_MODIFICATION	1.28E-13
GOBP_REGULATION_OF_PRODUCTION_OF_MOLECULAR_MEDIATOR_OF_IMMUNE_RESPONSE	1.42E-13
GOBP_SISTER_CHROMATID_SEGREGATION	1.43E-13
GOBP_POSITIVE_REGULATION_OF_CELL_CYCLE_PHASE_TRANSITION	1.43E-13
GOBP_CHEMICAL_HOMEOSTASIS	1.60E-13
GOBP_POSITIVE_REGULATION_OF_WNT_SIGNALING_PATHWAY	1.61E-13
GOBP_BIOLOGICAL_PROCESS_INVOLVED_IN_SYMBIOTIC_INTERACTION	1.69E-13
GOBP_REGULATION_OF_PROTEIN_SERINE_THREONINE_KINASE_ACTIVITY	1.73E-13
GOBP_DNA_CONFORMATION_CHANGE	1.76E-13
GOBP_EPITHELIAL_CELL_PROLIFERATION	1.76E-13
GOBP_NEGATIVE_REGULATION_OF_PROTEIN_METABOLIC_PROCESS	1.84E-13
GOBP_REGULATION_OF_CELL_MORPHOGENESIS	1.84E-13
GOBP_CELLULAR_RESPONSE_TO_ABIOTIC_STIMULUS	1.92E-13
GOBP_INTERFERON_MEDIATED_SIGNALING_PATHWAY	1.96E-13
GOBP_RESPONSE_TO_KETONE	2.02E-13
GOBP_INFLAMMATORY_RESPONSE	2.15E-13
GOBP_LIPID_METABOLIC_PROCESS	2.35E-13
GOBP_CARBOHYDRATE_METABOLIC_PROCESS	2.44E-13
GOBP_REGULATION_OF_CELL_SUBSTRATE_ADHESION	2.46E-13
GOBP_REGULATION_OF_CELLULAR_CATABOLIC_PROCESS	2.65E-13
GOBP_POSITIVE_REGULATION_OF_LYMPHOCYTE_MEDIATED_IMMUNITY	2.73E-13
GOBP_MITOTIC_CELL_CYCLE	2.76E-13
GOBP_IMPORT_INTO_NUCLEUS	2.99E-13
GOBP_HEMOPOIESIS	3.02E-13
GOBP_POSITIVE_REGULATION_OF_PROTEIN_KINASE_ACTIVITY	3.06E-13
GOBP_CELL_ACTIVATION_INVOLVED_IN_IMMUNE_RESPONSE	3.16E-13
GOBP_REGULATION_OF_LYMPHOCYTE_MEDIATED_IMMUNITY	3.45E-13
GOBP_REGULATION_OF_AUTOPHAGY	4.29E-13
GOBP_ORGANIC_CYCLIC_COMPOUND_CATABOLIC_PROCESS	4.42E-13
GOBP_LYMPHOCYTE_ACTIVATION_INVOLVED_IN_IMMUNE_RESPONSE	4.66E-13
GOBP_CELLULAR_SENESCENCE	4.69E-13
GOBP_MITOCHONDRION_ORGANIZATION	4.76E-13
GOBP_FOREBRAIN_DEVELOPMENT	5.03E-13
GOBP_POSITIVE_REGULATION_OF_CYTOSKELETON_ORGANIZATION	5.18E-13
GOBP_CELL_MORPHOGENESIS	5.25E-13
GOBP_NEGATIVE_REGULATION_OF_CELL_ADHESION	5.39E-13
GOBP_VACUOLAR_TRANSPORT	5.81E-13
GOBP_POSITIVE_REGULATION_OF_ADAPTIVE_IMMUNE_RESPONSE	6.16E-13
GOBP_MITOTIC_CELL_CYCLE_PROCESS	6.28E-13
GOBP_MICROTUBULE_BASED_PROCESS	6.34E-13
GOBP_CYTOSOLIC_PATTERN_RECOGNITION_RECEPTOR_SIGNALING_PATHWAY	6.71E-13
GOBP_REGULATION_OF_PROTEIN_CATABOLIC_PROCESS	6.90E-13
GOBP_PROTEIN_DNA_COMPLEX_ASSEMBLY	7.01E-13
GOBP_T_CELL_DIFFERENTIATION_IN_THYMUS	7.62E-13
GOBP_NEGATIVE_REGULATION_OF_TRANSFERASE_ACTIVITY	7.77E-13
GOBP_REGULATION_OF_LEUKOCYTE_MEDIATED_IMMUNITY	7.80E-13

GOBP_RIBOSE_PHOSPHATE_METABOLIC_PROCESS	7.92E-13
GOBP_REGULATION_OF_MEMBRANE_POTENTIAL	8.47E-13
GOBP_MEIOTIC_CELL_CYCLE_PROCESS	8.69E-13
GOBP_MITOCHONDRIAL_GENE_EXPRESSION	8.94E-13
GOBP_NUCLEOSIDE_TRIPHOSPHATE_METABOLIC_PROCESS	9.12E-13
GOBP_CARBOHYDRATE_DERIVATIVE_CATABOLIC_PROCESS	9.22E-13
GOBP_REGULATION_OF_NEUROGENESIS	9.55E-13
GOBP_POSITIVE_REGULATION_OF_MITOTIC_CELL_CYCLE	9.57E-13
GOBP_CELL_SUBSTRATE_ADHESION	1.06E-12
GOBP_PROTON_TRANSMEMBRANE_TRANSPORT	1.19E-12
GOBP_ANATOMICAL_STRUCTURE_FORMATION_INVOLVED_IN_MORPHOGENESIS	1.29E-12
GOBP_REGULATION_OF_SMALL_GTPASE_MEDIATED_SIGNAL_TRANSDUCTION	1.34E-12
GOBP_CELLULAR_HOMEOSTASIS	1.46E-12
GOBP_POSITIVE_REGULATION_OF_DEVELOPMENTAL_PROCESS	1.58E-12
GOBP_CELLULAR_RESPONSE_TO_PEPTIDE	1.61E-12
GOBP_HETEROCYCLE_CATABOLIC_PROCESS	1.64E-12
GOBP_RESPONSE_TO_OXYGEN_LEVELS	1.67E-12
GOBP_POSITIVE_REGULATION_OF_CELL_DEVELOPMENT	1.73E-12
GOBP_BIOLOGICAL_PROCESS_INVOLVED_IN_INTERACTION_WITH_HOST	1.89E-12
GOBP_NEURON_DEVELOPMENT	1.91E-12
GOBP_IMPORT_INTO_CELL	1.92E-12
GOBP_CELLULAR_RESPONSE_TO_STEROID_HORMONE_STIMULUS	2.10E-12
GOBP_REGULATION_OF_PROTEIN_CONTAINING_COMPLEX_ASSEMBLY	2.19E-12
GOBP_RESPONSE_TO_TRANSFORMING_GROWTH_FACTOR_BETA	2.37E-12
GOBP_ENDOSOMAL_TRANSPORT	2.50E-12
GOBP_CENTRAL_NERVOUS_SYSTEM_NEURON_DIFFERENTIATION	2.55E-12
GOBP_POSITIVE_REGULATION_OF_ENDOCYTOSIS	2.85E-12
GOBP_REGULATION_OF_PROTEIN_UBIQUITINATION	2.96E-12
GOBP_CELL_DIVISION	2.96E-12
GOBP_CELLULAR_RESPONSE_TO_CHEMICAL_STRESS	3.21E-12
GOBP_METAPHASE_CHROMOSOME_ALIGNMENT	3.26E-12
GOBP_REGULATION_OF_CELL_CYCLE_PROCESS	3.49E-12
GOBP_REGULATION_OF_ALPHA_BETA_T_CELL_ACTIVATION	3.51E-12
GOBP_LEUKOCYTE_PROLIFERATION	4.15E-12
GOBP_SPINDLE_ORGANIZATION	4.21E-12
GOBP_RESPONSE_TO_HEAT	4.46E-12
GOBP_BIOLOGICAL_PROCESS_INVOLVED_IN_INTERACTION_WITH_SYMBIONT	5.01E-12
GOBP_POSITIVE_REGULATION_OF_LYMPHOCYTE_DIFFERENTIATION	5.45E-12
GOBP_G_PROTEIN_COUPLED_RECEPTOR_SIGNALING_PATHWAY	6.46E-12
GOBP_CELL_PROJECTION_ORGANIZATION	6.59E-12
GOBP_POSITIVE_REGULATION_OF_CANONICAL_NF_KAPPAB_SIGNAL_TRANSDUCTION	6.98E-12
GOBP_NEGATIVE_REGULATION_OF_MRNA_METABOLIC_PROCESS	8.21E-12
GOBP_VIRAL_LIFE_CYCLE	8.52E-12
GOBP_LOCOMOTION	8.80E-12
GOBP_CD4_POSITIVE_ALPHA_BETA_T_CELL_ACTIVATION	9.04E-12
GOBP_CIRCULATORY_SYSTEM_DEVELOPMENT	9.06E-12
GOBP_POSITIVE_REGULATION_OF_CELLULAR_COMPONENT_BIOGENESIS	1.03E-11
GOBP_ANIMAL_ORGAN_MORPHOGENESIS	1.07E-11
GOBP_NEGATIVE_REGULATION_OF_CELL_CELL_ADHESION	1.12E-11
GOBP_PROTEIN_ACETYLATION	1.23E-11
GOBP_CELL_CHEMOTAXIS	1.23E-11
GOBP_REGULATION_OF_CELLULAR_RESPONSE_TO_STRESS	1.26E-11
GOBP_IMMUNE_SYSTEM_DEVELOPMENT	1.34E-11
GOBP_TRANSCRIPTION_BY_RNA_POLYMERASE_I	1.37E-11
GOBP_ORGANIC_ACID_METABOLIC_PROCESS	1.38E-11
GOBP_PROTEIN_POLYUBIQUITINATION	1.41E-11
GOBP_NEGATIVE_REGULATION_OF_CELL_POPULATION_PROLIFERATION	1.48E-11
GOBP_NERVOUS_SYSTEM_PROCESS	1.68E-11
GOBP_LOCALIZATION_WITHIN_MEMBRANE	1.69E-11
GOBP_RESPONSE_TO_PEPTIDE	1.75E-11
GOBP_REPRODUCTIVE_SYSTEM_DEVELOPMENT	1.90E-11
GOBP_NUCLEAR_EXPORT	1.94E-11
GOBP_POST_TRANSLATIONAL_PROTEIN_MODIFICATION	2.28E-11
GOBP_POSITIVE_REGULATION_OF_PROTEIN_MODIFICATION_BY_SMALL_PROTEIN_CONJUGATION_OR_REMOVAL	2.42E-11

GOBP_POSITIVE_REGULATION_OF_CATABOLIC_PROCESS	2.50E-11
GOBP_SENSORY_PERCEPTION	2.56E-11
GOBP_SELECTIVE_AUTOPHAGY	2.70E-11
GOBP_IMMUNE_RESPONSE_REGULATING_CELL_SURFACE_RECEPTOR_SIGNALING_PATHWAY	2.91E-11
GOBP_REGULATION_OF_DNA_METABOLIC_PROCESS	3.18E-11
GOBP_ENERGY_DERIVATION_BY_OXIDATION_OF_ORGANIC_COMPOUNDS	3.31E-11
GOBP_NEGATIVE_REGULATION_OF_GENE_EXPRESSION_EPIGENETIC	3.33E-11
GOBP_TUBE_DEVELOPMENT	3.60E-11
GOBP_EPIGENETIC_REGULATION_OF_GENE_EXPRESSION	3.66E-11
GOBP_CELLULAR_RESPONSE_TO_PEPTIDE_HORMONE_STIMULUS	3.74E-11
GOBP_REGULATION_OF_VIRAL_GENOME_REPLICATION	3.89E-11
GOBP_NEGATIVE_REGULATION_OF_POST_TRANSLATIONAL_PROTEIN_MODIFICATION	3.92E-11
GOBP_SMOOTH_MUSCLE_CELL_PROLIFERATION	4.18E-11
GOBP_NEGATIVE_REGULATION_OF_IMMUNE_SYSTEM_PROCESS	4.35E-11
GOBP_VIRAL_GENOME_REPLICATION	4.56E-11
GOBP_RIBOSOMAL_LARGE_SUBUNIT_BIOGENESIS	4.99E-11
GOBP_CELLULAR_RESPONSE_TO_SALT	5.60E-11
GOBP_REGULATION_OF_BODY_FLUID_LEVELS	5.66E-11
GOBP_CELLULAR_RESPONSE_TO_INSULIN_STIMULUS	5.91E-11
GOBP_NUCLEOBASE_CONTAINING_COMPOUND_TRANSPORT	6.05E-11
GOBP_PROTEIN_TARGETING	6.51E-11
GOBP_MULTICELLULAR_ORGANISMAL_LEVEL_HOMEOSTASIS	6.63E-11
GOBP_REGULATION_OF_SMALL_MOLECULE_METABOLIC_PROCESS	6.84E-11
GOBP_ANTIGEN_PROCESSING_AND_PRESENTATION	6.86E-11
GOBP_ORGANELLE_LOCALIZATION	6.95E-11
GOBP_REGULATION_OF_MRNA_PROCESSING	7.84E-11
GOBP_REGULATION_OF_MRNA_SPLICING_VIA_SPLICEOSOME	8.22E-11
GOBP_NEGATIVE_REGULATION_OF_CELL_DEVELOPMENT	8.57E-11
GOBP_NEURON_APOPTOTIC_PROCESS	9.74E-11
GOBP_T_CELL_ACTIVATION_INVOLVED_IN_IMMUNE_RESPONSE	9.92E-11
GOBP_NEGATIVE_REGULATION_OF_APOPTOTIC_SIGNALING_PATHWAY	9.98E-11
GOBP_NEGATIVE_REGULATION_OF_PROTEOLYSIS	1.02E-10
GOBP_REGULATION_OF_CELL_JUNCTION_ASSEMBLY	1.05E-10
GOBP_CELLULAR_RESPONSE_TO_EXTRACELLULAR_STIMULUS	1.07E-10
GOBP_NEGATIVE_REGULATION_OF_CATABOLIC_PROCESS	1.14E-10
GOBP_ANTIGEN_RECEPTOR_MEDIATED_SIGNALING_PATHWAY	1.16E-10
GOBP_ORGANELLE_DISASSEMBLY	1.20E-10
GOBP_CYTOKINE_PRODUCTION_INVOLVED_IN_IMMUNE_RESPONSE	1.25E-10
GOBP_CELLULAR_RESPONSE_TO_OXYGEN_LEVELS	1.26E-10
GOBP_MITOCHONDRIAL_TRANSLATION	1.29E-10
GOBP_PROTEIN_LOCALIZATION_TO_MITOCHONDRION	1.29E-10
GOBP_T_CELL_ACTIVATION	1.30E-10
GOBP_MAPK_CASCADE	1.32E-10
GOBP_RNA_CATABOLIC_PROCESS	1.35E-10
GOBP_SKELETAL_SYSTEM_DEVELOPMENT	1.45E-10
GOBP_RESPONSE_TO_PEPTIDE_HORMONE	1.46E-10
GOBP_REGULATION_OF_GROWTH	1.53E-10
GOBP_REGULATION_OF_SYNAPSE_STRUCTURE_OR_ACTIVITY	1.53E-10
GOBP_REGULATION_OF_HYDROLASE_ACTIVITY	1.65E-10
GOBP_NEGATIVE_REGULATION_OF_IMMUNE_EFFECTOR_PROCESS	1.66E-10
GOBP_MONOCARBOXYLIC_ACID_METABOLIC_PROCESS	1.72E-10
GOBP_POSITIVE_REGULATION_OF_HEMOPOIESIS	1.77E-10
GOBP_NON_MEMBRANE_BOUNDED_ORGANELLE_ASSEMBLY	1.89E-10
GOBP_CONNECTIVE_TISSUE_DEVELOPMENT	2.00E-10
GOBP_LEUKOCYTE_CHEMOTAXIS	2.02E-10
GOBP_CELLULAR_RESPONSE_TO_EXTERNAL_STIMULUS	2.07E-10
GOBP_CELLULAR_RESPONSE_TO_DECREASED_OXYGEN_LEVELS	2.11E-10
GOBP_REGULATION_OF_DEPHOSPHORYLATION	2.14E-10
GOBP_INORGANIC_ION_HOMEOSTASIS	2.21E-10
GOBP_POSITIVE_REGULATION_OF_POST_TRANSLATIONAL_PROTEIN_MODIFICATION	2.30E-10
GOBP_NEGATIVE_REGULATION_OF_PROTEIN_UBIQUITINATION	2.34E-10
GOBP_TRNA_METABOLIC_PROCESS	2.37E-10
GOBP_REGULATION_OF_T_CELL_MEDIATED_IMMUNITY	2.53E-10
GOBP_NEGATIVE_REGULATION_OF_DEVELOPMENTAL_PROCESS	2.55E-10

GOBP_AEROBIC_RESPIRATION	2.67E-10
GOBP_POSITIVE_REGULATION_OF_PROTEIN_UBIQUITINATION	2.67E-10
GOBP_REGULATION_OF_HORMONE_LEVELS	2.70E-10
GOBP_REGULATION_OF_CHROMOSOME_SEGREGATION	2.79E-10
GOBP_RESPONSE_TO_LEUKEMIA_INHIBITORY_FACTOR	2.85E-10
GOBP_B_CELL_DIFFERENTIATION	3.28E-10
GOBP_PROTEIN_ACYLATION	3.29E-10
GOBP_NUCLEOTIDE_EXCISION_REPAIR	3.32E-10
GOBP_RESPONSE_TO_WOUNDING	3.50E-10
GOBP_WOUND_HEALING	3.55E-10
GOBP_NCRNA_TRANSCRIPTION	3.56E-10
GOBP_CELLULAR_RESPIRATION	3.65E-10
GOBP_SECRETION	3.76E-10
GOBP_POSITIVE_REGULATION_OF_PHOSPHATIDYLINOSITOL_3_KINASE_PROTEIN_KINASE_B_SIGNAL_TRANSDUCTION	3.81E-10
GOBP_REGULATION_OF_MACROAUTOPHAGY	4.07E-10
GOBP_NEGATIVE_REGULATION_OF_CELL_ACTIVATION	4.28E-10
GOBP_NEGATIVE_REGULATION_OF_DEFENSE_RESPONSE	4.36E-10
GOBP_REGULATION_OF_CYTOSKELETON_ORGANIZATION	4.53E-10
GOBP_ESTABLISHMENT_OF_ORGANELLE_LOCALIZATION	4.58E-10
GOBP_NEGATIVE_REGULATION_OF_RESPONSE_TO_EXTERNAL_STIMULUS	4.61E-10
GOBP_CELL_CELL_SIGNALING	4.80E-10
GOBP_EXPORT_FROM_CELL	5.13E-10
GOBP_REGULATION_OF_HEMOPOIESIS	5.43E-10
GOBP_CELL_SUBSTRATE_JUNCTION_ORGANIZATION	5.82E-10
GOBP_CELLULAR_LIPID_METABOLIC_PROCESS	6.14E-10
GOBP_CELL_JUNCTION_ORGANIZATION	6.63E-10
GOBP_NUCLEOSIDE_PHOSPHATE_BIOSYNTHETIC_PROCESS	6.63E-10
GOBP_HOMEOSTASIS_OF_NUMBER_OF_CELLS	7.11E-10
GOBP_GROWTH	7.31E-10
GOBP_MODULATION_BY_HOST_OF_SYMBIONT_PROCESS	7.52E-10
GOBP_REGULATION_OF_ACTIN_FILAMENT_ORGANIZATION	7.86E-10
GOBP_PLATELET_ACTIVATION	8.00E-10
GOBP_POSITIVE_REGULATION_OF_LOCOMOTION	8.02E-10
GOBP_POSITIVE_REGULATION_OF_INTRACELLULAR_TRANSPORT	8.44E-10
GOBP_POSITIVE_REGULATION_OF_TRANSPORT	8.53E-10
GOBP_RENAL_SYSTEM_DEVELOPMENT	8.99E-10
GOBP_POSITIVE_REGULATION_OF_PROTEIN_CATABOLIC_PROCESS	9.59E-10
GOBP_RHYTHMIC_PROCESS	9.63E-10
GOBP_PROTEIN_PROCESSING	9.72E-10
GOBP_MACROMOLECULE_METHYLATION	1.01E-09
GOBP_POSITIVE_REGULATION_OF_CELLULAR_CATABOLIC_PROCESS	1.04E-09
GOBP_DOUBLE_STRAND_BREAK_REPAIR	1.07E-09
GOBP_REGULATION_OF_LIPID_METABOLIC_PROCESS	1.10E-09
GOBP_MONONUCLEAR_CELL_DIFFERENTIATION	1.15E-09
GOBP_ENTRY_INTO_HOST	1.17E-09
GOBP_NEURON_PROJECTION_EXTENSION	1.18E-09
GOBP_TRANSFORMING_GROWTH_FACTOR_BETA_RECEPTOR_SIGNALING_PATHWAY	1.22E-09
GOBP_TUBE_MORPHOGENESIS	1.24E-09
GOBP_ENDOCYTOSIS	1.24E-09
GOBP_NEGATIVE_REGULATION_OF_PROTEIN_MODIFICATION_PROCESS	1.29E-09
GOBP_REGULATION_OF_MITOTIC_CELL_CYCLE	1.35E-09
GOBP_ORGANELLE_FUSION	1.36E-09
GOBP_CELLULAR_RESPONSE_TO_OXIDATIVE_STRESS	1.37E-09
GOBP_NEGATIVE_REGULATION_OF_VIRAL_PROCESS	1.39E-09
GOBP_CANONICAL_NF_KAPPAB_SIGNAL_TRANSDUCTION	1.39E-09
GOBP_REGULATION_OF_PEPTIDYL_TYROSINE_PHOSPHORYLATION	1.46E-09
GOBP_ALTERNATIVE_MRNA_SPLICING_VIA_SPLICEOSOME	1.54E-09
GOBP_ESTABLISHMENT_OR_MAINTENANCE_OF_CELL_POLARITY	1.55E-09
GOBP_MYELOID_LEUKOCYTE_ACTIVATION	1.55E-09
GOBP_LEUKOCYTE_DIFFERENTIATION	1.83E-09
GOBP_CELL_CYCLE_PHASE_TRANSITION	1.85E-09
GOBP_RESPONSE_TO_STARVATION	1.86E-09
GOBP_ATP_METABOLIC_PROCESS	1.92E-09
GOBP_POSITIVE_REGULATION_OF_T_CELL_PROLIFERATION	1.94E-09

GOBP_REGULATION_OF_LOCOMOTION	2.13E-09
GOBP_MULTICELLULAR_ORGANISM_REPRODUCTION	2.27E-09
GOBP_CELLULAR_RESPONSE_TO_BIOTIC_STIMULUS	2.29E-09
GOBP_RESPONSE_TO_HYDROGEN_PEROXIDE	2.39E-09
GOBP_TYPE_II_INTERFERON_PRODUCTION	2.61E-09
GOBP_NEGATIVE_REGULATION_OF_CELL_CYCLE	2.66E-09
GOBP_ESTABLISHMENT_OF_RNA_LOCALIZATION	2.76E-09
GOBP_NEGATIVE_REGULATION_OF_NEURON_APOPTOTIC_PROCESS	2.76E-09
GOBP_NEGATIVE_REGULATION_OF_INTRACELLULAR_SIGNAL_TRANSDUCTION	2.97E-09
GOBP_CIRCADIAN_RHYTHM	3.04E-09
GOBP_NEGATIVE_REGULATION_OF_LEUKOCYTE_CELL_CELL_ADHESION	3.11E-09
GOBP_REGULATION_OF_RAS_PROTEIN_SIGNAL_TRANSDUCTION	3.11E-09
GOBP_LYSOSOMAL_TRANSPORT	3.19E-09
GOBP_NEURAL_PRECURSOR_CELL_PROLIFERATION	3.21E-09
GOBP_NEGATIVE_REGULATION_OF_CYTOKINE_PRODUCTION	3.26E-09
GOBP_SIGNAL_TRANSDUCTION_IN_RESPONSE_TO_DNA_DAMAGE	3.30E-09
GOBP_MEIOTIC_CELL_CYCLE	3.33E-09
GOBP_MYELOID_CELL_DIFFERENTIATION	3.53E-09
GOBP_ORGANIC_HYDROXY_COMPOUND_METABOLIC_PROCESS	3.57E-09
GOBP_TRANSMEMBRANE_RECEPTOR_PROTEIN_TYROSINE_KINASE_SIGNALING_PATHWAY	3.92E-09
GOBP_RESPONSE_TO_MECHANICAL_STIMULUS	4.23E-09
GOBP_STEM_CELL_DIFFERENTIATION	4.61E-09
GOBP_REGULATION_OF_ACTIN_FILAMENT_LENGTH	4.85E-09
GOBP_RESPONSE_TO_EXTRACELLULAR_STIMULUS	5.02E-09
GOBP_CD4_POSITIVE_ALPHA_BETA_T_CELL_DIFFERENTIATION	5.08E-09
GOBP_REGULATION_OF_NEURON_APOPTOTIC_PROCESS	5.16E-09
GOBP_CELL_GROWTH	5.35E-09
GOBP_LIPOPROTEIN_METABOLIC_PROCESS	5.40E-09
GOBP_REGULATION_OF_VASCULATURE_DEVELOPMENT	5.65E-09
GOBP_REGULATION_OF_CELL_SIZE	5.68E-09
GOBP_MAMMARY_GLAND_DEVELOPMENT	5.86E-09
GOBP_OXIDATIVE_PHOSPHORYLATION	5.93E-09
GOBP_POSITIVE_REGULATION_OF_DNA_BINDING_TRANSCRIPTION_FACTOR_ACTIVITY	5.95E-09
GOBP_REGULATION_OF_VESICLE_MEDIATED_TRANSPORT	6.37E-09
GOBP_DNA_TEMPLATED_TRANSCRIPTION_INITIATION	6.49E-09
GOBP_REGULATION_OF_PROTEIN_DEPHOSPHORYLATION	6.59E-09
GOBP_VIRAL_GENE_EXPRESSION	6.65E-09
GOBP_TAXIS	6.75E-09
GOBP_REGULATION_OF_NUCLEOCYTOPLASMIC_TRANSPORT	6.94E-09
GOBP_REGULATION_OF_MRNA_METABOLIC_PROCESS	7.34E-09
GOBP_MONONUCLEAR_CELL_MIGRATION	8.22E-09
GOBP_EXTRINSIC_APOPTOTIC_SIGNALING_PATHWAY	8.23E-09
GOBP_RESPONSE_TO_TUMOR_NECROSIS_FACTOR	8.36E-09
GOBP_TELENCEPHALON_DEVELOPMENT	8.60E-09
GOBP_DEVELOPMENT_OF_PRIMARY_SEXUAL_CHARACTERISTICS	8.68E-09
GOBP_PROTEIN_TARGETING_TO_MITOCHONDRION	8.74E-09
GOBP_REGULATION_OF_CHEMOTAXIS	8.77E-09
GOBP_REGULATION_OF_PROTEOLYSIS_INVOLVED_IN_PROTEIN_CATABOLIC_PROCESS	8.88E-09
GOBP_ENDOSOME_ORGANIZATION	9.31E-09
GOBP_CARBOHYDRATE_CATABOLIC_PROCESS	9.44E-09
GOBP_REGULATION_OF_CELL_CYCLE_PHASE_TRANSITION	1.00E-08
GOBP_METHYLATION	1.02E-08
GOBP_TRANSLATIONAL_INITIATION	1.02E-08
GOBP_REGENERATION	1.22E-08
GOBP_REGULATION_OF_NERVOUS_SYSTEM_DEVELOPMENT	1.32E-08
GOBP_DNA_TEMPLATED_TRANSCRIPTION_ELONGATION	1.36E-08
GOBP_RESPONSE_TO_REACTIVE_OXYGEN_SPECIES	1.43E-08
GOBP_RAS_PROTEIN_SIGNAL_TRANSDUCTION	1.47E-08
GOBP_NEGATIVE_REGULATION_OF_CELLULAR_CATABOLIC_PROCESS	1.53E-08
GOBP_DEFENSE_RESPONSE_TO_BACTERIUM	1.59E-08
GOBP_POSITIVE_REGULATION_OF_INFLAMMATORY_RESPONSE	1.61E-08
GOBP_REGULATION_OF_PROTEIN_POLYMERIZATION	1.66E-08
GOBP_REGULATION_OF_LEUKOCYTE_PROLIFERATION	1.73E-08
GOBP_RECEPTOR_MEDIATED_ENDOCYTOSIS	1.76E-08

GOBP_DNA_MODIFICATION	1.76E-08
GOBP_EMBRYONIC_MORPHOGENESIS	1.87E-08
GOBP_POSITIVE_REGULATION_OF_INTRACELLULAR_PROTEIN_TRANSPORT	1.88E-08
GOBP_VESICLE_LOCALIZATION	1.89E-08
GOBP_PEPTIDYL_TYROSINE_MODIFICATION	1.95E-08
GOBP_RESPONSE_TO_CARBOHYDRATE	1.96E-08
GOBP_SEX_DIFFERENTIATION	1.98E-08
GOBP_PROTEIN_POLYMERIZATION	2.03E-08
GOBP_REGULATION_OF_B_CELL_ACTIVATION	2.06E-08
GOBP_REGULATION_OF_INTRACELLULAR_TRANSPORT	2.08E-08
GOBP_NEGATIVE_REGULATION_OF_HYDROLASE_ACTIVITY	2.16E-08
GOBP_MYELOID_CELL_HOMEOSTASIS	2.21E-08
GOBP_POSITIVE_REGULATION_OF_LEUKOCYTE_PROLIFERATION	2.32E-08
GOBP_SMALL_GTPASE_MEDIATED_SIGNAL_TRANSDUCTION	2.41E-08
GOBP_MONOATOMIC_ION_HOMEOSTASIS	2.65E-08
GOBP_VASCULATURE_DEVELOPMENT	2.68E-08
GOBP_MONOSACCHARIDE_METABOLIC_PROCESS	2.88E-08
GOBP_REGULATION_OF_ACTIN_FILAMENT_BASED_PROCESS	2.93E-08
GOBP_RESPONSE_TO_INORGANIC_SUBSTANCE	3.09E-08
GOBP_FATTY_ACID_METABOLIC_PROCESS	3.72E-08
GOBP_EMBRYO_DEVELOPMENT_ENDING_IN_BIRTH_OR_EGG_HATCHING	3.97E-08
GOBP_REGULATION_OF_CELL_GROWTH	3.97E-08
GOBP_REGULATION_OF_ENDOCYTOSIS	4.35E-08
GOBP_REGULATION_OF_HORMONE_SECRETION	4.74E-08
GOBP_CYTOKINE_MEDIATED_SIGNALING_PATHWAY	4.77E-08
GOBP_REGULATION_OF_STEM_CELL_DIFFERENTIATION	5.11E-08
GOBP_NUCLEUS_ORGANIZATION	5.19E-08
GOBP_HETEROCHROMATIN_ORGANIZATION	5.51E-08
GOBP_TISSUE_HOMEOSTASIS	5.92E-08
GOBP_INTRINSIC_APOPTOTIC_SIGNALING_PATHWAY_IN_RESPONSE_TO_DNA_DAMAGE	6.31E-08
GOBP_VESICLE_ORGANIZATION	6.34E-08
GOBP_SMALL_MOLECULE_CATABOLIC_PROCESS	6.45E-08
GOBP_SKIN_DEVELOPMENT	6.47E-08
GOBP_PEPTIDYL_LYSINE_ACETYLATION	6.88E-08
GOBP_REGULATION_OF_ENDOTHELIAL_CELL_MIGRATION	7.02E-08
GOBP_RESPONSE_TO_TOXIC_SUBSTANCE	7.41E-08
GOBP_POSITIVE_REGULATION_OF_PROTEIN_SERINE_THREONINE_KINASE_ACTIVITY	7.97E-08
GOBP_TRANSCRIPTION_INITIATION_AT_RNA_POLYMERASE_II_PROMOTER	8.69E-08
GOBP_RESPONSE_TO_ALCOHOL	8.99E-08
GOBP_ERAD_PATHWAY	1.03E-07
GOBP_PROTEIN_COMPLEX_OLIGOMERIZATION	1.12E-07
GOBP_MRNA_CATABOLIC_PROCESS	1.12E-07
GOBP_NEGATIVE_REGULATION_OF_PROTEIN_CONTAINING_COMPLEX_ASSEMBLY	1.15E-07
GOBP_REGULATION_OF_SUPRAMOLECULAR_FIBER_ORGANIZATION	1.28E-07
GOBP_BLOOD_VESSEL_MORPHOGENESIS	1.29E-07
GOBP_CARBOHYDRATE_HOMEOSTASIS	1.31E-07
GOBP_NEGATIVE_REGULATION_OF_LYMPHOCYTE_ACTIVATION	1.37E-07
GOBP_RESPONSE_TO_INSULIN	1.37E-07
GOBP_SENSORY_ORGAN_DEVELOPMENT	1.56E-07
GOBP_STRESS_ACTIVATED_PROTEIN_KINASE_SIGNALING_CASCADE	1.58E-07
GOBP_ERYTHROCYTE_HOMEOSTASIS	1.60E-07
GOBP_HEART_DEVELOPMENT	1.61E-07
GOBP_TISSUE_MORPHOGENESIS	1.74E-07
GOBP_T_CELL_DIFFERENTIATION	1.75E-07
GOBP_RESPONSE_TO_IONIZING_RADIATION	1.76E-07
GOBP_HEMOSTASIS	1.80E-07
GOBP_EPITHELIAL_TO_MESENCHYMAL_TRANSITION	1.94E-07
GOBP_PALLIUM_DEVELOPMENT	2.08E-07
GOBP_SYNAPSE_ORGANIZATION	2.33E-07
GOBP_MITOTIC_CELL_CYCLE_PHASE_TRANSITION	2.34E-07
GOBP_PLASMA_MEMBRANE_ORGANIZATION	2.36E-07
GOBP_CELLULAR_COMPONENT_MORPHOGENESIS	2.54E-07
GOBP_CIRCULATORY_SYSTEM_PROCESS	2.61E-07
GOBP_ORGANIC_ACID_BIOSYNTHETIC_PROCESS	2.67E-07

GOBP_RNA_MODIFICATION	2.73E-07
GOBP_REGULATION_OF_CELL_CYCLE_G1_S_PHASE_TRANSITION	2.96E-07
GOBP_SENSORY_SYSTEM_DEVELOPMENT	3.39E-07
GOBP_PROTEIN_HOMOOLIGOMERIZATION	3.64E-07
GOBP_GAMETE_GENERATION	3.77E-07
GOBP_MUSCLE_STRUCTURE_DEVELOPMENT	3.92E-07
GOBP_REGULATION_OF_PROTEASOMAL_PROTEIN_CATABOLIC_PROCESS	3.98E-07
GOBP_MITOCHONDRIAL_MEMBRANE_ORGANIZATION	3.99E-07
GOBP_MICROTUBULE_ORGANIZING_CENTER_ORGANIZATION	4.24E-07
GOBP_ENZYME_LINKED_RECEPTOR_PROTEIN_SIGNALING_PATHWAY	4.31E-07
GOBP_RESPONSE_TO_MONOSACCHARIDE	5.09E-07
GOBP_REGULATION_OF_PATTERN_RECOGNITION_RECEPTOR_SIGNALING_PATHWAY	5.22E-07
GOBP_SOMATIC_DIVERSIFICATION_OF_IMMUNE_RECEPTORS	5.35E-07
GOBP_ATP_SYNTHESIS_COUPLED_ELECTRON_TRANSPORT	5.51E-07
GOBP_EPIDERMAL_CELL_DIFFERENTIATION	5.78E-07
GOBP_MITOCHONDRIAL_TRANSPORT	5.84E-07
GOBP_POSITIVE_REGULATION_OF_NF_KAPPAB_TRANSCRIPTION_FACTOR_ACTIVITY	5.95E-07
GOBP_REGULATION_OF_DNA_TEMPLATED_TRANSCRIPTION_INITIATION	5.98E-07
GOBP_CILIUM_ORGANIZATION	6.04E-07
GOBP_CELL_FATE_COMMITMENT	6.33E-07
GOBP_ORGANIC_HYDROXY_COMPOUND_TRANSPORT	6.39E-07
GOBP_PYRUVATE_METABOLIC_PROCESS	6.56E-07
GOBP_REGULATION_OF_INTRACELLULAR_PROTEIN_TRANSPORT	6.61E-07
GOBP_MALE_GAMETE_GENERATION	6.73E-07
GOBP_AXO_DENDRITIC_TRANSPORT	6.75E-07
GOBP_ELECTRON_TRANSPORT_CHAIN	7.23E-07
GOBP_EPITHELIAL_CELL_APOPTOTIC_PROCESS	7.32E-07
GOBP_REGULATION_OF_DNA_TEMPLATED_TRANSCRIPTION_ELONGATION	7.88E-07
GOBP_MAINTENANCE_OF_LOCATION	8.57E-07
GOBP_REGULATION_OF_MITOTIC_CELL_CYCLE_PHASE_TRANSITION	9.09E-07
GOBP_DEPHOSPHORYLATION	9.18E-07
GOBP_REGULATION_OF_ANATOMICAL_STRUCTURE_SIZE	9.31E-07
GOBP_ENDOMEMBRANE_SYSTEM_ORGANIZATION	1.05E-06
GOBP_NEGATIVE_REGULATION_OF_IMMUNE_RESPONSE	1.06E-06
GOBP_REGULATION_OF_MICROTUBULE_BASED_PROCESS	1.07E-06
GOBP_MAINTENANCE_OF_LOCATION_IN_CELL	1.09E-06
GOBP_LIPID_BIOSYNTHETIC_PROCESS	1.10E-06
GOBP_POSITIVE_REGULATION_OF_PROTEIN_CONTAINING_COMPLEX_ASSEMBLY	1.15E-06
GOBP_RESPIRATORY_ELECTRON_TRANSPORT_CHAIN	1.17E-06
GOBP_REGULATION_OF_DNA_BINDING_TRANSCRIPTION_FACTOR_ACTIVITY	1.25E-06
GOBP_REGULATION_OF_EXTRINSIC_APOPTOTIC_SIGNALING_PATHWAY	1.29E-06
GOBP_CALCIUM_ION_HOMEOSTASIS	1.34E-06
GOBP_NEGATIVE_REGULATION_OF_DNA_METABOLIC_PROCESS	1.38E-06
GOBP_HEART_MORPHOGENESIS	1.40E-06
GOBP_REGULATORY_NCRNA_MEDIATED_GENE_SILENCING	1.44E-06
GOBP_POSITIVE_REGULATION_OF_GROWTH	1.47E-06
GOBP_SPLICEOSOMAL_COMPLEX_ASSEMBLY	1.58E-06
GOBP_SIGNAL_RELEASE	1.58E-06
GOBP_GLUCOSE_METABOLIC_PROCESS	1.61E-06
GOBP_MESENCHYMAL_CELL_DIFFERENTIATION	1.70E-06
GOBP_REGULATION_OF_PROTEOLYSIS	1.81E-06
GOBP_ORGANELLE_MEMBRANE_FUSION	1.85E-06
GOBP_SMALL_MOLECULE_BIOSYNTHETIC_PROCESS	1.93E-06
GOBP_GLYCEROLIPID_METABOLIC_PROCESS	2.35E-06
GOBP_MICROTUBULE_BASED_MOVEMENT	2.41E-06
GOBP_REGULATION_OF_TRANSPORTER_ACTIVITY	2.78E-06
GOBP_AMEBOIDAL_TYPE_CELL_MIGRATION	3.07E-06
GOBP_ACTIN_POLYMERIZATION_OR_DEPOLYMERIZATION	3.44E-06
GOBP_POSITIVE_REGULATION_OF_MAPK_CASCADE	3.50E-06
GOBP_HORMONE_TRANSPORT	3.77E-06
GOBP_PROTEIN_MODIFICATION_BY_SMALL_PROTEIN_CONJUGATION	3.95E-06
GOBP_STEROID_HORMONE_MEDIATED_SIGNALING_PATHWAY	3.98E-06
GOBP_REGULATION_OF_MITOCHONDRION_ORGANIZATION	4.21E-06
GOBP_CELL_PROJECTION_ASSEMBLY	4.50E-06

GOBP_NUCLEAR_TRANSCRIBED_MRNA_CATABOLIC_PROCESS	5.08E-06
GOBP_CYTOSOLIC_TRANSPORT	5.11E-06
GOBP_ESTABLISHMENT_OF_CELL_POLARITY	5.55E-06
GOBP_ACTIN_FILAMENT_BUNDLE_ORGANIZATION	5.87E-06
GOBP_REGULATION_OF_PEPTIDYL_SERINE_PHOSPHORYLATION	6.61E-06
GOBP_EPIDERMIS_DEVELOPMENT	7.44E-06
GOBP_REGULATION_OF_MONOATOMIC_ION_TRANSPORT	7.45E-06
GOBP_REGULATION_OF_EPITHELIAL_CELL_MIGRATION	8.95E-06
GOBP_NATURAL_KILLER_CELL_ACTIVATION	9.53E-06
GOBP_NEGATIVE_REGULATION_OF_RESPONSE_TO_BIOTIC_STIMULUS	9.67E-06
GOBP_MYOBLAST_DIFFERENTIATION	1.02E-05
GOBP_NUCLEOSIDE_TRIPHOSPHATE_BIOSYNTHETIC_PROCESS	1.02E-05
GOBP_PEPTIDYL_LYSINE_MODIFICATION	1.07E-05
GOBP_MULTI_MULTICELLULAR_ORGANISM_PROCESS	1.09E-05
GOBP_RNA_EXPORT_FROM_NUCLEUS	1.11E-05
GOBP_VESICLE_TARGETING	1.16E-05
GOBP_GASTRULATION	1.20E-05
GOBP_REGULATION_OF_MONOATOMIC_CATION_TRANSMEMBRANE_TRANSPORT	1.21E-05
GOBP_HORMONE_MEDIATED_SIGNALING_PATHWAY	1.21E-05
GOBP_REGULATION_OF_CELL_DIVISION	1.27E-05
GOBP_ALCOHOL_METABOLIC_PROCESS	1.30E-05
GOBP_MUSCLE_CELL_DIFFERENTIATION	1.39E-05
GOBP_NEGATIVE_REGULATION_OF_ORGANELLE_ORGANIZATION	1.45E-05
GOBP_REGULATION_OF_SISTER_CHROMATID_SEGREGATION	1.46E-05
GOBP_NEGATIVE_REGULATION_OF_GROWTH	1.49E-05
GOBP_PROTEIN_LOCALIZATION_TO_CELL_PERIPHERY	1.50E-05
GOBP_REGULATION_OF_RNA_SPLICING	1.55E-05
GOBP_RESPONSE_TO_TYPE_II_INTERFERON	1.59E-05
GOBP_PROTEIN_LOCALIZATION_TO_VACUOLE	1.60E-05
GOBP_POSITIVE_REGULATION_OF_LEUKOCYTE_MIGRATION	1.67E-05
GOBP_REGULATION_OF_VIRAL_PROCESS	1.68E-05
GOBP_NEGATIVE_REGULATION_OF_TRANSPORT	1.78E-05
GOBP_INTRINSIC_APOPTOTIC_SIGNALING_PATHWAY_BY_P53_CLASS_MEDIATOR	1.80E-05
GOBP_PROTEIN_LOCALIZATION_TO_EXTRACELLULAR_REGION	1.85E-05
GOBP_NEGATIVE_REGULATION_OF_CYTOSKELETON_ORGANIZATION	1.93E-05
GOBP_HEART_PROCESS	2.08E-05
GOBP_MAINTENANCE_OF_PROTEIN_LOCATION	2.15E-05
GOBP_BEHAVIOR	2.20E-05
GOBP_HINDBRAIN_DEVELOPMENT	2.31E-05
GOBP_POSITIVE_REGULATION_OF_AUTOPHAGY	2.43E-05
GOBP_MUSCLE_CONTRACTION	2.81E-05
GOBP_RESPONSE_TO_BACTERIUM	2.82E-05
GOBP_AMINO_ACID_METABOLIC_PROCESS	3.05E-05
GOBP_BONE_DEVELOPMENT	3.30E-05
GOBP_REGULATION_OF_CELL_SHAPE	3.69E-05
GOBP_EPITHELIAL_TUBE_MORPHOGENESIS	3.69E-05
GOBP_MESENCHYME_DEVELOPMENT	3.86E-05
GOBP_CELLULAR_RESPONSE_TO_STARVATION	4.13E-05
GOBP_REGULATION_OF_MONOATOMIC_ION_TRANSMEMBRANE_TRANSPORT	4.30E-05
GOBP_NEGATIVE_REGULATION_OF_INTRINSIC_APOPTOTIC_SIGNALING_PATHWAY	4.38E-05
GOBP_ENDOTHELIAL_CELL_MIGRATION	4.43E-05
GOBP_NEGATIVE_REGULATION_OF_CELL_PROJECTION_ORGANIZATION	4.79E-05
GOBP_REGULATION_OF_CIRCADIAN_RHYTHM	4.79E-05
GOBP_POSITIVE_REGULATION_OF_HYDROLASE_ACTIVITY	5.14E-05
GOBP_CELL_MORPHOGENESIS_INVOLVED_IN_NEURON_DIFFERENTIATION	5.21E-05
GOBP_REGULATION_OF_METAL_ION_TRANSPORT	5.32E-05
GOBP_PHOSPHATIDYLINOSITOL_3_KINASE_PROTEIN_KINASE_B_SIGNAL_TRANSDUCTION	5.53E-05
GOBP_MYELOID_LEUKOCYTE_MEDIATED_IMMUNITY	5.60E-05
GOBP_NEGATIVE_REGULATION_OF_RNA_CATABOLIC_PROCESS	5.65E-05
GOBP_POSITIVE_REGULATION_OF_PEPTIDYL_TYROSINE_PHOSPHORYLATION	5.75E-05
GOBP_VESICLE_MEDIATED_TRANSPORT_TO_THE_PLASMA_MEMBRANE	5.84E-05
GOBP_RESPONSE_TO_METAL_ION	5.99E-05
GOBP_DEVELOPMENTAL_GROWTH_INVOLVED_IN_MORPHOGENESIS	6.81E-05
GOBP_CELLULAR_RESPONSE_TO_REACTIVE_OXYGEN_SPECIES	6.92E-05

GOBP_REGULATION_OF_LIPID_BIOSYNTHETIC_PROCESS	7.13E-05
GOBP_POSITIVE_REGULATION_OF_PROTEOLYSIS_INVOLVED_IN_PROTEIN_CATABOLIC_PROCESS	7.23E-05
GOBP_RIBOSE_PHOSPHATE_BIOSYNTHETIC_PROCESS	7.45E-05
GOBP_SYNAPTIC_SIGNALING	7.63E-05
GOBP_STRIATED_MUSCLE_CELL_DIFFERENTIATION	8.22E-05
GOBP_POSITIVE_REGULATION_OF_DOUBLE_STRAND_BREAK_REPAIR	8.30E-05
GOBP_NEGATIVE_REGULATION_OF_AMIDE_METABOLIC_PROCESS	9.39E-05
GOBP_REGULATION_OF_LEUKOCYTE_MIGRATION	9.44E-05
GOBP_MICROTUBULE_BASED_TRANSPORT	9.73E-05
GOBP_CYTOSKELETON_DEPENDENT_INTRACELLULAR_TRANSPORT	9.81E-05
GOBP_TYPE_I_INTERFERON_PRODUCTION	0.0001011
GOBP_UBIQUITIN_DEPENDENT_ERAD_PATHWAY	0.00010207
GOBP_LEUKOCYTE_HOMEOSTASIS	0.00010763
GOBP_CELL_PART_MORPHOGENESIS	0.0001153
GOBP_POSITIVE_REGULATION_OF_PROTEASOMAL_PROTEIN_CATABOLIC_PROCESS	0.00012475
GOBP_INTERLEUKIN_6_PRODUCTION	0.00013481
GOBP_NEGATIVE_REGULATION_OF_CELL_CYCLE_PROCESS	0.00013536
GOBP_T_CELL_PROLIFERATION	0.00013828
GOBP_NEGATIVE_REGULATION_OF_PROTEIN_CATABOLIC_PROCESS	0.0001404
GOBP_MEMBRANE_DOCKING	0.00014042
GOBP_EXOCYTOSIS	0.00015738
GOBP_MORPHOGENESIS_OF_AN_EPITHELIUM	0.00016973
GOBP_TORC1_SIGNALING	0.00016992
GOBP_POSITIVE_REGULATION_OF_CELL_SUBSTRATE_ADHESION	0.00018142
GOBP_PROTEIN_CONTAINING_COMPLEX_LOCALIZATION	0.0001823
GOBP_BLOOD_VESSEL_ENDOTHELIAL_CELL_MIGRATION	0.00021286
GOBP_CALCIUM_MEDIATED_SIGNALING	0.00021917
GOBP_NEGATIVE_REGULATION_OF_CELL_DIFFERENTIATION	0.00025419
GOBP_VACUOLE_ORGANIZATION	0.00025513
GOBP_AMIDE_TRANSPORT	0.00026728
GOBP_CYTOSKELETON_DEPENDENT_CYTOKINESIS	0.00026962
GOBP_TRANSPORT_ALONG_MICROTUBULE	0.00027617
GOBP_IN_UTERO_EMBRYONIC_DEVELOPMENT	0.00032548
GOBP_REGULATION_OF_TRANSCRIPTION_ELONGATION_BY_RNA_POLYMERASE_II	0.00032902
GOBP_MYELOID_LEUKOCYTE_DIFFERENTIATION	0.00036458
GOBP_REGULATION_OF_DOUBLE_STRAND_BREAK_REPAIR	0.0003706
GOBP_NEURAL_TUBE_DEVELOPMENT	0.00038146
GOBP_REGULATION_OF_CELLULAR_COMPONENT_SIZE	0.00038527
GOBP_TRANSMEMBRANE_RECEPTOR_PROTEIN_SERINE_THREONINE_KINASE_SIGNALING_PATHWAY	0.00039715
GOBP_MIRNA_METABOLIC_PROCESS	0.00039945
GOBP_GLIAL_CELL_DIFFERENTIATION	0.00042313
GOBP_PROTEIN_K48_LINKED_UBIQUITINATION	0.00043758
GOBP_TUBE_FORMATION	0.00043946
GOBP_RESPONSE_TO_SALT	0.00044
GOBP_REGULATION_OF_TRANSMEMBRANE_RECEPTOR_PROTEIN_SERINE_THREONINE_KINASE_SIGNALING_PATHWAY	0.00047949
GOBP_ENDOCYTIC_RECYCLING	0.00049599
GOBP_REGULATION_OF_CELLULAR_RESPONSE_TO_TRANSFORMING_GROWTH_FACTOR_BETA_STIMULUS	0.00050649
GOBP_POSITIVE_REGULATION_OF_NEUROGENESIS	0.00052758
GOBP_TOR_SIGNALING	0.0005349
GOBP_CELL_CYCLE_G2_M_PHASE_TRANSITION	0.00053616
GOBP_B_CELL_MEDIATED_IMMUNITY	0.00054456
GOBP_REGULATION_OF_SECRETION	0.00057073
GOBP_REGULATION_OF_MAP_KINASE_ACTIVITY	0.00057381
GOBP_APOPTOTIC_MITOCHONDRIAL_CHANGES	0.00058434
GOBP_REGULATION_OF_CYSTEINE_TYPE_ENDOPEPTIDASE_ACTIVITY_INVOLVED_IN_APOPTOTIC_PROCESS	0.00058903
GOBP_DENDRITE_MORPHOGENESIS	0.00059026
GOBP_CYTOKINESIS	0.00060932
GOBP_REGULATION_OF_CELLULAR_RESPONSE_TO_GROWTH_FACTOR_STIMULUS	0.00063383
GOBP_AUTOPHAGY_OF_MITOCHONDRION	0.00071112
GOBP_CELL_CYCLE_G1_S_PHASE_TRANSITION	0.00073717
GOBP_REGULATION_OF_MICROTUBULE_CYTOSKELETON_ORGANIZATION	0.00074808
GOBP_ADAPTIVE_THERMOGENESIS	0.00077493
GOBP_REGULATION_OF_CYSTEINE_TYPE_ENDOPEPTIDASE_ACTIVITY	0.00079189
GOBP_PROTEIN_TARGETING_TO_MEMBRANE	0.00084628

GOBP_CALCIUM_ION_TRANSPORT	0.00084658
GOBP_TISSUE_MIGRATION	0.00085779
GOBP_PEPTIDE_TRANSPORT	0.00088169
GOBP_REGULATION_OF_DNA_REPAIR	0.00088869
GOBP_PROTEIN_LOCALIZATION_TO_PLASMA_MEMBRANE	0.00089417
GOBP_LYTIC_VACUOLE_ORGANIZATION	0.00092928
GOBP_POSITIVE_REGULATION_OF_APOPTOTIC_SIGNALING_PATHWAY	0.0009377
GOBP_REGULATION_OF_MRNA_CATABOLIC_PROCESS	0.00095788
GOBP_REGULATION_OF_WNT_SIGNALING_PATHWAY	0.00097796
GOBP_POSITIVE_REGULATION_OF_VIRAL_PROCESS	0.00108481
GOBP_VASCULAR_PROCESS_IN_CIRCULATORY_SYSTEM	0.00108827
GOBP_REGULATION_OF_ORGANELLE_ASSEMBLY	0.00112407
GOBP_POSITIVE_REGULATION_OF_DNA_REPAIR	0.00122272
GOBP_AXON_DEVELOPMENT	0.00129446
GOBP_REGULATION_OF_LEUKOCYTE_APOPTOTIC_PROCESS	0.0015417
GOBP_POSITIVE_REGULATION_OF_NERVOUS_SYSTEM_DEVELOPMENT	0.00170546
GOBP_REGULATION_OF_TRANSMEMBRANE_TRANSPORT	0.00171815
GOBP_ATP_BIOSYNTHETIC_PROCESS	0.00181587
GOBP_GLYCOPROTEIN_METABOLIC_PROCESS	0.00196037
GOBP_CALCIUM_ION_TRANSMEMBRANE_IMPORT_INTO_CYTOSOL	0.00199431
GOBP_INSULIN_RECEPTOR_SIGNALING_PATHWAY	0.00204639
GOBP_NEGATIVE_REGULATION_OF_LOCOMOTION	0.00212703
GOBP_RESPONSE_TO_MOLECULE_OF_BACTERIAL_ORIGIN	0.00241301
GOBP_REGULATION_OF_GENERATION_OF_PRECURSOR_METABOLITES_AND_ENERGY	0.00250774
GOBP_STRIATED_MUSCLE_TISSUE_DEVELOPMENT	0.00265921
GOBP_ERK1_AND_ERK2_CASCADE	0.00271552
GOBP_AUTOPHAGOSOME_ORGANIZATION	0.00274562
GOBP_ESTABLISHMENT_OF_PROTEIN_LOCALIZATION_TO_MEMBRANE	0.00315145
GOBP_ORGANIC_ANION_TRANSPORT	0.00321194
GOBP_GLIOGENESIS	0.00326496
GOBP_GLYCEROPHOSPHOLIPID_METABOLIC_PROCESS	0.00334911
GOBP_NEGATIVE_REGULATION_OF_CELL_GROWTH	0.00389762
GOBP_TUMOR_NECROSIS_FACTOR_MEDIATED_SIGNALING_PATHWAY	0.00458876
GOBP_MYELOID_LEUKOCYTE_MIGRATION	0.00461359
GOBP_HEMATOPOIETIC_PROGENITOR_CELL_DIFFERENTIATION	0.00482965
GOBP_REGULATION_OF_MYELOID_CELL_DIFFERENTIATION	0.00488507
GOBP_PROTEIN_AUTOPHOSPHORYLATION	0.00521367
GOBP_STEROID_METABOLIC_PROCESS	0.00563387
GOBP_REGULATION_OF_REPRODUCTIVE_PROCESS	0.00566844
GOBP_REGULATION_OF_SYSTEM_PROCESS	0.00587372
GOBP_CELLULAR_RESPONSE_TO_INORGANIC_SUBSTANCE	0.00614767
GOBP_SENSORY_ORGAN_MORPHOGENESIS	0.00669071
GOBP_FAT_CELL_DIFFERENTIATION	0.00765124
GOBP_GOLGI_VESICLE_TRANSPORT	0.00807972
GOBP_MUSCLE_CELL_DEVELOPMENT	0.00808373
GOBP_CALCIUM_ION_TRANSMEMBRANE_TRANSPORT	0.00841335
GOBP_NEGATIVE_REGULATION_OF_MITOTIC_CELL_CYCLE_PHASE_TRANSITION	0.00868115
GOBP_ENDOPLASMIC_RETICULUM_ORGANIZATION	0.00884623
GOBP_REGULATION_OF_NEURON_DIFFERENTIATION	0.00967775
GOBP_PEPTIDYL_THREONINE_MODIFICATION	0.01068901
GOBP_PHOSPHOLIPID_METABOLIC_PROCESS	0.0108917
GOBP_VESICLE_BUDDING_FROM_MEMBRANE	0.01186416
GOBP_REGULATION_OF_PROTEIN_LOCALIZATION_TO_MEMBRANE	0.01323011
GOBP_CELL_CELL_SIGNALING_BY_WNT	0.01351664
GOBP_INSULIN_SECRETION	0.01381646
GOBP_POSITIVE_REGULATION_OF_MRNA_METABOLIC_PROCESS	0.01433244
GOBP_MITOTIC_CELL_CYCLE_CHECKPOINT_SIGNALING	0.01484766
GOBP_MUSCLE_SYSTEM_PROCESS	0.01517471
GOBP_REGULATION_OF_RESPONSE_TO_CYTOKINE_STIMULUS	0.0161286
GOBP_NEGATIVE_REGULATION_OF_SECRETION	0.01710051
GOBP_CELL_CYCLE_CHECKPOINT_SIGNALING	0.01718039
GOBP_LOCOMOTORY_BEHAVIOR	0.01730302
GOBP_MUSCLE_TISSUE_DEVELOPMENT	0.02095661
GOBP_POSITIVE_REGULATION_OF_MONOATOMIC_ION_TRANSPORT	0.02191988

GOBP_MULTICELLULAR_ORGANISM_GROWTH	0.02239709
GOBP_STEROL_METABOLIC_PROCESS	0.02246025
GOBP_MUSCLE_ORGAN_DEVELOPMENT	0.02321256
GOBP_MITOTIC_CYTOKINESIS	0.02368944
GOBP_POSITIVE_REGULATION_OF_CELL_GROWTH	0.02421998
GOBP_REGULATION_OF_PROTEIN_LOCALIZATION_TO_PLASMA_MEMBRANE	0.02892262
GOBP_DEVELOPMENTAL_GROWTH	0.02900511
GOBP_TUMOR_NECROSIS_FACTOR_SUPERFAMILY_CYTOKINE_PRODUCTION	0.03037864
GOBP_REGULATION_OF_EXOCYTOSIS	0.0345556
GOBP_REGULATED_EXOCYTOSIS	0.03583179
GOBP_POSITIVE_REGULATION_OF_BINDING	0.03673433
GOBP_TEMPERATURE_HOMEOSTASIS	0.03888702
GOBP_CELL_JUNCTION_ASSEMBLY	0.04114805
GOBP_REGULATION_OF_ACTIN_FILAMENT_BUNDLE_ASSEMBLY	0.04249202
GOBP_CELL_CELL_JUNCTION_ORGANIZATION	0.0444231
GOBP_MALE_SEX_DIFFERENTIATION	0.04748432
GOBP_RETROGRADE_TRANSPORT_ENDOSOME_TO_GOLGI	0.0537013
GOBP_ENSHEATHMENT_OF_NEURONS	0.05438723
GOBP_RESPIRATORY_SYSTEM_DEVELOPMENT	0.05727668
GOBP_NEGATIVE_REGULATION_OF_MITOTIC_CELL_CYCLE	0.05743212
GOBP_REGULATION_OF_GTPASE_ACTIVITY	0.06021952
GOBP_STRESS_FIBER_ASSEMBLY	0.06090814
GOBP_POSTSYNAPSE_ORGANIZATION	0.06624803
GOBP_REGULATION_OF_CELL_PROJECTION_ORGANIZATION	0.06888475
GOBP_ORGANIC_ACID_TRANSPORT	0.06897001
GOBP_NEGATIVE_REGULATION_OF_INNATE_IMMUNE_RESPONSE	0.07014744
GOBP_REGULATION_OF_UBIQUITIN_DEPENDENT_PROTEIN_CATABOLIC_PROCESS	0.07078057
GOBP_POSITIVE_REGULATION_OF_PROTEIN_LOCALIZATION_TO_MEMBRANE	0.07197405
GOBP_EMBRYONIC_ORGAN_MORPHOGENESIS	0.0741126
GOBP_SPINDLE_ASSEMBLY	0.074307
GOBP_VESICLE_MEDIATED_TRANSPORT_IN_SYNAPSE	0.0775767
GOBP_REACTIVE_OXYGEN_SPECIES_METABOLIC_PROCESS	0.07786183
GOBP_MRNA_TRANSPORT	0.08339226
GOBP_PROTON_MOTIVE_FORCE_DRIVEN_ATP_SYNTHESIS	0.08705689
GOBP_EMBRYONIC_ORGAN_DEVELOPMENT	0.08764979
GOBP_NEGATIVE_REGULATION_OF_SUPRAMOLECULAR_FIBER_ORGANIZATION	0.08932634
GOBP_RNA_DESTABILIZATION	0.08985191
GOBP_HEMATOPOIETIC_OR_LYMPHOID_ORGAN_DEVELOPMENT	0.09665317
GOBP_REGULATION_OF_STRESS_ACTIVATED_PROTEIN_KINASE_SIGNALING_CASCADE	0.09675123
GOBP_REGULATION_OF_BINDING	0.09736035
GOBP_PROTEIN_DEPHOSPHORYLATION	0.11064006
GOBP_MICROTUBULE_POLYMERIZATION_OR_DEPOLYMERIZATION	0.1229837
GOBP_LIPID_CATABOLIC_PROCESS	0.12362704
GOBP_PROTEIN_LOCALIZATION_TO_ENDOPLASMIC_RETICULUM	0.12477351
GOBP_NEGATIVE_REGULATION_OF_PROTEIN_LOCALIZATION	0.12573512
GOBP_REGULATION_OF_ANTIGEN_RECEPTOR_MEDIATED_SIGNALING_PATHWAY	0.1340369
GOBP_REGULATION_OF_PROTEIN_LOCALIZATION_TO_CELL_PERIPHERY	0.14431521
GOBP_REGULATION_OF_REACTIVE_OXYGEN_SPECIES_METABOLIC_PROCESS	0.15178481
GOBP_DEVELOPMENTAL_CELL_GROWTH	0.15518391
GOBP_MEMBRANE_FUSION	0.16667569
GOBP_REGULATION_OF_CANONICAL_WNT_SIGNALING_PATHWAY	0.17334953
GOBP_PLACENTA_DEVELOPMENT	0.19330474
GOBP_POSITIVE_REGULATION_OF_EPITHELIAL_CELL_PROLIFERATION	0.19352556
GOBP_REGULATION_OF_CELL_PROJECTION_ASSEMBLY	0.21684937
GOBP_DNA_INTEGRITY_CHECKPOINT_SIGNALING	0.21933051
GOBP_REGULATION_OF_MICROTUBULE_POLYMERIZATION_OR_DEPOLYMERIZATION	0.2208515
GOBP_REGULATION_OF_CARBOHYDRATE_METABOLIC_PROCESS	0.22259244
GOBP_DENDRITE_DEVELOPMENT	0.22419941
GOBP_POSITIVE_REGULATION_OF_CELL_PROJECTION_ORGANIZATION	0.24306031
GOBP_SECOND_MESSENGER_MEDIATED_SIGNALING	0.26271215
GOBP_RNA_3_END_PROCESSING	0.27982362
GOBP_RECEPTOR_INTERNALIZATION	0.28442116
GOBP_REGULATION_OF_DEVELOPMENTAL_GROWTH	0.28768664
GOBP_POSITIVE_REGULATION_OF_TRANSMEMBRANE_TRANSPORT	0.31128259

GOBP_STEROID_BIOSYNTHETIC_PROCESS	0.32297853
GOBP_REGULATION_OF_PEPTIDASE_ACTIVITY	0.32642228
GOBP_MICROTUBULE_POLYMERIZATION	0.34255657
GOBP_REGULATION_OF_PROTEIN_TARGETING	0.35748999
GOBP_SKELETAL_MUSCLE_ORGAN_DEVELOPMENT	0.36277038
GOBP_CANONICAL_WNT_SIGNALING_PATHWAY	0.36766819
GOBP_NEGATIVE_REGULATION_OF_INFLAMMATORY_RESPONSE	0.36969182
GOBP_CARBOHYDRATE_BIOSYNTHETIC_PROCESS	0.39114673
GOBP_CELLULAR_RESPONSE_TO_TYPE_II_INTERFERON	0.40699406
GOBP_COGNITION	0.40875784
GOBP_CELLULAR_PROCESS_INVOLVED_IN_REPRODUCTION_IN_MULTICELLULAR_ORGANISM	0.42496286
GOBP_REGULATION_OF_CALCIUM_ION_TRANSPORT	0.43399062
GOBP_NEGATIVE_REGULATION_OF_TRANSMEMBRANE_RECEPTOR_PROTEIN_SERINE_THREONINE_ KINASE_SIGNALING_PATHWAY	0.45503828
GOBP_GLYCOPROTEIN_BIOSYNTHETIC_PROCESS	0.48276323
GOBP_REGULATION_OF_DNA_RECOMBINATION	0.49241989
GOBP_NEGATIVE_REGULATION_OF_MAPK_CASCADE	0.49853138
GOBP_MAINTENANCE_OF_CELL_NUMBER	0.50693341
GOBP_REGULATION_OF_SYNAPTIC_PLASTICITY	0.51588189
GOBP_REGULATION_OF_TRANS_SYNAPTIC_SIGNALING	0.52779887
GOBP_RECEPTOR_SIGNALING_PATHWAY_VIA_STAT	0.53065178
GOBP_ORGANIC_ACID_CATABOLIC_PROCESS	0.53316753
GOBP_MEMBRANE_LIPID_METABOLIC_PROCESS	0.55048447
GOBP_NUCLEAR_TRANSCRIBED_MRNA_CATABOLIC_PROCESS_DEADENYLATION_DEPENDENT_DECAY	0.57615808
GOBP_CELLULAR_KETONE_METABOLIC_PROCESS	0.62759869
GOBP_REGULATION_OF_CELL_CYCLE_G2_M_PHASE_TRANSITION	0.65756484
GOBP_RESPONSE_TO_NUTRIENT	0.65832423
GOBP_BLASTOCYST_DEVELOPMENT	0.67246747
GOBP_GLYCOSYLATION	0.67396748
GOBP_MRNA_EXPORT_FROM_NUCLEUS	0.67681011
GOBP_PROTEIN_MODIFICATION_BY_SMALL_PROTEIN_REMOVAL	0.69107885
GOBP_REGULATION_OF_PROTEASOMAL_UBIQUITIN_DEPENDENT_PROTEIN_CATABOLIC_PROCESS	0.70900785
GOBP_ENDOTHELIUM_DEVELOPMENT	0.71745116
GOBP_NEGATIVE_REGULATION_OF_ESTABLISHMENT_OF_PROTEIN_LOCALIZATION	0.72379231
GOBP_POSITIVE_REGULATION_OF_GTPASE_ACTIVITY	0.73306078
GOBP_DEVELOPMENTAL_MATURATION	0.75018982
GOBP_CELLULAR_RESPONSE_TO_TOPOLOGICALLY_INCORRECT_PROTEIN	0.77388225
GOBP_MITOCHONDRIAL_RESPIRATORY_CHAIN_COMPLEX_ASSEMBLY	0.78232733
GOBP_POSITIVE_REGULATION_OF_PROTEOLYSIS	0.78641607
GOBP_REGULATION_OF_NEURON_PROJECTION_DEVELOPMENT	0.79477511
GOBP_POSITIVE_REGULATION_OF_EPITHELIAL_CELL_MIGRATION	0.80157445
GOBP_LIPID_LOCALIZATION	0.82327125
GOBP_REGULATION_OF_MUSCLE_CELL_DIFFERENTIATION	0.85722429
GOBP_POSITIVE_REGULATION_OF_SECRETION	0.85867931
GOBP_ORGANELLE_TRANSPORT_ALONG_MICROTUBULE	0.86713701
GOBP_ORGAN_GROWTH	0.86903885
GOBP_REGULATION_OF_TRANSLATIONAL_INITIATION	0.87173572
GOBP_POSITIVE_REGULATION_OF_CYSTEINE_TYPE_ENDOPEPTIDASE_ACTIVITY	0.878294
GOBP_NEGATIVE_REGULATION_OF_BINDING	0.87939374
GOBP_GERM_CELL_DEVELOPMENT	0.88453105
GOBP_LAMELLIPODIUM_ORGANIZATION	0.89361889
GOBP_REGULATION_OF_JNK_CASCADE	0.91913758
GOBP_ORGANIC_HYDROXY_COMPOUND_BIOSYNTHETIC_PROCESS	0.95363967
GOBP_LEUKOCYTE_APOPTOTIC_PROCESS	0.96725128
GOBP_REGULATION_OF_CALCIUM_ION_TRANSMEMBRANE_TRANSPORT	0.96989247
GOBP_JNK_CASCADE	0.9738892
GOBP_REGULATION_OF_NON_CANONICAL_NF_KAPPAB_SIGNAL_TRANSDUCTION	0.97571849
GOBP_CELLULAR_LIPID_CATABOLIC_PROCESS	0.97933174
GOBP_POSITIVE_REGULATION_OF_PEPTIDASE_ACTIVITY	0.98851048
GOBP_REGULATION_OF_PROTEIN_BINDING	0.9889387
GOBP_RHO_PROTEIN_SIGNAL_TRANSDUCTION	0.99009131
GOBP_PATTERN_SPECIFICATION_PROCESS	0.99440222
GOBP_NEGATIVE_REGULATION_OF_DNA_BINDING_TRANSCRIPTION_FACTOR_ACTIVITY	0.99442167
GOBP_POSITIVE_REGULATION_OF_UBIQUITIN_DEPENDENT_PROTEIN_CATABOLIC_PROCESS	0.99544391

GOBP_LIPID_MODIFICATION	0.99647074
GOBP_CELLULAR_RESPONSE_TO_UNFOLDED_PROTEIN	0.99652508
GOBP_ENDOPLASMIC_RETICULUM_TO_GOLGI_VESICLE_MEDIATED_TRANSPORT	0.99753436
GOBP_REGULATION_OF_MYELOID_LEUKOCYTE_DIFFERENTIATION	0.99875028
GOBP_ANATOMICAL_STRUCTURE_MATURATION	0.99881367
GOBP_POSITIVE_REGULATION_OF_PROTEASOMAL_UBIQUITIN_DEPENDENT_PROTEIN_CATABOLIC_PROCESS	0.99932564
GOBP_POSITIVE_REGULATION_OF_NEURON_PROJECTION_DEVELOPMENT	0.99945418
GOBP_GOLGI_ORGANIZATION	0.99949839
GOBP_GLYCEROLIPID_BIOSYNTHETIC_PROCESS	0.999568
GOBP_ENDOPLASMIC_RETICULUM_UNFOLDED_PROTEIN_RESPONSE	0.99980125
GOBP_REGULATION_OF_MUSCLE_SYSTEM_PROCESS	0.99982424
GOBP_REGULATION_OF_PROTEIN_SECRETION	0.99983765
GOBP_EPITHELIAL_CELL_DEVELOPMENT	0.99986919
GOBP_SULFUR_COMPOUND_METABOLIC_PROCESS	0.99995348
GOBP_POST_EMBRYONIC_DEVELOPMENT	0.99995512
GOBP_GLYCEROPHOSPHOLIPID_BIOSYNTHETIC_PROCESS	0.99997771
GOBP_NON_CANONICAL_NF_KAPPAB_SIGNAL_TRANSDUCTION	0.99998314
GOBP_PHOSPHATIDYLINOSITOL_METABOLIC_PROCESS	0.99998864
GOBP_TISSUE_REMODELING	0.9999968
GOBP_POST_GOLGI_VESICLE_MEDIATED_TRANSPORT	0.9999994
GOBP_PHOSPHOLIPID_BIOSYNTHETIC_PROCESS	0.99999968
GOBP_REGULATION_OF_RESPONSE_TO_ENDOPLASMIC_RETICULUM_STRESS	0.99999991
GOBP_NOTCH_SIGNALING_PATHWAY	1
