# Supplementary figures and images for: GSNCASCR: An R Package to Identify Differentially Co-Expressed Curated Gene Sets with Single-Cell RNA-Seq Data
Source: Int J Mol Sci. 2025 May 16;26(10):4771. doi: 10.3390/ijms26104771 (PMC12112291; doi:10.3390/ijms26104771)

COVID

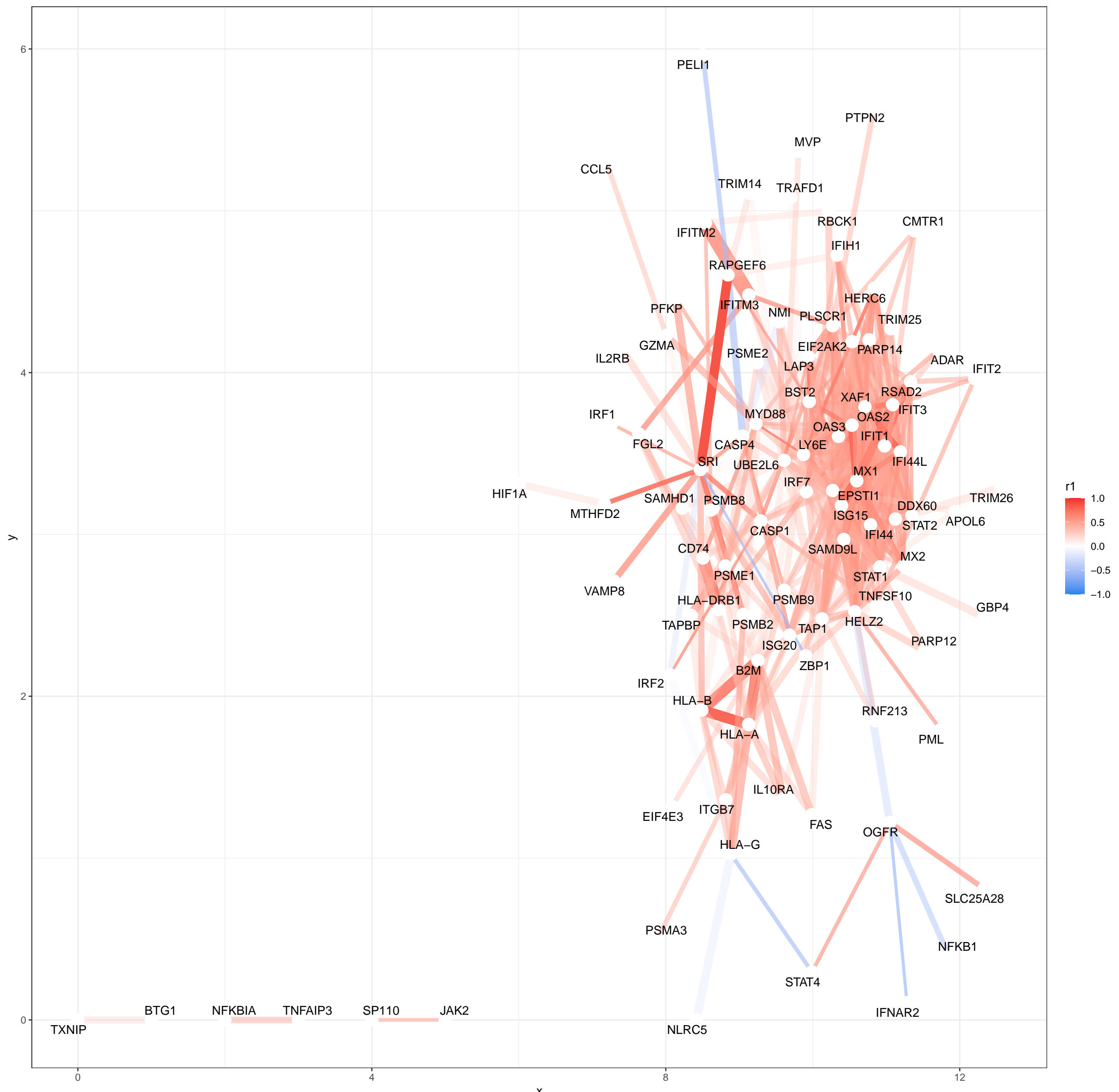

Healthy

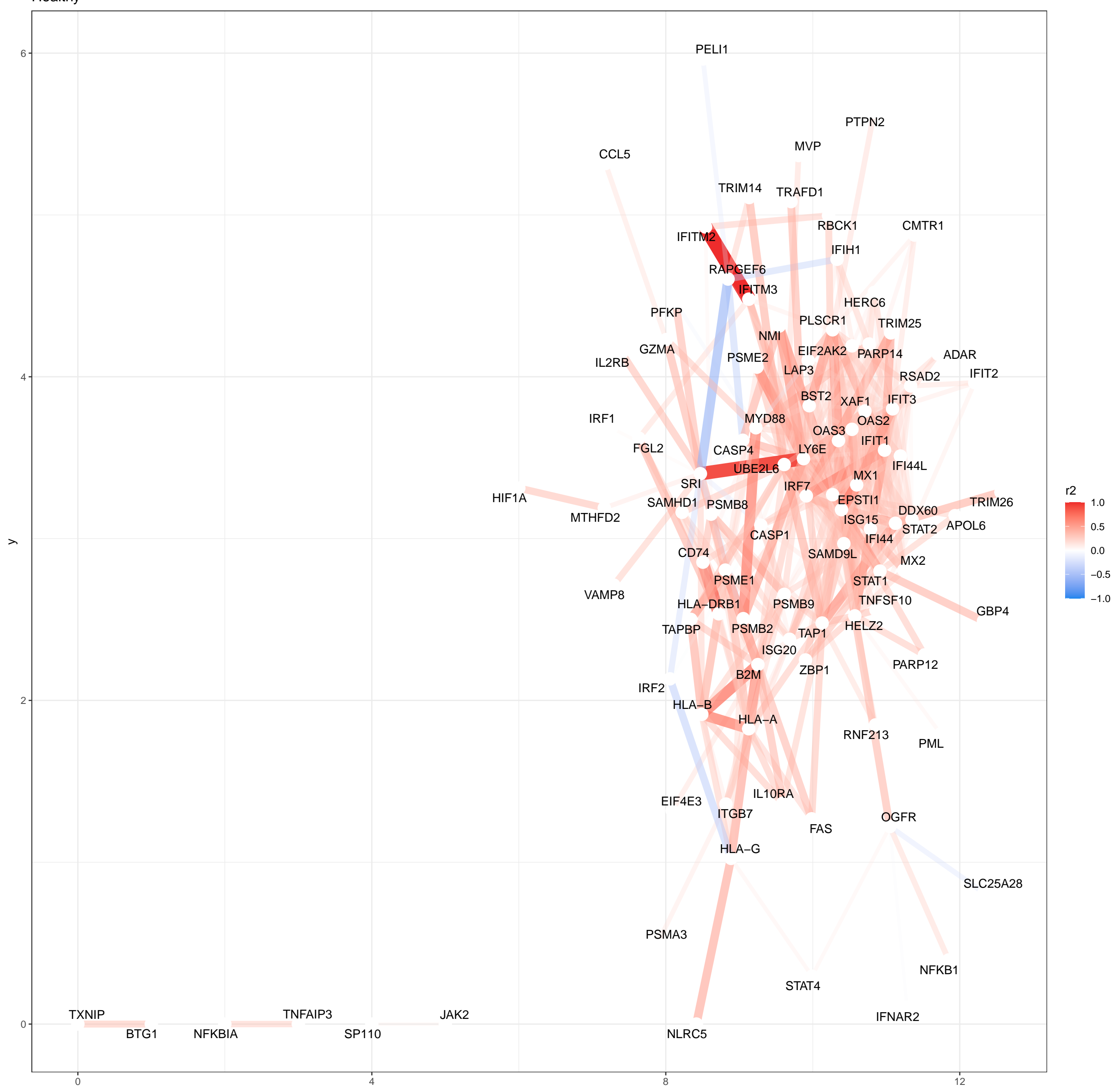

Difference (P1 - P2)

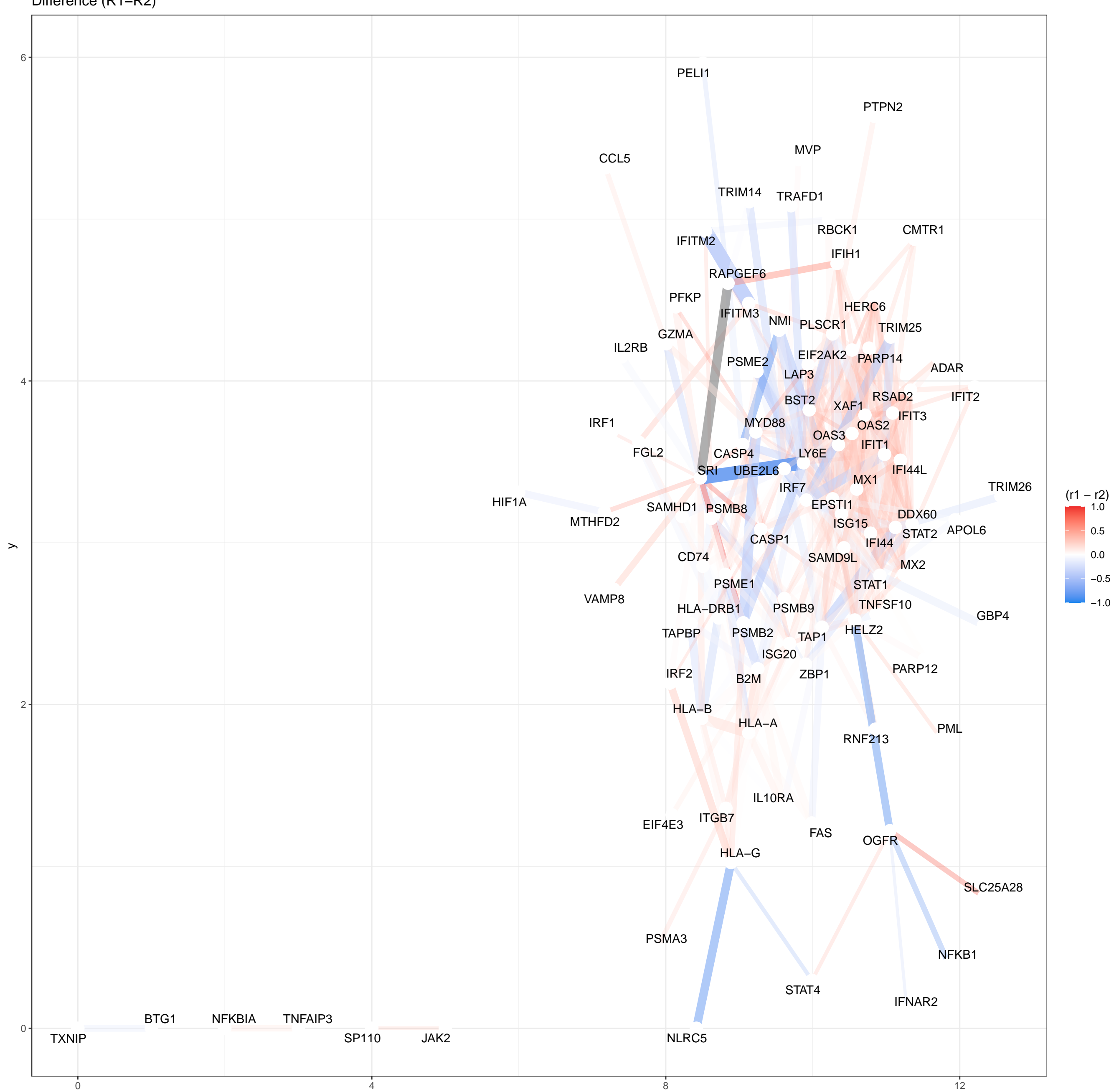

Supplement: Supplementary file 1 [file ijms-26-04771-s001.zip › File S2_Figure4_CD4_high_res_1200.pdf]
